# *let-7* MicroRNA-Mediated Regulation of Shh Signaling and the Gene Regulatory Network Is Essential for Retina Regeneration

**DOI:** 10.1016/j.celrep.2018.04.002

**Published:** 2018-05-02

**Authors:** Simran Kaur, Shivangi Gupta, Mansi Chaudhary, Mohammad Anwar Khursheed, Soumitra Mitra, Akshai Janardhana Kurup, Rajesh Ramachandran

**Affiliations:** 1 Department of Biological Sciences, Indian Institute of Science Education and Research, Mohali, Knowledge City, SAS Nagar, Sector 81, Manauli PO, 140306 Mohali, Punjab, India

**Keywords:** zebrafish, retina, regeneration, Shh, Ascl1a, Mmp9, Zic2b, Foxn4, *let-7*, Lin28

## Abstract

Upon injury, Müller glia cells of the zebrafish retina reprogram themselves to progenitor cells with stem cell characteristics. This necessity for retina regeneration is often compromised in mammals. We explored the significance of developmentally inevitable Sonic hedgehog signaling and found its necessity in MG reprogramming during retina regeneration. We report on stringent translational regulation of *sonic hedgehog*, *smoothened*, and *patched1* by *let-7* microRNA, which is regulated by Lin28a, in Müller glia (MG)-derived progenitor cells (MGPCs). We also show Shh-signaling-mediated induction of Ascl1 in mouse and zebrafish retina. Moreover, Shh-signaling-dependent regulation of *matrix metalloproteinase9*, in turn, regulates Shha levels and genes essential for retina regeneration, such as *lin28a*, *zic2b*, and *foxn4*. These observations were further confirmed through whole-retina RNA-sequencing (RNA-seq) analysis. This mechanistic gene expression network could lead to a better understanding of retina regeneration and, consequently, aid in designing strategies for therapeutic intervention in human retinal diseases.

## Highlights

• Shh signaling is essential for MG dedifferentiation during retina regeneration
• Shh signaling components are regulated by *let-7* microRNA in the zebrafish retina
• A regulatory feedback loop between Mmp9 and Shh signaling is active in the retina
• Shh signaling induced a gene-regulatory network involving *mmp9*, *ascl1a*, *zic2b*, and *foxn4*

## Graphical Abstract

**Figure:**
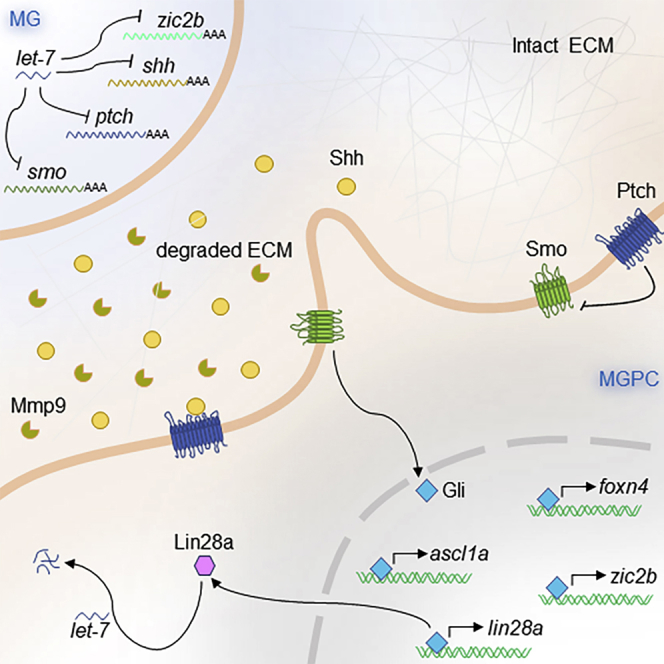

## Teaser

Kaur et al. demonstrate that microRNA *let-7* in injured zebrafish retina regulates the translation of *shha*, *shhb*, *smo*, *ptch1*, and *zic2b* mRNAs. Further, Shh signaling is necessary during retina regeneration for inducing a pro-regenerative gene expression cascade involving several genes, including *ascl1a*, *lin28a*, *mmp9*, *foxn4*, and *zic2b*.

## Introduction

In contrast to mammals, zebrafish retina possesses remarkable regenerative capacity after an acute injury, leading to functional restoration of vision (Sherpa et al., 2008). The Müller glia (MG) cells in zebrafish retina reprogram themselves to MG-derived progenitor cells (MGPCs) that systematically differentiate into all retinal neurons, namely rods, cones, horizontal, amacrine, ganglion, bipolar cells, and MG itself (Ramachandran et al., 2010b). Although induction of MGPCs immensely contributes to the successful regeneration of zebrafish retina, the complete mechanism remains elusive. While the mechanism of retina regeneration is histologically well described, only a subset of the involved genes/proteins has been identified and characterized functionally (Goldman, 2014, Wan and Goldman, 2016). Therefore, we attempted to identify previously uncharacterized regulators of zebrafish retina regeneration using the needle-poke method of injury, which reflects the situation of mechanical damage that occurs in nature.

Even though several studies have elucidated the importance of Delta-Notch, Wnt, and Fgf signaling during retina regeneration in zebrafish, the roles of developmentally important Shh signaling remain largely underexplored (Goldman, 2014, Sun et al., 2014, Wan and Goldman, 2016). Recent studies have revealed the potential roles of Shh signaling during tissue regeneration (Ando et al., 2017, Dunaeva and Waltenberger, 2017, Thomas et al., 2018, Todd and Fischer, 2015). Therefore, we investigated the mechanistic involvement of Shh signaling during zebrafish retina regeneration. Subsequently, we hypothesized that MG dedifferentiation may depend on Shh signaling and have some similarities to the reprogramming of somatic cells by pluripotency-inducing factors (Hochedlinger and Plath, 2009, van den Hurk et al., 2016). Since we were interested in the possible involvement of Shh signaling during the early regenerative response of MG to injury, we analyzed the retina within the first few days after blockade of Shh signaling. We identified expression pattern of several important genes induced by Shh signaling and vice versa that reveal the robust regulatory network associated with retina regeneration. These include the interplay of Shh/Notch signaling components, transcription factors (namely, Ascl1a, Zic2b, Foxn4, and Insm1a), the matrix metalloproteinase Mmp9, the RNA-binding protein Lin28a, and microRNA *let-7*. Complete retina regeneration in zebrafish has provided valuable clues as to why their mammalian counterparts often fail (Goldman, 2014, Wan and Goldman, 2016). The findings from this study add clarity to the enigmatic process of retina regeneration lacking in mammals.

## Results

### Injury-Dependent Induction of Shh Signaling Is Essential for Regeneration

**Figure 1:**
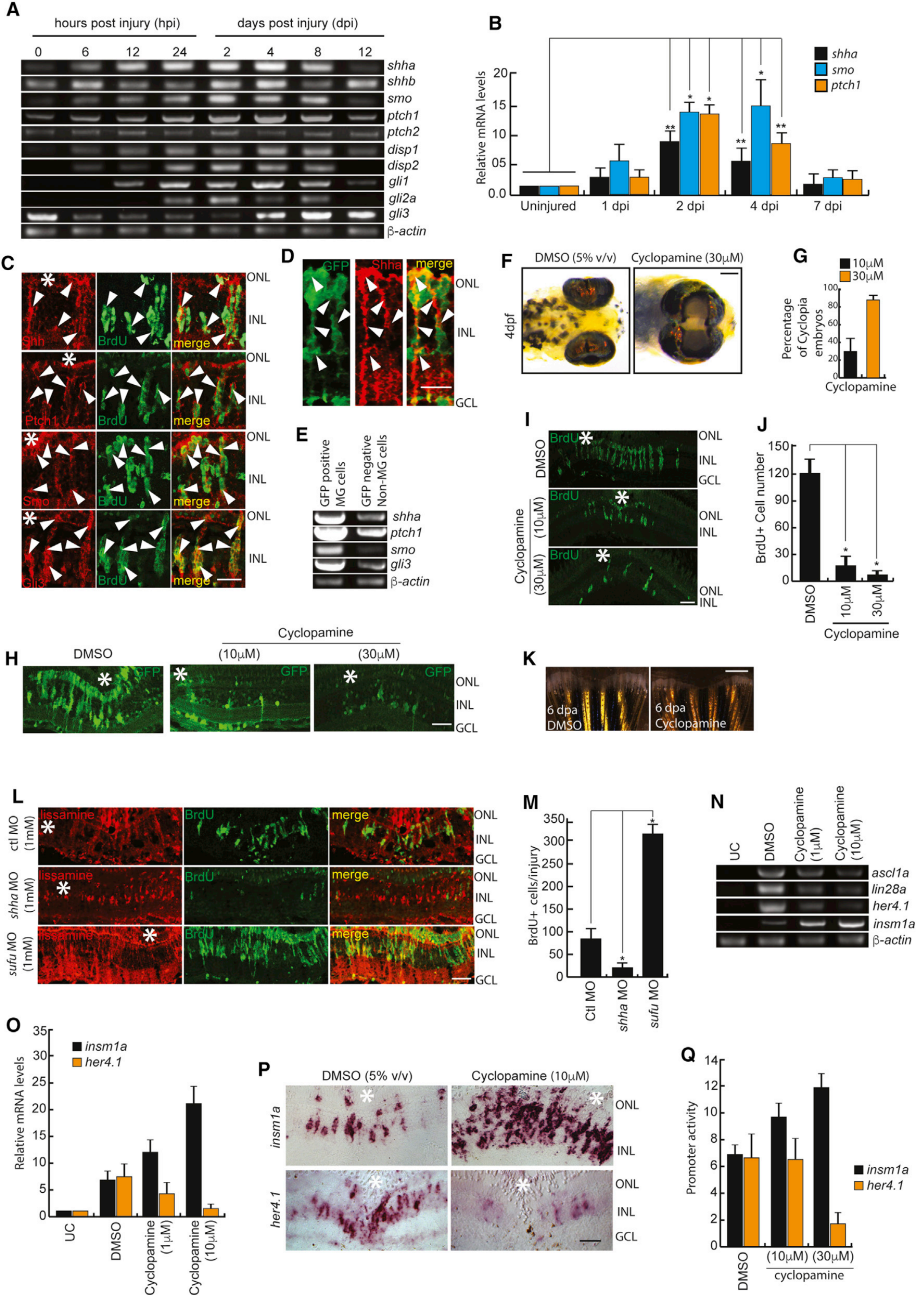
Shh Signaling Is Necessary for MG Dedifferentiation in the Injured Retina (A and B) RT-PCR (A) and qPCR (B) analysis of Shh signaling component genes in the retina at indicated time points post-injury; n = 6 biological replicates. ^∗^p < 0.001; ^∗∗^p < 0.003. (C and D) Immunofluorescence (IF) microscopy images of Shh signaling components in wild-type BrdU^+^ MGPCs (C), and Shh expression in *1016 tuba1a:*GFP transgenic fish at 4 dpi (D). Arrowheads mark protein expression in cells in (C) and (D). (E) RT-PCR assay of Shh signaling component genes in GFP-positive MGPCs and the rest of the cells from *1016 tuba1a*:GFP transgenic retina at 4 dpi. (F and G) Bright-field (BF) images of 4-days post-fertilized embryos treated with 5% (v/v) DMSO and 30 μM cyclopamine (F), and quantification of the number of cyclopia embryos (G). (H–J) IF microscopy images showing a dose-dependent decline in GFP^+^ and BrdU^+^ MGPCs in *1016 tuba1a:*GFP transgenic (H) and wild-type (I) retinae, respectively, at 4 dpi upon cyclopamine treatment, which is quantified in (J). (K) BF microscopy images of blastema during caudal fin regeneration in cyclopamine-treated wild-type zebrafish at 6 days post-amputation. (L and M) IF microscopy images of retinal sections with *shha* or *sufu* knockdowns (L), and quantification of the number of BrdU^+^ cells at the injury site (M). ^∗^p < 0.0001; n = 4 biological replicates. Lissamine tag on MO shows red fluorescence in (L). (N–P) RT-PCR analysis of *ascl1a, lin28a, her4.1*, and *insm1a* in uninjured control, 2.5 dpi DMSO-treated, and 2.5 dpi cyclopamine-treated retina (N); qPCR analysis of mRNA levels of *insm1a* and *her4.1* with cyclopamine treatment (O); and BF images of corresponding mRNA *in situ* hybridization (ISH) of these genes in the retina at 4 dpi (P). (Q) Single-cell-stage embryos were injected with *insm1a:luciferase* or *her4.1:luciferase* vectors along with Renilla luciferase mRNA for normalization and then treated with cyclopamine for 24 hr before lysing for quantification of *insm1a* and *her4.1* promoter activity using a dual luciferase assay. Scale bars represent 10 μm in (C), (D), (H), (I), (L), and (P) and 500 μm in (F) and (K). Asterisk indicates the injury site (C, H, I, L, and P). Error bars represent SD. ^∗^p < 0.0001 (J); ^∗^p < 0.001 (M). n = 6 biological replicates. GCL, ganglion cell layer; INL, inner nuclear layer; ONL, outer nuclear layer; UC, uninjured control. See also Figures S1, S2, S6, and S7.

We explored the temporal expression pattern of Shh signaling component genes such as *sonic hedgehog* (*shha*, *shhb*), smoothened (*smo*), *patched1* (*ptch1*), *patched2* (*ptch2*), *dispatched1* (*disp1*), *dispatched2* (*disp2*), and *glioma-associated oncogene* (*gli1*, *gli2a*, and *gli3*) in total retina. We found that most of these genes were upregulated after retinal injury, except *gli3*, which showed a downregulation (Figures 1A and 1B). Moreover, the Shh signaling components Shh, Ptch1, Smo, and Gli3 showed co-localization with bromodeoxyuridine (BrdU)^+^ MGPCs (Figures 1C, S1A, S1B, and S7A). Western blot analysis revealed a temporal upregulation of Shh protein with a peak of expression at 4 days post-injury (dpi) (Figures S1C and S7A). The Shh protein is expressed in MG cells of wild-type (WT) injured retina marked by glutamine synthetase (GS) at 4 dpi (Figure S1D). Using *tuba1a1016*:GFP transgenic zebrafish (Fausett and Goldman, 2006), we showed the expression of Shh and its signaling components in proliferating MGPCs marked by GFP. Immunofluorescence (IF) studies and cell sorting revealed a relative abundance of Shh protein and its signaling components in GFP^+^ MGPCs compared with the rest of the cells of *tuba1a1016*: GFP transgenic retina at 4 dpi (Figures 1D and 1E). We confirmed the secretion of Shha and its probable autocrine action in MG using brefeldin A, a protein transport inhibitor, (Miller et al., 1992) and observed an expected increase in intracellular Shha and a decline in BrdU^+^ cells (Figures S1E and S1F).

To decipher the influence of Shh signaling on retina regeneration, we used the pharmacological agent cyclopamine (Incardona et al., 1998), a potent inhibitor of Smo (Chen et al., 2002). We found that at 30 μM concentration, 90% of zebrafish embryos exhibited cyclopia, a hallmark of impaired Shh signaling, which also impacted developmentally important genes (Figures 1F, 1G, and S1G). We then explored the impact of continuous cyclopamine exposure on MGPC induction and regeneration in WT and *tuba1a1016*:GFP transgenic retina at 4 dpi. Interestingly, 10 μM and 30 μM concentrations significantly inhibited MGPC induction (Figures 1H–1J, S1H, and S1I), which was not the result of enhanced apoptosis (Figure S1J). A similar reduction in fin blastema was also seen with cyclopamine treatment on the 6^th^ day post-amputation (Figure 1K), suggesting a conserved Shh signaling mechanism across tissues during regeneration. The few residual BrdU^+^ MGPCs in cyclopamine-treated retina failed to form any retinal cell types (Figure S1K). Moreover, morpholino (MO)-based targeted gene knockdown of Shh signaling component genes such as *shha*, *shhb*, *ptch1*, *ptch2*, and *gli2a* caused progenitor reduction, and that of negative regulators *sufu* (*suppressor of fused*) (Figures 1L and 1M and S2A–S2C) and *gli3* (Figures 5I, S6A, and S6B; Table S1) enhanced MGPC induction as compared with control retina at 4 dpi. These increased MGPCs when traced until 20 dpi revealed the formation of amacrine, bipolar, and MG cells, indicating their functional potential to give rise to different retinal cell types (Figures S2D and S2E). These results emphasize the importance of Shh signaling during retina regeneration.

We also performed whole-retina RNA sequencing (RNA-seq) at 12 hr post-injury (hpi), 4 dpi, and 4 dpi with cyclopamine treatment compared with uninjured controls to get a holistic view of the blockade of Shh signaling. We found that several transcription factor genes, including *ascl1a*, *zic2b*, *foxn4*, and matrix metalloproteinase *mmp9*, are regulated with cyclopamine treatment (Table S3; Figures S1L and S1M; GEO: GSE102063).

### Shh Signaling Affects Expression of Repressor Genes

We then explored the impact of compromised Shh signaling in the expression pattern of well-known regeneration-associated repressor genes such as *her4.1* and *insm1a* (Goldman, 2014). RT-PCR and qPCR analysis in cyclopamine-treated retina revealed that the pivotal regeneration-associated genes are downregulated, with the exception of *insm1a* and a few Notch signaling genes (Figures 1N, 1O, and S2F). Insm1a, a known transcriptional repressor in MGPC induction and cell-cycle exit (Ramachandran et al., 2012, Zhang et al., 2009), showed upregulation, whereas levels of *her4.1*, one of the effectors of Notch signaling (Pasini et al., 2004, Wilson et al., 2016), showed downregulation, which was confirmed by mRNA *in situ* hybridization (ISH) and luciferase assays (Figures 1P and 1Q). Upregulation of *insm1a* and downregulation of *her4.1* with blocked Shh signaling in post-injured retina led us to hypothesize the involvement of a well-known transcription factor such as Ascl1a in this regulatory loop. Insm1a, a known transcriptional repressor of *ascl1a* (Ramachandran et al., 2012), could influence its expression in a Shh-signaling-dependent manner. Moreover, Ascl1a could impact the expression of *delta* genes (Henke et al., 2009, Nelson et al., 2009), the ligand of Notch signaling, capable of inducing *her4.1* expression in Notch-expressing cells (Takke et al., 1999). Thus, the Shh-signaling-dependent increase in Insm1a could cause a downregulation of *ascl1a*, which in turn reduces *her4.1* levels in injured retina. These results suggest possible crosstalk between Shh and Notch signaling, contributing to retina regeneration.

### Shh Signaling Induces *ascl1a* during Retina Regeneration

**Figure 2:**
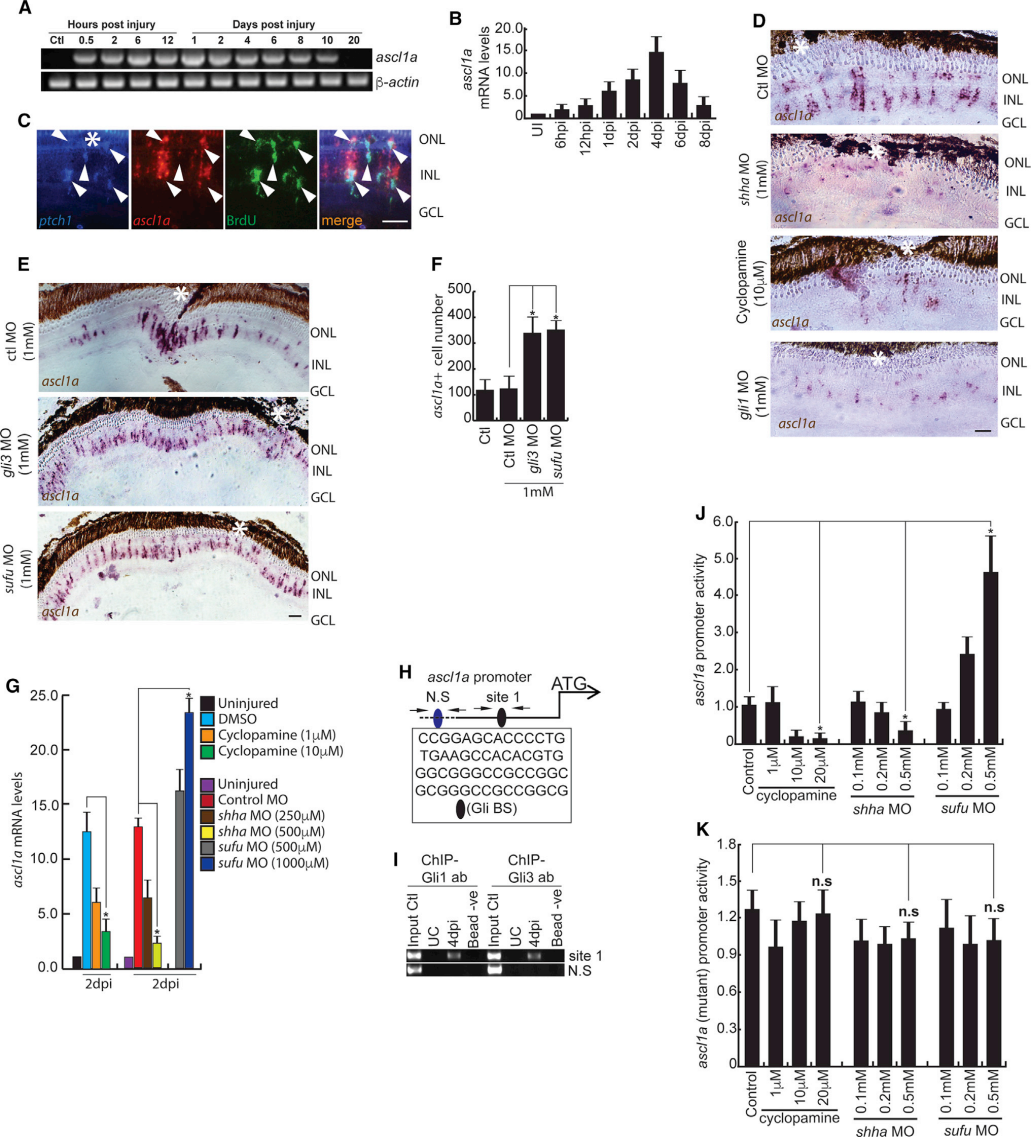
Shh-Signaling-Dependent *ascl1a* Regulation in the Injured Retina (A and B) RT-PCR (A) and qPCR (B) analysis of *ascl1a* in the post-injured retina; n = 6 biological replicates. (C) Fluorescence ISH (FISH) and IF microscopy images of a 0.5-μm-thick optical section of retina showing co-localization of *ascl1a* with *ptch1* in BrdU^+^ MGPCs at 4 dpi. Arrowheads mark co-expression of genes in BrdU^+^ cells. (D–F) BF microscopy images of *ascl1a* mRNA ISH in retina at 4 dpi with cyclopamine treatment, *shha* or *gli1* knockdowns (D), and *gli3* or *sufu* knockdowns (E). The number of *ascl1a*^+^ cells from (E) is quantified in (F). (G) qPCR analysis of *ascl1a* mRNA with cyclopamine treatment and *shha* or *sufu* knockdown in 2 dpi retina. (H) Schematic of the *ascl1a* promoter with a putative Gli-binding site (Gli-BS) cluster. Arrows mark ChIP primers, N.S marks the negative control, and capital letters mark putative Gli-BSs. (I) Retinal ChIP assay at 4 dpi showing both Gli1 and Gli3 bound to the *ascl1a* promoter. (J) Luciferase assay in 24 hpf embryos co-injected with *ascl1a:*GFP*-luciferase* vector and *sufu* or *shha* MOs. (K) Luciferase assay was done with mutated Gli-BS of *ascl1a* promoter in an experiment similar to (J). Scale bars represent 10 μm in (C) and 20 μm in (D) and (E). Asterisk indicates the injury site (C–E). Error bars represent SD. ^∗^p < 0.0001 (F); ^∗^p < 0.01 (G); ^∗^p < 0.01 (J). n = 6 biological replicates (F and G); n = 3 (J). See also Figures S2, S6, and S7.

Apart from the potential involvement of Insm1a in repressing *ascl1a* levels, we also speculated its direct regulation mediated through Shh signaling. This is presumably true, as the temporal expression pattern of *ascl1a* by RT-PCR and qPCR matched that of Shh signaling components (Figures 1A, 1B, 2A, and 2B). We found the co-expression of *ptch1*, a bona fide marker of active Shh signaling (Jeong and McMahon, 2005), with *ascl1a* mRNA in retina at 4 dpi (Figure 2C). This suggests the potential involvement of Shh signaling in *ascl1a* induction and vice versa. Inhibition of Shh signaling, by cyclopamine treatment or knockdown of *gli1* or *shha*, significantly downregulated *ascl1a* expression (Figures 1N and S2G), which was also confirmed by mRNA ISH and qPCR in retina (Figures 2D and 2G). Conversely, knockdown of negative regulators of Shh signaling, *gli3* and *sufu*, caused an upregulation of *ascl1a* (Figures 2E–2G), suggesting its possible direct regulation. This is supported by the presence of several Gli-binding sites on the *ascl1a* promoter, revealed by *in silico* analysis (Figure 2H). Further, we performed a post-injured retinal chromatin immunoprecipitation (ChIP) assay using antibodies against the Shh signaling effector proteins Gli1 and Gli3 separately to examine whether these Gli-binding sites (Gli-BSs) are functional. Interestingly, both antibodies could separately precipitate Gli-bound chromatin, supporting the direct physical interaction of Gli1/Gli3 on the *ascl1a* promoter (Figures 2I and S2K). Furthermore, a luciferase assay performed in zebrafish embryos confirmed the effect of stimulators and inhibitors of Shh signaling on *ascl1a* expression (Figure 2J). The Gli-BS mutations in the *ascl1a* promoter almost completely abolished the effect of inhibitors and stimulators as revealed by the luciferase assay (Table S2; Figure 2K). These results suggest that Shh signaling regulates the important gene *ascl1a*.

### Shh Signaling/*lin28a*/*let-7* Regulatory Loop Is Essential for MGPC Induction

**Figure 3:**
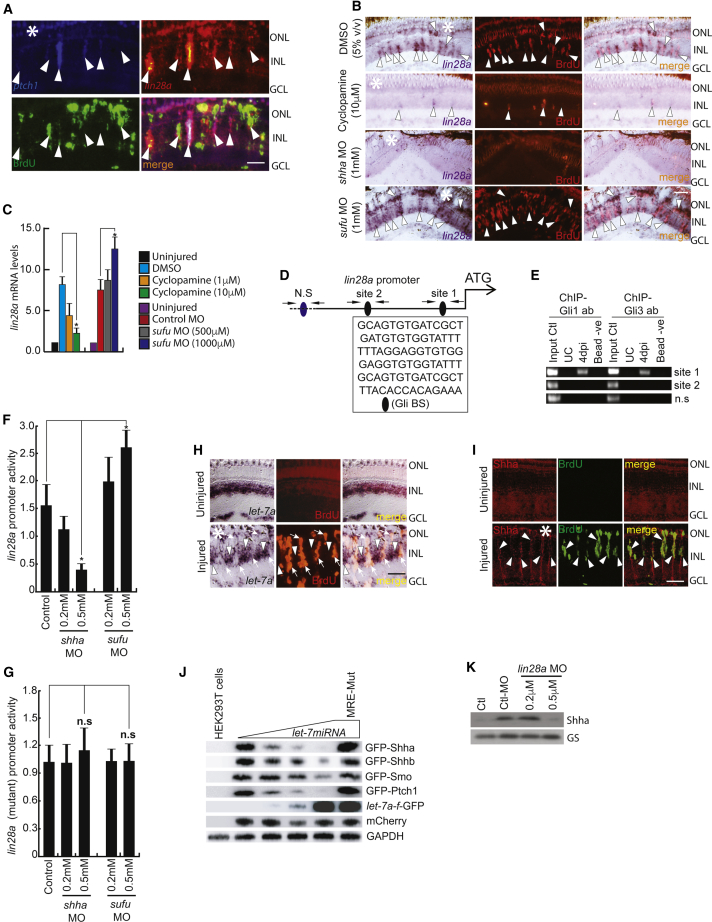
Lin28a-*let-7* Axis Regulates Shh Signaling Component Genes in the Injured Retina (A) FISH and IF microscopy images of a 0.5-μm-thick optical section of retina showed co-localization of *lin28a* with *ptch1* in BrdU^+^ MGPCs at 4 dpi. Arrowheads mark co-expression of genes in BrdU^+^ cells. (B and C) BF microscopy images of *lin28a* mRNA ISH in the retina at 4 dpi with cyclopamine treatment and *shha* or *sufu* knockdown (B), which was quantified by qPCR (C). Arrowheads mark co-expression of genes in BrdU^+^ cells in (B). (D and E) Schematic of the *lin28a* promoter with a potential Gli-BS cluster, where arrows mark ChIP primers and capital letters mark consensus sequence of Gli-BS (D). A 4 dpi retinal ChIP assay showed both Gli1 and Gli3 bound to one of the two Gli-BS clusters (E). (F) Luciferase assay in 24 hpf embryos co-injected with *lin28a:*GFP*-luciferase* vector and *sufu* or *shha* MOs. (G) Luciferase assay with mutated Gli-BSs of the *lin28a* promoter in an experiment similar to (F). (H and I) ISH and IF microscopy of retina showing co-exclusion of *let-7a* microRNA (H) and co-localization of Shha protein (I) in BrdU^+^ MGPCs in the retina at 4 dpi. Arrowheads mark expression of *let-7a* in BrdU^−^ cells and arrows mark co-exclusion of *let-7a* from BrdU^+^ cells in (H). Arrowheads mark co-expression of Shha in BrdU^+^ cells in (I). (J) *let-7* microRNA downregulated the translation of GFP fused with the indicated gene constructs harboring microRNA-binding regions in a dose-dependent manner in HEK293T cells. (K) Western blot of Shha in *lin28a*-MO electroporated retina at 4 dpi. Scale bars represent 10 μm (A, H, and I) and 20 μm (B). Asterisk indicates the injury site (A, B, H, and I). Error bars represent SD.^∗^p < 0.001 (C); ^∗^p < 0.001 (F). n = 6 biological replicates (C, F, and G). GS, glutamine synthetase. See also Figures S3, S6, and S7.

We then explored whether the RNA-binding protein and pluripotency-inducing factor Lin28a, a necessary and well-known target of Ascl1a during retina regeneration, is regulated directly through Shh signaling (Ramachandran et al., 2010a). This was supported by the co-expression of *ptch1* and *lin28a* in 4 dpi retinal sections (Figure 3A), suggesting the possible interdependency or hierarchical regulation. We further evaluated the expression pattern of *lin28a* that goes down with inhibited Shh signaling in retinal cross sections (Figure 3B). This was also proven by qPCR (Figure 3C). The opposite expression pattern of *lin28a* was found with *sufu* knockdown, as expected (Figures 3B and 3C). Evaluation of the *lin28a* promoter revealed putative Gli-BSs (Figure 3D) located as clusters, which were probed using Gli1 and Gli3 antibodies for a ChIP assay in the post-injured retina. Interestingly, both Gli1 and Gli3 bind to one of these Gli-BS clusters (Figures 3E and S2K), suggesting direct regulation of *lin28a* by Gli proteins. These results were further confirmed by luciferase assay performed in zebrafish embryos co-injected with *lin28a:*GFP-luciferase vector along with MOs against positive and negative regulators of Shh signaling (Figure 3F). The introduction of Gli-BS mutations in the *lin28a* promoter alleviated the impact of inhibitors and stimulators as revealed by a luciferase assay (Table S2; Figure 3G). Furthermore, *let-7* microRNA, which is downregulated by Lin28a (Ramachandran et al., 2010a), was abundant in the uninjured inner nuclear layer (INL) in BrdU^+^ MGPCs at 4 dpi (Figure 3H). This *let-7* downregulation in MGPCs is opposite to the IF pattern of Shh (Figures 3H and 3I), which suggested possible regulation of *shha* mRNA by *let-7* microRNA. The mRNA ISH of *shha* and *ptch1* also revealed a diffused expression pattern in both uninjured and 4 dpi retina (Figures S2H–S2J). *In silico* analysis predicted several *let-7* microRNA-binding sites present in *shha*, *shhb*, *smo*, and *ptch1* genes (Table S4). We cloned these four genes in-frame with GFP reporter regulated by the cytomegalovirus (CMV) promoter and transfected these constructs with increasing concentrations of *let-7a* and *let-7f* microRNA expression plasmid (Ramachandran et al., 2010a) in HEK293T cells (Figure S5F). The results showed a dose-dependent decline in GFP expression (Figure 3J), which was quantified (Figures S6C–S6F). The knockdown of *lin28a* led to an expected decline in Shha protein at 4 dpi (Figure 3K). These findings suggest that *lin28a*-mediated suppression of *let-7* is required for the translational regulation of Shh signaling components in MGPCs as a part of positive feedback loop mediated through the Ascl1a-*lin28a* axis.

### Mmp9 Regulates *ascl1a* through Shh Signaling

**Figure 4:**
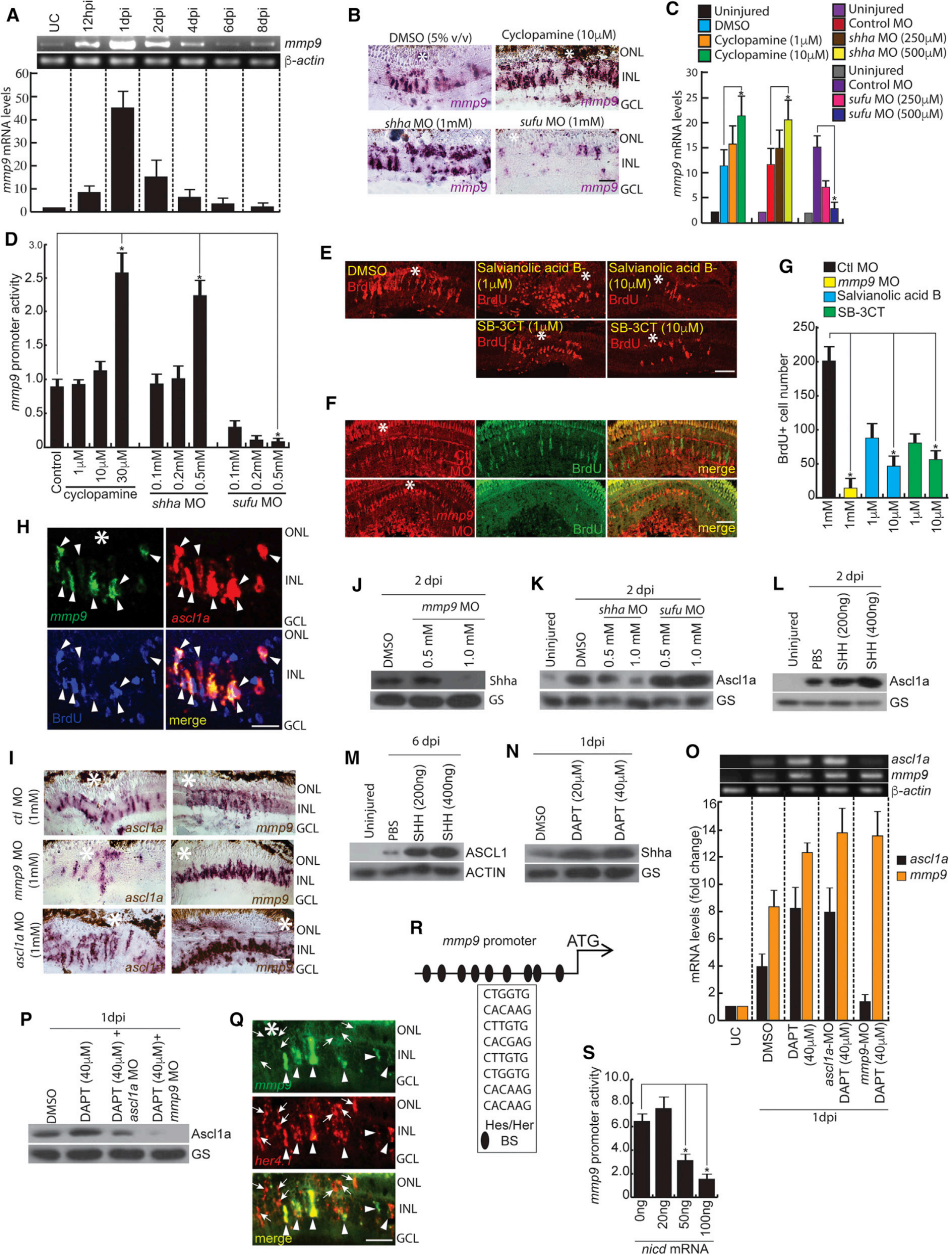
Shh-Mmp9-Ascl1a Interplay Is Necessary during MG Reprogramming (A) RT-PCR (top) and qPCR (bottom) analysis of injury-dependent *mmp9* expression in the retina; n = 6 biological replicates. (B–D) BF microscopy images of *mmp9* mRNA ISH in the retina at 4 dpi with cyclopamine treatment and *shha* or *sufu* knockdown (B), as quantified by qPCR (C), and a luciferase assay in 24 hpf embryos injected with *mmp9:*GFP*-luciferase* vector (D). (E–G) IF microscopy images of 4 dpi retina with Mmp9 blockade using drugs (E) and MO against *mmp9* (F). The number of BrdU^+^ MGPCs is quantified in (G). (H) FISH and IF microscopy images of a 0.5-μm-thick optical section of retina showing co-localization of *mmp9* and *ascl1a* in BrdU^+^ MGPCs at 4 dpi. Arrowheads mark co-expression of genes in BrdU^+^ cells. (I) BF microscopy images of *ascl1a* and *mmp9* mRNA ISH in *ascl1a* and *mmp9* knockdowns in 4 dpi retina. (J) Western blotting experiment showing Shh levels in 2 dpi retina with the *mmp9* knockdown. (K) Western blotting assay of Ascl1a in 2 dpi retina with *shha or sufu* knockdowns. (L) Western blotting assay of Ascl1a in 2 dpi zebrafish retina injected with recombinant SHH protein. (M) Western blotting assay of ASCL1 in 6 dpi mouse retina injected with recombinant SHH protein. (N) Western blotting assay of Shha in DAPT-treated retina at 1dpi. (O and P) RT-PCR (top) and qPCR (bottom) analysis of *ascl1a* and *mmp9* in DAPT-treated retina, with or without *ascl1a* or *mmp9* knockdown (O), and confirmed by western blotting assay (P). (Q) FISH and IF microscopy images of a 0.5-μm-thick optical section of retina showed substantial co-exclusion and marginal co-localization of *mmp9* with *her4.1* at 4 dpi. Arrowheads mark co-expression of the gene, and arrows mark *her4.1*^+^ cells. (R and S) Schematic of the *mmp9* promoter with potential Hes/Her-BS binding sites (inside box), and luciferase assay in 24 hpf embryos co-injected with *mmp9:*GFP*-luciferase* construct and notch intracellular domain (*nicd*) mRNA (S). Scale bars represent 10 μm (H and Q) and 20 μm (B, E, F, and I). Asterisk indicates the injury site (B, E, F, H, I and Q). Error bars represent SD. ^∗^p < 0.001 (C, D, G, and S). Biological replicates n = 6 in (C) and (G), and n = 3 in (D) and (S). See also Figures S3, S4, S6, and S7.

We also investigated the involvement of *mmp9*, a gene highly induced in regenerating MG cells, as revealed in microarray analysis (Ramachandran et al., 2012) and whole-retina RNA-seq done in the present study. Mmp9 is not only an important enzyme prerequisite for proliferative and pro-differentiative roles (Mannello et al., 2006), but also essential during fin regeneration (LeBert et al., 2015, Yoshinari et al., 2009). We found that *mmp9* is rapidly induced in the injured retina, with a peak expression at 24 hpi (Figures 4A and S3A), and later (at 4 dpi), *mmp9* levels were restricted to the neighboring cells of BrdU^+^ MGPCs (Figures S3B and S3C). Interestingly, inhibition of Shh signaling caused a significant upregulation of *mmp9*, and an opposite effect was seen with *sufu* knockdown (Figures 4B and S3D–S3F), which was confirmed by qPCR (Figure 4C) and a luciferase assay performed in zebrafish embryos injected with *mmp9*:GFP-luciferase vector (Figure 4D). These results suggest a negative correlation between *mmp9* and active cell proliferation. However, upon inhibition of Mmp9 using pharmacological agents such as salvianolic acid B and SB-3CT, or by *mmp9* targeting MO (Figures S6A, and S6B; Table S1), we found a drastic decline in BrdU^+^ cells in WT or GFP^+^ cells in *tuba1016* transgenic retina (Figures 4E–4G and S3G). Interestingly, no impact was seen with *mmp9* blockade after 2 dpi (Figure S3H), suggesting that its role preludes cell proliferation. To evaluate this further, we analyzed the expression pattern of an important gene, *ascl1a*, in *mmp9*-expressing cells in 4 dpi retina. We found significant co-localization of *ascl1a*^+^ cells with *mmp9* expression (Figures 4H and S4A). Moreover, *mmp9* knockdown caused a decline in *ascl1a* expression, whereas *ascl1a* knockdown caused an upregulation of *mmp9* in 4 dpi retina (Figures 4I and S3I). Since the regulation of *ascl1a* is established through Shh signaling, we further explored whether Mmp9-mediated regulation of *ascl1a* was through Shha. Knockdown of *mmp9* abolished the expression of Shha, as found with cyclopamine treatment (Figures 4J, S3J, and S7B). We also found an Shh-signaling-dependent regulation of Ascl1a protein with both *shha* or *sufu* knockdowns in 2 dpi retina (Figures 4K and S7C). Recombinant-SHH could induce Ascl1a expression and cell proliferation in zebrafish retina, similar to *sufu* knockdown (Figures 4L, S3K–S3M, and S7D). Interestingly, we also found a drastic increase in mRNA levels of *Ascl1*, *Lin28a*, and ASCL1 protein in injured mouse retina treated with recombinant-SHH (Figures 4M, S3N, and S7E).

Inhibition of Notch signaling through *N*-[*N*-(3,5-difluorophenylacetyl)-L-alanyl]-*S*-phenylglycine *t*-butyl ester (DAPT) treatment, which causes a decline in Her4.1 levels and enhancement of MGPCs during retina regeneration (Conner et al., 2014, Wan et al., 2012), increased *mmp9, ascl1a* mRNA, and Shh protein levels (Figures S4B, S4C, 4N, and S7F). We further explored whether *ascl1a* upregulation seen with DAPT treatment is mediated through the Mmp9/Shh axis. Interestingly, we found that in the DAPT-treated retina, *ascl1a* translation was nullified with *mmp9* knockdown (Figures 4O, 4P, and S7G). We speculated that upregulation of *mmp9* with blockade of Notch signaling is possibly due to a lack of Her4.1-mediated transcriptional repression. Expression of *mmp9* and *her4.1* showed co-labeling in a few and co-exclusion in the majority of retinal cells (Figure 4Q). *In silico* analysis of the *mmp9* promoter revealed several *hairy enhancer of split* (Hes/Her)-binding N-boxes (Kageyama et al., 2007), suggesting its potential regulation through Notch signaling (Figure 4R). We performed a luciferase assay in zebrafish embryos co-injected with notch intracellular domain (*nicd*) mRNA along with *mmp9*:GFP-luciferase vector. *nicd* mRNA could cause an upregulation of Her4.1 (Nakahara et al., 2016, Wilson et al., 2016), and the luciferase assay showed dose-dependent downregulation of *mmp9* promoter activity (Figure 4S), while mutations in Her4-binding sites abolished this impact (Figure S4D; Table S2). In summary, these results suggest that active Notch-signaling-mediated induction of *her4.1* restricts the span of *mmp9* expression at the site of injury. Further, Mmp9 coaxes MG to regenerate through Shh signaling and Ascl1a induction during retina regeneration.

### Shh Signaling Regulates *zic2b* Expression during Regeneration

**Figure 5:**
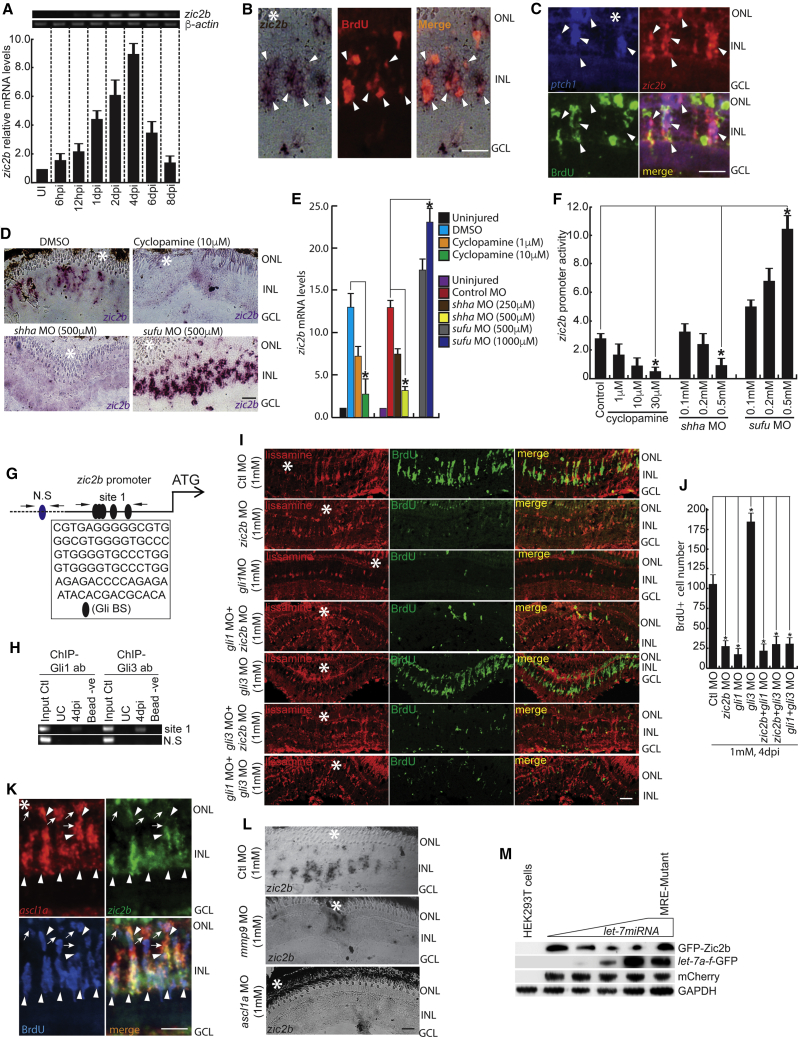
The Shh-Mediated Zic2b Axis Is Necessary during Retina Regeneration (A) RT-PCR (top) and qPCR (bottom) analysis of injury-dependent *zic2b* expression in the retina; n = 6 biological replicates. (B) ISH and IF microscopy revealed co-localization of *zic2b* mRNA with BrdU^+^ MGPCs in 4 dpi retina. (C) FISH and IF microscopy images of a 0.5-μm-thick optical section of retina showing co-localization of *zic2b* with *ptch1* in BrdU^+^ MGPCs at 4 dpi. (D and E) BF microscopy images of *zic2b* mRNA ISH in 4 dpi retina, with cyclopamine treatment, MO mediated *shha* or *sufu* knockdown done separately (D), which is quantified in (E). (F) Luciferase assay in 24 hpf embryos injected with *zic2b:*GFP*-luciferase* vector with cyclopamine treatment and *shha* or *sufu* knockdowns. (G) Schematic of the *zic2b* promoter with a putative Gli-BS. Arrows mark ChIP primers, N.S marks negative control devoid of Gli-BSs, and capital letters mark consensus of Gli-BSs. (H) Retinal ChIP assay at 4 dpi showing both Gli1 and Gli3 bound to the *zic2b* promoter. (I) IF microscopy images of BrdU^+^ cells in the regenerating retina with *zic2b*, *gli1*, and *gli3* knockdowns in isolation or combination, delivered at the time of injury, compared with control MO. (J) BrdU^+^ cells are quantified in the indicated knockdowns. (K) FISH and IF microscopy images of a 0.5-μm-thick optical section of retina showing co-localization of *zic2b* with *ascl1a* in BrdU^+^ MGPCs at 4 dpi. Arrowheads indicate *ascl1a* and *zic2b* co-expression, whereas arrows indicate *ascl1a*^+^ but *zic2b*^−^ cells. (L) ISH microscopy retinal images of *zic2b* mRNA with *mmp9* or *ascl1a* knockdown at 4 dpi. (M) *let-7* microRNA downregulated translation of the GFP construct appended with *zic2b* harboring microRNA responsive regions in a dose-dependent manner in HEK293T cells. Scale bars represent 10 μm (B, C, and K) and 20 μm (D, I, and L). Asterisk indicates the injury site (B, C, D, I, K, and L). Error bars represent SD. ^∗^p < 0.001 (E, F, and J). n = 6 biological replicates (E and J); n = 3 (F). See also Figures S4–S7.

We explored a zinc-finger transcription factor, Zic2, essential for normal brain patterning during development (Elms et al., 2003), which upon mutation shows holoprosencephaly (HPE) or cyclopia (Brown et al., 2001, Teslaa et al., 2013), a phenotype similar to cyclopamine treatment. Zic2 is also known to collaborate with Gli proteins (Koyabu et al., 2001). Therefore, we investigated whether a relationship exists between Gli proteins and Zic2 during retina regeneration, because both proteins occupy the same DNA sequence of the target genes’ promoters (Vokes et al., 2007). *zic2b*, orthologous to the mammalian Zic2 gene, showed upregulation in the retina microarray (Ramachandran et al., 2012) and our RNA-seq analysis. *zic2b* is also expressed in fin blastema (Figure S4E). The temporal expression pattern of *zic2b* in post-injured retina showed a peak expression at 4 dpi, a time when cell proliferation is at the maximum level (Figure 5A). Pulse labeling of MGPCs with BrdU also revealed its co-localization with *zic2b* (Figure 5B). Co-expression of *ptch1* with *zic2b* in BrdU^+^ cells suggests their interaction during regeneration (Figure 5C). The *zic2b* showed downregulation with blockade of Shh signaling and an upregulation with *sufu* knockdown (Figures 5D and 5E). These results were also confirmed by a luciferase assay done in zebrafish embryos injected with *zic2b*:GFP-luciferase construct along with MOs against *shha* and *sufu* and also exposed to cyclopamine (Figure 5F). Analysis of the *zic2b* promoter revealed a cluster of Gli-BSs (Figure 5G), and spanning chromatin was pulled down using both Gli1 and Gli3 antibodies separately (Figures 5H and S2K). Gene knockdowns of *gli1*, *gli3*, and *zic2b* significantly influenced MGPCs proliferation in 4 dpi retina (Figures 5I, 5J, S6A, and S6B; Table S1). The luciferase assay revealed that Shh signaling inhibitors and stimulators had a small impact on *zic2b* promoter activity with mutated Gli-BSs (Table S2; Figure S4F). Early or late knockdowns of *gli1*/*zic2b* caused a decline in the number of BrdU^+^ cells in the retina, but the opposite was seen with *gli3* knockdown (Figures 5I, 5J, S4G, and S4H). *zic2b* showed a pan retinal expression pattern with DAPT treatment, and the same was seen with *gli3*/*sufu* knockdowns (Figures S4I–S4K). Interestingly, *zic2b* knockdown nullified the enhancement of MGPCs with *gli3* knockdown (Figures 5I and 5J). Moreover, the induction of Gli3 seems to block the responsiveness of MGPCs to Gli1, as the late knockdowns and double knockdown of *gli1* and *gli3* also caused a drastic decline in cell proliferation (Figures 5I, 5J, S4G and S4H). The *gli1* knockdown significantly impacted several regeneration-associated genes as the possible cause of the lack of MGPC induction (Figure S4L). These results suggest that the induction of *zic2b* in MGPCs largely triggers a proliferative phase mediated through Shh signaling, and it may collaborate with or outcompete Gli proteins in targeting Gli-BSs to drive MGPCs toward differentiation.

We also examined whether *zic2b* expression depends on the *mmp9-shha-ascl1a* signaling axis, because a substantial proportion of BrdU^+^ MGPCs co-expressed *ascl1a* and *zic2b* (Figure 5K). We probed for *zic2b* expression in 4 dpi retina electroporated with *mmp9* and *ascl1a* MOs separately and found that *zic2b* levels declined drastically, as found with blockade of Shh signaling (Figures 5D and 5L). We further speculated that apart from its transcriptional control, *zic2b* might be regulated at translational levels. This speculation is mainly because of the presence of bona fide *let-7* microRNA-binding sites in the *zic2b* coding region (Figure S5F). Surprisingly, we found a downregulation in the translation of GFP protein from an expression cassette appended with *zic2b* in HEK293T cells (Figure 5M), which was quantified (Figure S6G). These results suggest that *zic2b* is an essential regeneration-associated gene in zebrafish retina that is regulated through the *mmp9-shha-ascl1a-lin28a-let-7* pathway.

### The Foxn4/Ascl1a/Shh/Zic2b Regulatory Loop Is Associated with Regeneration

**Figure 6:**
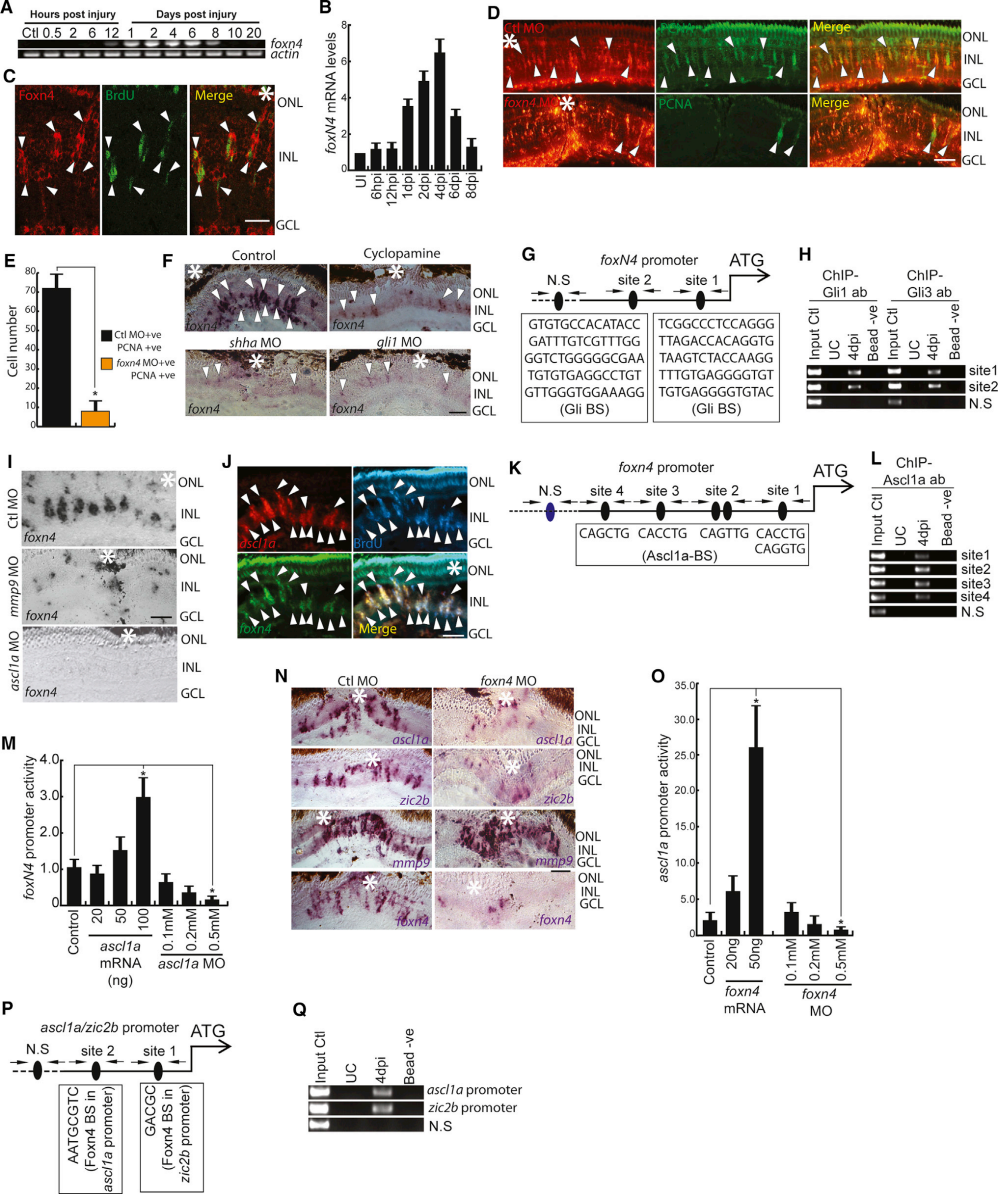
Expression Dynamics and Necessity of Foxn4 during Regeneration (A and B) RT-PCR (A) and qPCR (B) analysis of injury-dependent *foxn4* expression in the retina; n = 6 biological replicates. (C) IF microscopy of a 0.5-μm-thick optical section of retina revealing co-localization of Foxn4 with BrdU^+^ MGPCs in 4 dpi retina. (D and E) IF microscopy images of the retina with *foxn4* knockdown at 4 dpi (D). The number of PCNA^+^ MGPCs is quantified in (E). (F) BF microscopy images of *foxn4* mRNA ISH in retinal sections with cyclopamine treatment and *shha* or *gli1* knockdowns. (G and H) Schematic of *foxn4* promoter with a putative Gli-BS cluster, where arrows mark ChIP primers, N.S marks negative control, and capital letters mark putative Gli-BSs (G). A retinal ChIP assay at 4 dpi showing both Gli1 and Gli3 bound to the *foxn4* promoter (H). (I) BF microscopy images of *foxn4* mRNA ISH in retinal sections with *mmp9* or *ascl1a* knockdowns. (J) FISH and IF microscopy images of a 0.5-μm-thick optical section of retina showing co-localization of *foxn4* and *ascl1a* in BrdU^+^ MGPCs at 4 dpi. Arrowheads mark co-expression of genes in BrdU^+^ cells. (K and L) Schematic of the *foxn4* promoter with a putative Ascl1a-binding site cluster, where arrows mark ChIP primers, N.S marks negative control, and capital letters mark putative Ascl1a-BS (K). A retinal ChIP assay at 4 dpi showing Ascl1a bound to the *foxn4* promoter (L). (M) Luciferase assay showing *foxn4* promoter activity with overexpression or knockdown of *ascl1a* in 24 hpf embryos. (N) BF microscopy images of mRNA ISH in retinal sections with *foxn4* knockdown showing levels of genes (namely, *ascl1a*, *zic2b*, *mmp9*, and *foxn4*) at 4 dpi. (O) Luciferase assay showing *ascl1a* promoter activity with overexpression or knockdown of *foxn4* in 24 hpf embryos. (P and Q) Schematic of *ascl1a* and *zic2b* promoter with a putative Foxn4-binding site cluster, where arrows mark ChIP primers, N.S marks negative control, and capital letters mark putative Foxn4-BS (P). A retinal ChIP assay at 4 dpi showing Foxn4 bound to both the *ascl1a* and *zic2b* promoters (Q). Scale bars represent 10 μm (C, D, F, I, J, and N). Error bars represent SD. ^∗^p < 0.001 (M); ^∗^p < 0.04 (O). Biological replicates n = 6 in (M) and O, and n = 3 in (B). Asterisk marks injury spots in (C),(D),(F), (J) and (N). See also Figures S5–S7.

Foxn4, a member of the forkhead box family of proteins and discovered in retina microarray (Ramachandran et al., 2012) and RNA-seq analyses performed in the present study, showed an upregulation, with a peak expression at 4 dpi (Figures 6A and 6B). Foxn4 expression was restricted to BrdU^+^ MGPCs at 4 dpi (Figure 6C). Furthermore, we explored the significance of *foxn4* induction during retina regeneration. Interestingly, MO-mediated gene knockdown of *foxn4* inhibited MGPC induction up to 90% (Figures 6D, 6E, and S5A).

To ascertain whether *foxn4* is regulated through Shh signaling or its downstream effector genes, we adopted a pharmacological inhibition or gene-knockdown approach. Blockade of Shh signaling with cyclopamine or MOs against *shha* or *gli1* significantly abolished *foxn4* expression in the retina (Figures 6F, S4L, and S5B), whereas the opposite was seen with *sufu* knockdown (Figures S5C and S5D). Analysis of the *foxn4* promoter revealed 2 putative Gli-BS clusters (Figure 6G) that were strongly bound by Gli1 and Gli3, as revealed by a ChIP assay (Figures 6H and S2K), suggesting a direct involvement of Shh signaling in its expression. As discussed earlier, the influence of Mmp9 on expression levels of Shha led us to suspect its involvement in the regulation of *foxn4*. Knockdown of *mmp9* in 4 dpi retina caused a significant downregulation of *foxn4* (Figures 6I and S5E).

The temporal gene expression pattern and co-localization of *foxn4* with MGPCs prompted us to investigate its potential parallels with *ascl1a* gene. Fluorescence ISH (FISH) analysis showed co-expression of *ascl1a* and *foxn4* in BrdU^+^ MGPCs (Figure 6J). We then explored the possibility of a hierarchical regulation between *ascl1a* and *foxn4* during retina regeneration, as there is already a reported role for Foxn4 in the regulation of Ascl1 expression in mouse and chick (Del Barrio et al., 2007). We found significant downregulation of *foxn4* expression in retinal sections with knockdown of *ascl1a* (Figure 6I). *foxn4* promoter analysis predicted several Ascl1a-binding E-boxes (Bertrand et al., 2002, Li et al., 2006, Ramachandran et al., 2010a, Ramachandran et al., 2011), and binding was confirmed by a ChIP assay (Figures 6K, 6L, and S5G). The transactivation of the *foxn4* promoter by Ascl1a was confirmed with a luciferase assay, which was done by co-injection of *ascl1a* mRNA or MO against it, along with the promoter of *foxn4* driving the GFP-luciferase fusion construct in zebrafish embryos (Figure 6M). The mutation of Ascl1a-BS in the *foxn4* promoter had a negligible effect on its promoter activity both by *ascl1a* mRNA or by MO co-injections in zebrafish embryos (Figure S5H; Table S2).

**Figure 7:**
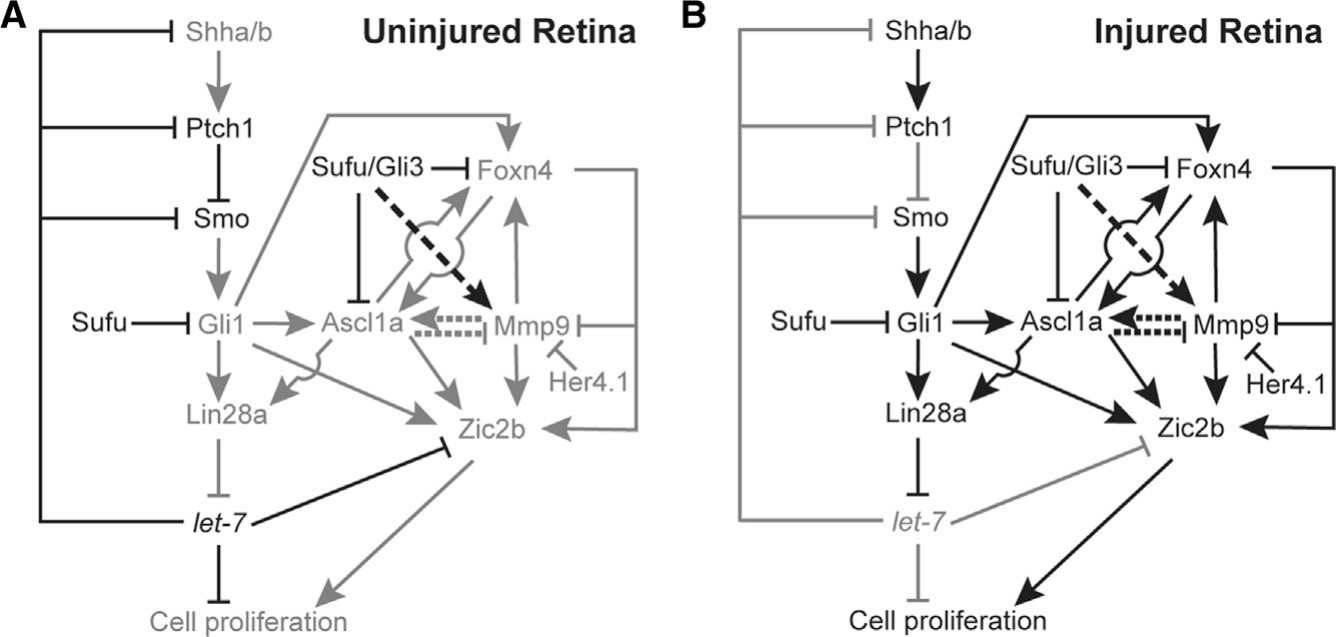
Schematic Representation of the Gene Regulatory Network during Retina Regeneration (A and B) Genetic interrelationships in uninjured (A) and injured (B) retina. Faded arrows and gene names show absence and bold shows presence. See also Figures S1–S7.

We then explored, using a knockdown approach in the retina, whether Foxn4 impacted *ascl1a* or other regeneration-associated genes such as *zic2b* and *mmp9*. We found that both *ascl1a* and *zic2b* were downregulated, which also explained the downregulation of *foxn4* itself, whereas no appreciable change was seen in *mmp9* levels (Figure 6N). A luciferase assay confirmed transactivation of the *ascl1a* promoter by Foxn4, which was done by co-injection of *foxn4* mRNA or MO against it, along with the promoter of *ascl1a* driving the GFP-luciferase fusion construct in zebrafish embryos (Figure 6O). Both the *ascl1a* and *zic2b* promoters harbor 2 potential Foxn4-binding sites (Luo et al., 2012) (Figure 6P), and this was confirmed by a ChIP assay, which was done using an antibody targeting Foxn4 (Figure 6Q). Mutated Foxn4-BS on the *ascl1a* promoter caused an almost complete alleviation of upregulated luciferase activity, as seen by its overexpression (Figures 6O and S5I; Table S2). These results suggest that *foxn4* expression is dependent on Shh signaling directly as well as through other genes such as *ascl1a*, which in turn regulates another regeneration-associated gene such as *zic2b* in a feedback loop. The findings from this study are summarized in a model (Figures 7A and 7B).

## Discussion

In this study, we explored the significance and potential regulators of Shh signaling during zebrafish retina regeneration. Our findings unravel mechanisms through which Shh signaling contributes to retina regeneration. We propose that Shh-dependent induction of Ascl1a and Lin28a contributes to Müller glia dedifferentiation through *let-7* microRNA-mediated translational downregulation of *shha*, *shhb*, *smo*, *ptch1*, and *zic2b* from respective mRNAs. Such stringent translational regulation probably accounts for the lack of an immature regenerative response despite the marginal expression of Shh signaling components such as *shha*, *shhb*, *smo*, and *ptch* in the uninjured retina. Cyclopamine-mediated repression of MGPCs might result from a decline in the regeneration-specific genes *ascl1a* and *lin28a*. This situation could be further exacerbated by upregulation of the repressor *insm1a* and the lack of the Delta-Notch signaling effector *her4.1*. These observations suggest the ability of Shh signaling to impinge upon various other signaling pathways important for regeneration.

Our results also show that Shh signaling impacted regeneration not only through transcription factors but also through negative regulation of enzymes such as Mmp9. Moreover, Mmp9-dependent expression of Shha causes the induction of Ascl1a as a prelude to MG dedifferentiation and MGPC induction. The increased expression of Mmp9 in a regeneration-compromised scenario like cyclopamine treatment (*shha* or *ascl1a* knockdown retina) suggests the existence of a feedback loop between Mmp9 and Shh signaling. The abundance of Mmp9 is probably due to the lack of Shha protein to give a feedback response for a decrease in its expression in MG to induce MGPCs. This observation is also supported by the *sufu* knockdown-mediated decline in *mmp9* expression. Co-labeling of *ascl1a* and *mmp9*, which was seen in a good number of cells, may appear paradoxical, but they all need not be Shh-positive or BrdU^+^. Only a subset of *ascl1a*-positive cells is *ptch1* positive and can have active Shh signaling and downregulated *mmp9*. The remainder of the *ascl1a* positive cells can have upregulated *mmp9* due to the lack of Shh signaling. Moreover, the Mmp9 expression is necessary for normal cycling of MGPCs during regeneration, and the repression of *mmp9* by Her4.1 could enable its expression restricted to the injury site at a later time. We anticipate a much wider role for the Shha-Mmp9-Ascl1a-Lin28a-*let-7* regulatory loop during retinal regeneration.

The induction of repressor Gli3 might cause the exit of MGPCs from the cell cycle to restrict the impact of a transcriptional activator, Gli1. This is evident from the knockdown results of *gli1* and *gli3* either in isolation or in combination. The *gli1* knockdown indicated a decline in the number of MGPCs, whereas *gli3* inhibition caused an expansion of MGPCs. Interestingly, double knockdown of *gli1* and *gli3* resulted in significant decline in MGPCs, suggesting that the Gli3 is necessary to quit the cell cycle as a prelude to differentiation. Similar results were seen with *zic2b* knockdown or cyclopamine treatment. This could be due to the impact of Shh signaling on the expression of downstream genes through Zic2b, although both Gli and Zic2b may compete or collaborate with the same binding sites on DNA. As *zic2b* mRNA shows a translational regulation through *let-7* microRNA, one could speculate that the role of Zic2b protein is restricted to Ascl1a- or Lin28a-expressing MGPCs.

The forkhead box gene family member *foxn4* is unique in its expression pattern during zebrafish development, with multiple isoforms in the thymus, skin, and brain (Danilova et al., 2004). We show the brain-specific isoform of *foxn4* is rapidly induced by Shh signaling, which orchestrates a series of gene expression events in response to retinal injury. Gli-BSs on the *foxn4* promoter is functional and probably explains the lack of its expression in the cyclopamine-treated retina. The regeneration-associated transcription factor Ascl1a significantly contributes to the induction of *foxn4*, suggesting dual control of its expression. Moreover, Foxn4 deficiency caused a significant reduction in MGPC number, probably through its effect on other regeneration-associated genes, which form a regulatory loop. To support this view, the proof that FoxN4 binds to promoters of *ascl1a* and *zic2b* at its consensus-binding sites (obtained from ChIP) makes it one of the central pillars of regeneration.

Taken together, our study sheds light on the mechanisms of MGPC induction in zebrafish retina in response to injury in an Shh-signaling-dependent manner and the significance of its downstream effector genes such as *ascl1a*, *lin28a*, *zic2b*, *foxn4*, and *mmp9*. These findings also suggest ways to coax mammalian MG dedifferentiation that may enable us to find ample solutions to intervene therapeutically for an efficient regenerative response.

## Experimental Procedures

Further details and an outline of resources used in this work can be found in Supplemental Experimental Procedures.

### Animals and Retinal Injury

Zebrafish were maintained at 26–28°C on a 14 hr/10 hr light/dark cycle for all experiments unless otherwise specified. The retinal injury was performed using a 30G needle as described previously (Fausett and Goldman, 2006). The C57BL/6 mice used in this study were maintained on a 12 hr/12 hr light/dark cycle with continuous access to food and water.

### RNA-Seq Analysis

The RNA-seq analysis of the total RNA of the retina at different time points post-injury and with cyclopamine treatment was performed as described previously (Brooks et al., 2012).

### Statistical Analysis

Observed data were analyzed for statistical significance by comparisons done using a two-tailed unpaired Student’s t test to analyze data from all experiments. Error bars represent SD in all histograms.

## Data and Software Availability

The accession number for the RNA-seq data reported in this paper is GEO: GSE102063.

## Supplemental Information

Document S1. Figures S1–S7 and Tables S1, S2, and S4

Table S3. List of Transcription Factors Regulated in Shh-Signaling-Inhibited Retina, Related to Figures 1 and S1 The table shows a list of transcription factors, transcription co-factors and chromatin remodeling factors along with their ENSEMBL IDs, obtained from RNA-seq data that are regulated during various time points, and Shh signaling inhibition with cyclopamine, post-retinal injury.

Table S5. List of Primers Used in the Study, Related to Figures 1, 2, 3, 4, 5, 6, and S1–S6 All the primers used in this study are listed with their code names indicating the genes in the first column and the 5′ to 3′ sequence, in the next column.

Document S2. Article plus Supplemental Information
