# Supplementary material for: let-7 MicroRNA-Mediated Regulation of Shh Signaling and the Gene Regulatory Network Is Essential for Retina Regeneration
Source: Cell Rep. 2018 May 2;23(5):1409–23. doi: 10.1016/j.celrep.2018.04.002 (PMC5946716; doi:10.1016/j.celrep.2018.04.002)
Supplement: Document S1. Figures S1–S7 and Tables S1, S2, and S4 [file mmc1.pdf]

Cell Reports, Volume *23*

Supplemental Information

let-7 MicroRNA-Mediated Regulation of Shh Signaling and the Gene Regulatory Network Is Essential for Retina Regeneration

Simran Kaur, Shivangi Gupta, Mansi Chaudhary, Mohammad Anwar Khursheed, Soumitra Mitra, Akshai Janardhana Kurup, and Rajesh Ramachandran

Experimental Procedures

Animals, fin cut, retinal injury and drugs.

Zebrafish were maintained at 26-28 °C on a 14 h:10 h light/dark cycle for all experiments unless specified. The *1016 tuba1a:*GFP transgenic fish used in this study have been characterized previously(Fausett and Goldman, 2006). Tricaine methanesulfonate is used as anesthetic. Fish embryos for all assays were obtained by natural breeding in laboratory. The Shh signaling inhibitor, cyclopamine; Mmp blockers, Salvianolic acid B and SB-3CT; protein transport inhibitor, Brefeldin A; Notch signaling blocker, *N*-[*N*-(3,5-difluorophenylacetyl)-L-alanyl]- *S*-phenylglycine *t*-butyl ester (DAPT), were made to a stock of 1mM, in DMSO for all experiments (all drugs were from Sigma-Aldrich). Drugs were delivered either through dipping or administration to the eye using a Hamilton syringe equipped with a 30-gauge needle. Retinal injury performed were previously described(Fausett and Goldman, 2006). All experiments were done to a minimum of six times for consistency and s.d.

C57BL /6 mice were used in this study. They were maintained at a cycle of 12 h light and 12 h dark cycle with continuous food and water accessibility. Animals were anaesthetized using isoflurane and eyes were injured or injected with a 30-gauge needle. Before harvesting the eyes animals were exposed to CO2 for euthanasia. The animal ethical committee at IISER Mohali approved these experiments.

RNAseq and analysis.

RNA was obtained from total retina from uninjured (control), 12 hours post injury and 4dpi, as previously described(Ramachandran et al., 2011), with or without cyclopamine treatment. The RNAseq was performed as shown previously(Brooks et al., 2012). The post sequence analysis were performed using TopHat and Cufflinks as reported earlier(Trapnell et al., 2012). The Supplementary Table S1 was created using a code developed in Python and the RNAseq data with special reference to transcription factors, obtained from the database AnimalTFDB2.0(Zhang et al., 2015), were analysed using it. The Venn diagrams in Figures S1J and S1K are created using FunRich (Functional Enrichment Analysis Tool; version 3.0) software(Pathan et al., 2015). The RNAseq data is deposited in repository at GEO Submission with ID of GSE102063.

Primers and plasmid construction

All primers are listed in Supplementary Table S5. The promoter of *her4.1* was amplified from zebrafish genomic DNA using primer pairs Xho-*her4.1*pro-F and Bam-*her4.1* pro-R (~4 kb). The digested PCR amplicon was cloned into a pEL luciferase expression vector to create *her4.1:*GFP-*luciferase* constructs. The *ascl1a:*GFP-*luciferase*, *lin28a:*GFP-*luciferase*, *insm1a:*GFP-*luciferase* constructs were described previously(Ramachandran et al., 2010a; Ramachandran et al., 2012).

Genes like *ascl1a*, *insm1a*, *lin28a*, and *nicd* were cloned from complementary DNA amplified from zebrafish retina RNA at 4 dpi using primer pairs Bam-Ascl1a FL-F and Xho-Ascl1a FL-R (~0.6 kb); Bam-*insm1a*-F and Xho-*insm1a*-R (~1.1kb); Bam-*lin28a* FL-F and Xho-*lin28a* FL-R (~0.6 kb). Post-digested PCR amplicons were cloned into their respective enzyme sites in pCS2$^+$ plasmid to obtain *cmv:ascl1a*, *cmv:insm1a* and *cmv:lin28a.* The *nicd* mRNA was prepared from PCR amplification using primer pairs T7-HSP M-F and Sv40-R (~2kb) from a clone of *nicd* driven by Hsp70 promoter, which in turn was made in pTAL plasmid vector by digesting an amplicon of *nicd* obtained using PCR primers Hind2X-flag-*nicd*-F and MluI NICD-R.
Micro-RNA response elements (MRE) sequences of *shhb*, *ptch1* and *smo* were cloned in pEGFP-C1 vector using BamHI and MfeI restriction sites, and *shha* and *zic2b* were cloned using BamHI and XhoI restriction sites. Site directed mutagenesis of various constructs were performed as described previously(Ramachandran et al., 2012).

For the confirmation of MO activity, an adaptor having respective MO targeted region for *gli1*, *gli2a*, *gli3*, *mmp9* or *ptch1* was cloned in pEGFP-N1 in XhoI and BamHI restriction sites, and *ptch2* was cloned in XhoI and AgeI site, which append in-frame to GFP reporter. The plasmid with and without respective MOs was injected to observe the absence or presence of GFP fluorescence under fluorescence microscope.

Total RNA isolation, RT-PCR and qPCR analysis.

Total RNA was isolated from dark-adapted zebrafish retinae of control, injured and drug treated/MO electroporated group using TRIzol (Invitrogen). A combination of oligo-dT and random hexamers were used to reverse transcribe approximately 5 μg of RNA using Superscript II reverse transcriptase

(Invitrogen) to generate cDNA. PCR reactions used Taq or Phusion (New England Biolabs) thermo polymerase and gene-specific primers (Supplementary Table S5) with previously described cycling conditions(Ramachandran et al., 2010a). Quantitative PCR (qPCR) was carried out in triplicate with KOD SYBR qPCR mix (Genetix, QKD-201) on a real-time PCR detection system (Eppendorf MasterCycler RealPlex4). The relative expression of mRNAs in control and injured retinae was deciphered using the ΔΔCt method and normalized to ribosomal protein *l*-24 or β-actin mRNA levels.

mRNA synthesis, embryo micro-injection and ChIP assay.
Gene clone of *nicd* cDNA in $pCS2^+$ plasmid was linearized and capped mRNAs were synthesized using the mMESSAGE mMACHINE (Ambion) *in vitro* transcription system. For luciferase assay experiments, single-cell zebrafish embryos were injected with a total volume of ~1nl solution containing 0.02 pg of *Renilla* luciferase mRNA (normalization), 5 pg of *promoter:*GFP-*luciferase* vector and 0-6 pg of *nicd* mRNA or 0.1 to 0.5mM *shha*/*sufu* MOs. To assure consistency of results, a master mix was made for daily injections and ~300 embryos were injected at single cell stage. 24 hours later, embryos were divided into 3 groups (~ 70 embryos/group) and lysed for dual luciferase reporter assays (Promega, catalogue number E1910).

Chromatin immunoprecipitation (ChIP) assays were done in adult retina at 4dpi using ~50 adult retinae after dark adaptation. Chromatin was isolated by sonication as described previously(Lindeman et al., 2009). The chromatin after sonication was distributed into three equal aliquots; two were probed with an anti-zebrafish Gli1, Gli3, Shha and Ascl1a antibodies (described below) and the third served as a control. Primers used for ChIP assays are described in Supplementary Table S5.

Morpholino (MO) electroporation.
MOs tagged with lissamine (Gene Tools) of approximately 0.5 µl (0.5 to 1.0 mM) were injected at the time of injury using a Hamilton syringe of 2 µl volume capacity. MO delivery to cells was accomplished by electroporation as previously described (Fausett et al., 2008). The control and *ascl1a* targeting MOs have been previously described (Ramachandran et al., 2012). Morpholinos targeting *shha*, *shhb*, *sufu*, *gli1*, *gli2a*, *gli3*, *patched1*, *patched2*, *mmp9* and *zic2b* are: *shha*(1)-5'-GCACTCTCGTCAAAAGCCGCATTTT-3'; *shha*(2)-5'-CACGCTGAAT CTCGCTGCGGTGTTC-3'; *shhb*-5'-TCAGATGCAGCCTTACGTCCATGAC-3'; *patched1*-5'-AGGAGACATTAACAGCCGAGGCCAT-3';*patched2*-5'-CCGGGTCT CTGGGATCCGAGGCCAT-3'; *gli1*-5'-CTCCATGATGAGACTTCTTGGATGA-3'; *gli2a*-5'-GGGTTCCATGACAACTGGGCATTCC-3'; *gli3*-5'-GTTCCATGACA ACTGGGCATTCCTC-3'; *sufu*-5'-ACGCCAGGACTCCAAGTCTCATTTT-3'; *mmp9*-5'-GCTGCATATCCACTGGCATCGAGAC-3'; *zic2b*(1)-5'-GGTGGCCGGC GTCCAGTAACATCAC-3'; *zic2b*(2)-5'-CACGATTATTGACCAAAGAATGCGT-3'

Cell culture and transfection and western blotting.
The HEK293T cells were grown in a 90 mm petriplate before seeding into 24-well plate at approximately 40% confluence and grown in Dulbecco's Modified Eagle Medium (DMEM), 10% (v/v) fetal bovine serum, with antibiotics and antimycotics in a 37 °C incubator with 5% CO2. Cells were then transfected after 24 h from time of plating. To examine involvement of *let-7* microRNA in regulation of gene expression, cells were transfected with 50 ng of pEGFP-C1 vector harboring GFP-reporter tagged to *shha*, *shhb*, *smo*, *ptch1* or *zic2b* cDNA, along with 0, 50, 200 or 500 ng of the *ubC:let-7a/let-7f* vector and 50 ng of the *β-actin2:mCherry* normalization vector. 48 h post-transfection, cells were harvested and protein expression was assayed by western blotting.
For *in vivo* experiments, Western blotting was performed using whole retina tissue using 4 retinae per experimental sample, lysed in Laemmli buffer, size fractioned in 12% acrylamide gel with SDS at denaturing conditions before transferring on to Immun-Blot PVDF membrane (Biorad Catalogue number 162-0177), followed by probing with specific primary antibodies and HRP conjugated secondary for chemiluminescence assay using Clarity Western ECL (Biorad Catalogue number 170-5061).

BrdU llabeling, Retina tissue preparation for mRNA *in situ* hybridization, immunofluorescence microscopy, and TUNEL Assay
BrdU labeling was performed by single i.p. injection of 20 µl of BrdU (20 mM) 3 h before euthanasia and retina dissection, unless mentioned specifically. Some animals required for long-term cell tracing experiments received more BrdU injections over multiple days. Fish were given higher dose of tricaine methane sulphonate and eyes were dissected, lens removed, fixed in 4% paraformaldehyde and

sectioned as described previously(Fausett and Goldman, 2006). The mRNA *in situ* hybridization (ISH) was performed on retinal sections with fluorescein or digoxigenin-labelled complementary RNA probes (FL/DIG RNA llabeling kit, Roche Diagnostics)(Barthel and Raymond, 2000) The micro RNA *let-7* ISH were done as described previously (Ramachandran et al., 2010a). Fluorescence ISH was performed according to the manufacturer's directions (Thermo Fisher Scientific, catalogue numbers T20917, B40955, B40953). Sense probes were used in every ISH separately as control, to assess the potential of background signal. Immunofluorescence microscopy protocols and antibodies were previously described(Ramachandran et al., 2010b; Ramachandran et al., 2012). Immunofluorescence microscopy was performed rabbit polyclonal antibody against human ASCL1/MASH1 (Abcam, catalogue number ab74065); Rat monoclonal antibody against BrdU (Abcam, catalogue number ab6326); Mouse monoclonal antibody against human proliferating cell nuclear antigen, PCNA (Santa Cruz, catalogue number sc-25280); Rabbit polyclonal antibody against zebrafish Gli1 (Anaspec, catalogue number AS-55627); Rabbit polyclonal antibody against zebrafish Gli3 (Anaspec, catalogue number AS-55630); Rabbit polyclonal antibody against zebrafish Patched 1 (Anaspec, catalogue number AS-55641); Rabbit polyclonal antibody against zebrafish Shha (Anaspec, catalogue number AS-55574s); Rabbit polyclonal antibody against zebrafish Smo (Anaspec, catalogue number AS-55647); Mouse polyclonal antibody against GFP (Abcam, catalogue number ab-38689); Rabbit polyclonal antibody against zebrafish mCherry (Abcam, catalogue number ab-183628); Mouse monoclonal antibody against Actin (Santa Cruz, catalogue number sc-81178); Rabbit polyclonal antibody against mouse glutamine synthetase (Abcam, catalogue number ab93439); Mouse polyclonal antibody against HuD (Santa Cruz, catalogue number sc-48421); Goat polyclonal antibody against protein kinase C β1 (PKCβ1) (Santa Cruz, catalogue number sc-209-G) at 1:500 dilution. Before BrdU immunofluorescence microscopy, retinal sections were treated with 2 N HCl at 37 °C for 20 min, equilibrated with 100mM sodium borate (pH 8.5) for 10 min, twice and then processed using standard procedures(Senut et al., 2004). BrdU labelled MGPC lineage-tracing experiments were done in retinal sections from single eye sections of 8 μm thickness, distributed across five slides. Individual slide was first processed for immunofluorescence based detection of specific antigen or mRNA and then BrdU or PCNA staining was performed as mentioned above using respective antibodies(Powell et al., 2012; Ramachandran et al., 2012). The total number of $BrdU^+$ cells and the number of co-labelled $BrdU^+$ cells that also stained with a specific ISH probe and subsequent enzymatic reaction, were quantified on each slide. TUNEL assay was performed on retinal sections using In Situ Cell death Detection Fluorescein kit (Roche, Ref no:11684795910) as per manufacturer recommended protocol.

Fluorescence and confocal microscopy, cell counting and statistical analysis.
After the completion of staining experiments, the slides were examined with a Nikon N*i*-E fluorescence microscope equipped with fluorescence optics and Nikon A1 confocal imaging system. The $PCNA^+$ and $BrdU^+$ cells were counted by observation of their fluorescence in retinal sections, ISH^+ cells through bright field, visualized in the same microscope and quantified. Every sections of the stained retina were mounted, observed and analysed, and at least three retinae from separate fish were used. Observed data were analysed for statistical significance by comparisons done using a two-tailed unpaired Student's *t*-test to analyse data from all experiments. For all other comparisons, analysis of variance (ANOVA) was performed and subsequently a Bonferroni/Dunn *post hoc t*-test was done using Stat View software. Error bars represent s.d in all histograms.

Fluorescence based cell sorting.
RNA was obtained from FACS purified MG and MG-derived progenitors at 4 dpi as previously described (Ramachandran et al., 2011, 2012). Briefly uninjured and injured retinas were isolated from *1016 tuba1a:*GFP transgenic fish. GFP+ MGPCs from *1016 tuba1a:*GFP retinas at 4 dpi were isolated by treating retinas with hyaluronidase and trypsin and then sorted on a BD FACS Aria Fusion high speed cell sorter. Approximately 40 injured retinas from *1016 tuba1a:*GFP fish yielded 80,000 GFP positive and 170,000 GFP cells.

References
Barthel, L.K., and Raymond, P.A. (2000). In situ hybridization studies of retinal neurons. Methods Enzymol *316*, 579-590.
Brooks, M.J., Rajasimha, H.K., and Swaroop, A. (2012). Retinal transcriptome profiling by directional next-generation sequencing using 100 ng of total RNA. Methods Mol Biol *884*, 319-334.

Fausett, B.V., and Goldman, D. (2006). A role for alpha1 tubulin-expressing Muller glia in regeneration of the injured zebrafish retina. J Neurosci *26*, 6303-6313.
Fausett, B.V., Gumerson, J.D., and Goldman, D. (2008). The proneural basic helix-loop-helix gene ascl1a is required for retina regeneration. J Neurosci *28*, 1109-1117.
Lindeman, L.C., Vogt-Kielland, L.T., Alestrom, P., and Collas, P. (2009). Fish'n ChIPs: chromatin immunoprecipitation in the zebrafish embryo. Methods Mol Biol *567*, 75-86.
Pathan, M., Keerthikumar, S., Ang, C.S., Gangoda, L., Quek, C.Y., Williamson, N.A., Mouradov, D., Sieber, O.M., Simpson, R.J., Salim, A.*, et al.* (2015). FunRich: An open access standalone functional enrichment and interaction network analysis tool. Proteomics *15*, 2597-2601.
Powell, C., Elsaeidi, F., and Goldman, D. (2012). Injury-dependent Muller glia and ganglion cell reprogramming during tissue regeneration requires Apobec2a and Apobec2b. J Neurosci *32*, 1096-1109.
Ramachandran, R., Fausett, B.V., and Goldman, D. (2010a). Ascl1a regulates Muller glia dedifferentiation and retinal regeneration through a Lin-28-dependent, let-7 microRNA signalling pathway. Nat Cell Biol *12*, 1101-1107.
Ramachandran, R., Reifler, A., Parent, J.M., and Goldman, D. (2010b). Conditional gene expression and lineage tracing of tuba1a expressing cells during zebrafish development and retina regeneration. J Comp Neurol *518*, 4196-4212.
Ramachandran, R., Zhao, X.F., and Goldman, D. (2011). Ascl1a/Dkk/beta-catenin signaling pathway is necessary and glycogen synthase kinase-3beta inhibition is sufficient for zebrafish retina regeneration. Proc Natl Acad Sci U S A *108*, 15858-15863.
Ramachandran, R., Zhao, X.F., and Goldman, D. (2012). Insm1a-mediated gene repression is essential for the formation and differentiation of Muller glia-derived progenitors in the injured retina. Nat Cell Biol *14*, 1013-1023.
Senut, M.C., Gulati-Leekha, A., and Goldman, D. (2004). An element in the alpha1-tubulin promoter is necessary for retinal expression during optic nerve regeneration but not after eye injury in the adult zebrafish. J Neurosci *24*, 7663-7673.
Trapnell, C., Roberts, A., Goff, L., Pertea, G., Kim, D., Kelley, D.R., Pimentel, H., Salzberg, S.L., Rinn, J.L., and Pachter, L. (2012). Differential gene and transcript expression analysis of RNA-seq experiments with TopHat and Cufflinks. Nat Protoc *7*, 562-578.
Zhang, H.M., Liu, T., Liu, C.J., Song, S., Zhang, X., Liu, W., Jia, H., Xue, Y., and Guo, A.Y. (2015). AnimalTFDB 2.0: a resource for expression, prediction and functional study of animal transcription factors. Nucleic Acids Res *43*, D76-81.

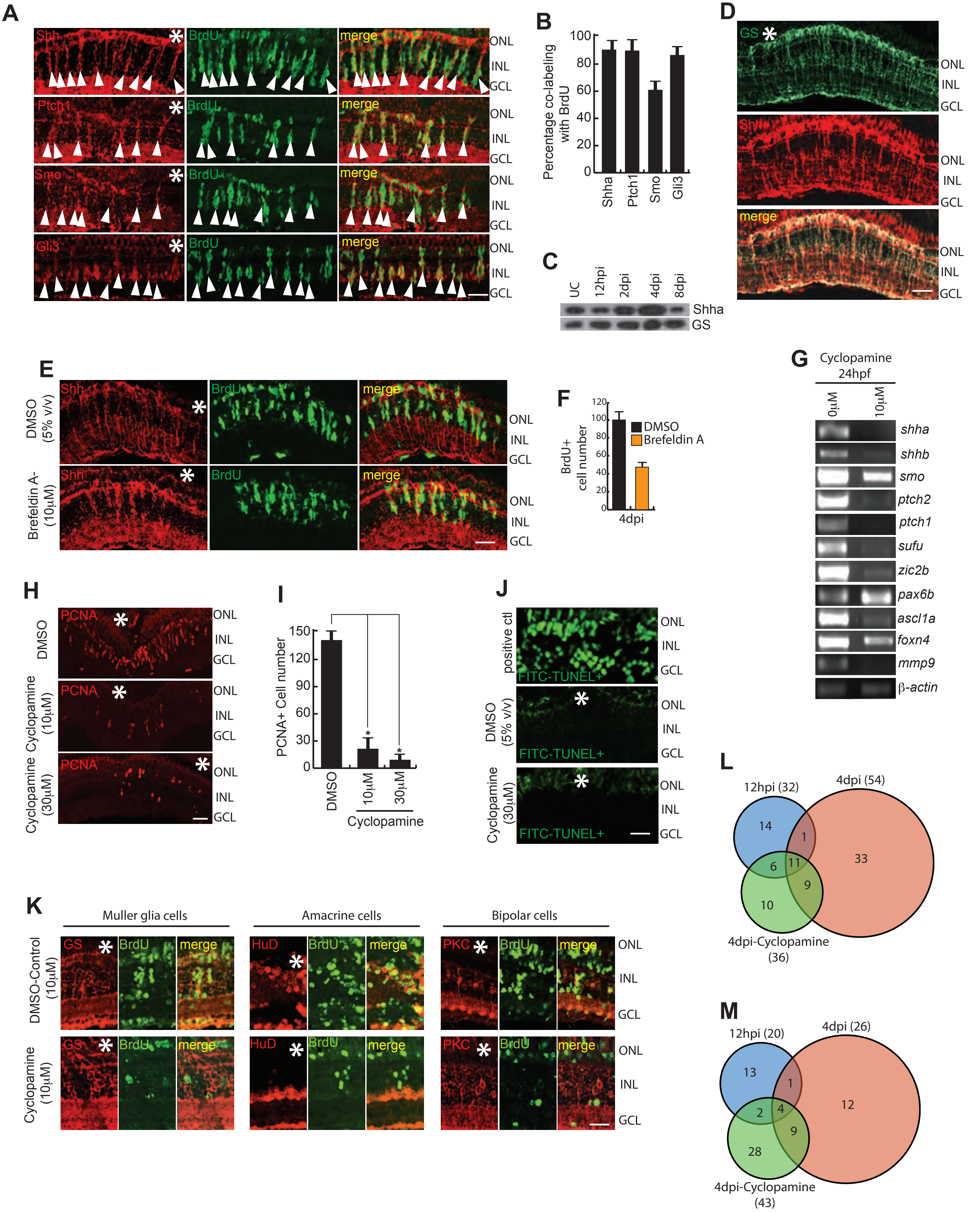

A
Shh
BrdU
merge
Ptch1
Smo
Gli3
ONL
INL
GCL
B
Percentage co-labeling with BrdU
100
80
60
40
20
0
Shha
Ptch1
Smo
Gli3
C
UC
12hpi
2dpi
4dpi
8dpi
Shha
GS
D
GS
Shh
merge
E
DMSO (5% v/v)
Brefeldin A- (10μM)
F
BrdU+ cell number
120
100
80
60
40
20
0
DMSO
Brefeldin A
4dpi
G
Cyclopamine 24hpf
0μM
10μM
shha
shhb
smo
ptch2
ptch1
sufu
zic2b
pax6b
ascl1a
foxn4
mmp9
β-actin
H
PCNA
DMSO
Cyclopamine (10μM)
Cyclopamine (30μM)
I
PCNA+ Cell number
150
120
90
60
30
0
DMSO
10μM
30μM
Cyclopamine
J
positive ctl
FITC-TUNEL+
DMSO (5% v/v)
Cyclopamine (30μM)
K
Muller glia cells
Amacrine cells
Bipolar cells
DMSO-Control (10μM)
Cyclopamine (10μM)
GS
HuD
PKC
L
12hpi (32)
4dpi (54)
14
1
6
11
33
9
10
4dpi-Cyclopamine (36)
M
12hpi (20)
4dpi (26)
13
1
2
4
12
9
28
4dpi-Cyclopamine (43)

Supplementary Figure S1. Shh signaling mediated gene expression and lineage tracing of MGPCs in cyclopamine treatment.

(**A,B**) High magnification immunofluorescence microscopy (IF) images of 4dpi retinal sections showed co-localization of Shh signaling components with $BrdU^+$ MGPCs (**A**)**,** which is quantified in (**B**). (**C**) Western blotting assay showed regulation of Shha protein following injury at various time points. (**D**) IF microscopy images of wild type 4dpi retinal sections revealed significant co-localization of Shha with Glutamine Synthetase (GS), which marks all the Muller glia, at the injury site. (**E**) IF microscopy of Shha in $BrdU^+$ MGPCs, in 4dpi retina, with Brefeldin A treatment, which is a protein transport inhibitor. (**F**) $BrdU^+$ cells were quantified in Brefeldin A treatment. (**G**) RT-PCR analysis of indicated genes mRNA levels in DMSO and cyclopamine treated 24hpf embryos. (**H,I**) IF microscopy images showed a cyclopamine dose-dependent decline in $PCNA^+$ MGPCs wild-type (**H**), retinae at 4 dpi, which is quantified in (**I**) (**J**) Terminal deoxynucleotidyl transferase (TdT) dUTP Nick-End Labeling (TUNEL) assay done on DNase treated positive control, 4dpi and cyclopamine treated 4dpi retinal sections, showed presence of $TUNEL^+$ cells only in positive control. (**K**) Cell-fate tracing experiment was done by injuring the fish followed by treating them with 5% (v/v) DMSO or 10μM cyclopamine for first four days followed by an i.p injection of BrdU and then were transferred to water for next 26 days until euthanasia. IF microscopy images of 30dpi retinal sections revealed co-localization of GS, which marks Muller glia, HuD which marks amacrine cells and PKC which marks horizontal cells, with $BrdU^+$ cells in DMSO treated retina but not in cyclopamine treated retina. (**L,M**) Whole retina RNAseq analysis of DNA binding proteins and transcription factors at 12hpi, 4dpi and 4dpi cyclopamine treatment were compared with uninjured retina showed upregulated genes (**L**), and downregulated genes (**M**). Scale bars, 10 μm (**A,E,H,J** and **K**) and 20 μm (**D**). Error bars are SD.

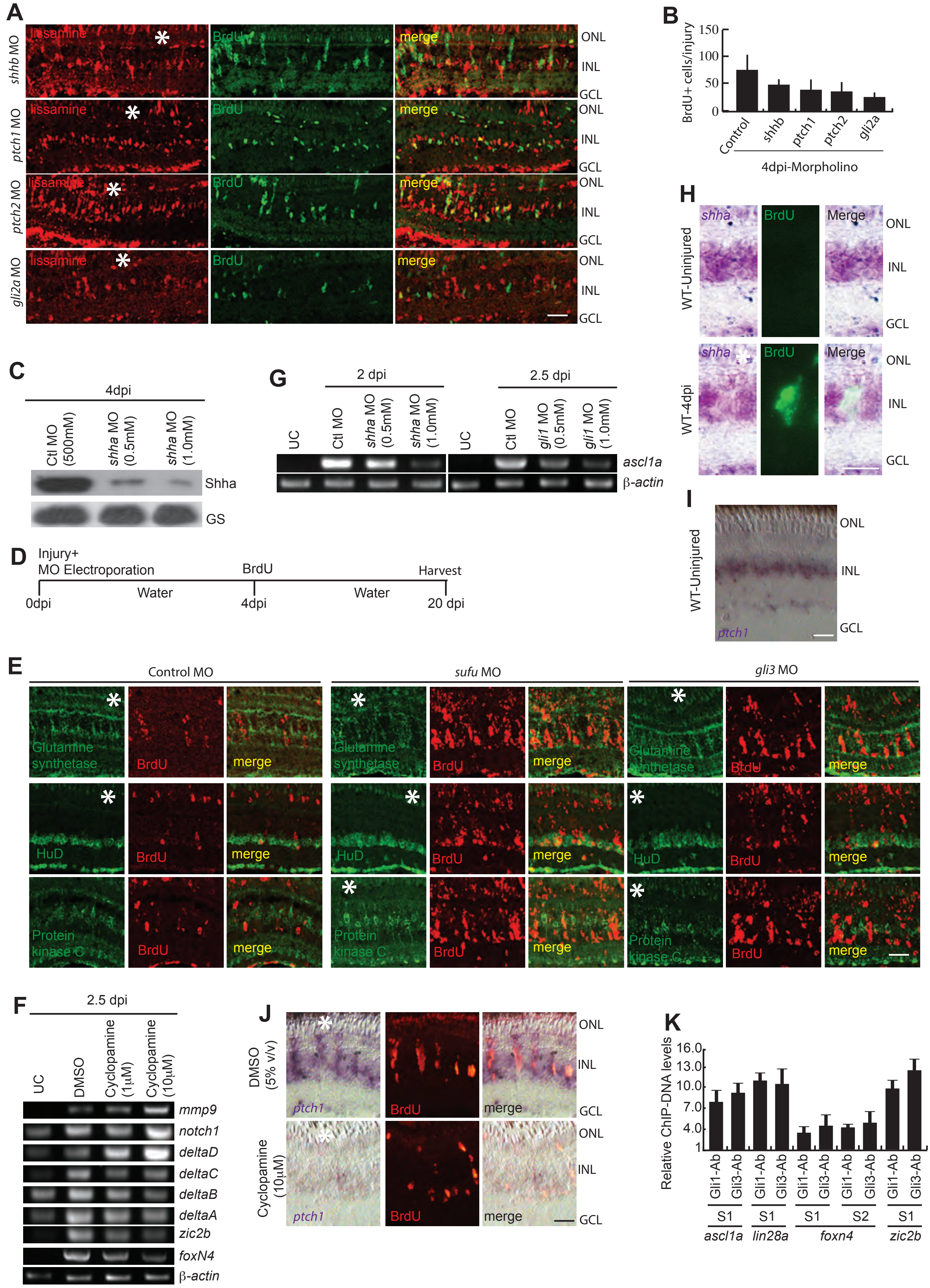
A
shhb MO
ptch1 MO
ptch2 MO
gli2a MO
lissamine
BrdU
merge
ONL
INL
GCL
B
BrdU+ cells/injury
150
100
50
0
Control
shhb
ptch1
ptch2
gli2a
4dpi-Morpholino
C
4dpi
Ctl MO (500mM)
shha MO (0.5mM)
shha MO (1.0mM)
Shha
GS
D
Injury+
MO Electroporation
BrdU
Harvest
Water
Water
0dpi
4dpi
20 dpi
E
Control MO
sufu MO
gli3 MO
Glutamine synthetase
HuD
Protein kinase C
BrdU
merge
F
2.5 dpi
UC
DMSO
Cyclopamine (1μM)
Cyclopamine (10μM)
mmp9
notch1
deltaD
deltaC
deltaB
deltaA
zic2b
foxN4
β-actin
G
2 dpi
2.5 dpi
UC
Ctl MO
shha MO (0.5mM)
shha MO (1.0mM)
gli1 MO (0.5mM)
gli1 MO (1.0mM)
ascl1a
β-actin
H
WT-Uninjured
WT-4dpi
shha
BrdU
Merge
I
WT-Uninjured
ptch1
J
DMSO (5% v/v)
Cyclopamine (10μM)
ptch1
BrdU
merge
K
Relative ChIP-DNA levels
16.0
13.0
10.0
7.0
4.0
1
Gli1-Ab
Gli3-Ab
S1
S2
ascl1a
lin28a
foxn4
zic2b

Supplementary Figure S2: Shh signaling component genes' knockdowns and lineage tracing of MGPCs in enhanced Shh signaling.

(**A,B**) IF microscopy images of 4dpi retina revealed decline in proliferation marked by reduction in $BrdU^+$ cells in *shhb*, *ptch1, ptch2* and *gli2a* knockdowns (**A**), which was quantified in (**B**). (**C**) Western blotting assay indicating *shha* knockdown caused downregulation in the expression of Shha protein. (**D,E**) The schematic of lineage tracing experiment, wherein control, *sufu* or *gli3* MOs were injected and electroporated while injuring the retina, and an i.p. injection of BrdU was given on 4dpi and eyes were harvested at 20dpi (**D**), the increased number of $BrdU^+$ cells could make retinal cell types (**E**). (**F**) RT-PCR analysis of indicated genes' mRNA levels in DMSO and cyclopamine treated 2.5dpi retina. (**G**) RT-PCR analysis of *ascl1a* mRNA levels in uninjured retina, control knockdown and *gli1* knockdown in 2.5dpi retina (**H**) Bright field (BF) and IF microscopy revealed the expression of *shha* mRNA and BrdU in uninjured and 4dpi retina. (**I,J**) ISH and IF of *ptch1* and BrdU respectively, in uninjured (**I**), 4dpi and 4dpi with cyclopamine treatment (**J**). (**K**) Relative abundance of ChIP DNA fragments obtained from Gli1 and Gli3 antibodies from various gene promoters, assayed by qPCR, which are normalized to control uninjured retina. Scale bars, 10 μm (**A,E,H,I,J**). Error bars are SD.

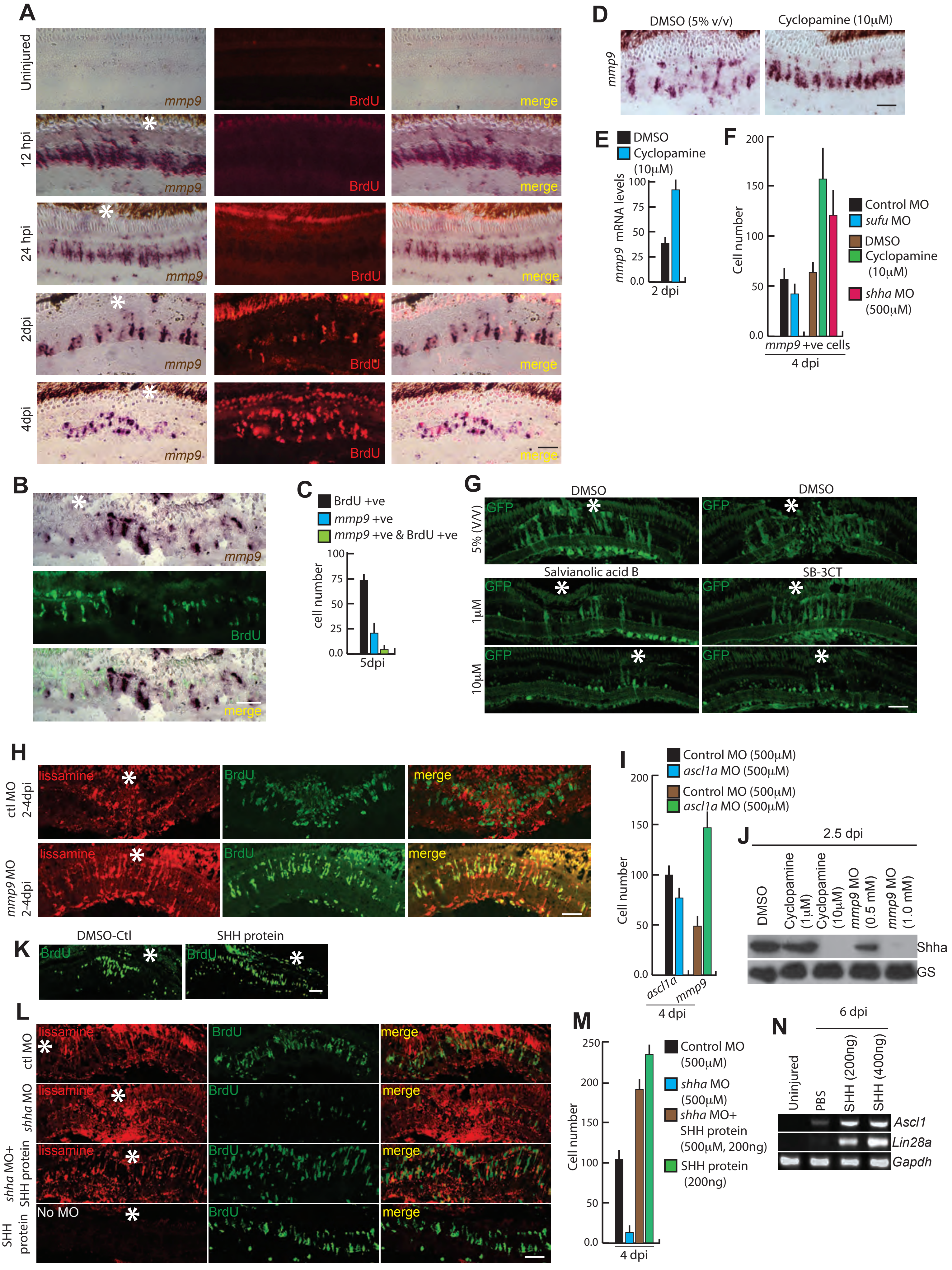

A
Uninjured
12 hpi
24 hpi
2dpi
4dpi
mmp9
BrdU
merge
B
C
BrdU +ve
mmp9 +ve
mmp9 +ve & BrdU +ve
cell number
5dpi
D
DMSO (5% v/v)
Cyclopamine (10μM)
E
DMSO
Cyclopamine (10μM)
mmp9 mRNA levels
2 dpi
F
Control MO
sufu MO
DMSO
Cyclopamine (10μM)
shha MO (500μM)
Cell number
mmp9 +ve cells
4 dpi
G
DMSO
Salvianolic acid B
SB-3CT
5% (V/V)
1μM
10μM
GFP
H
ctl MO 2-4dpi
mmp9 MO 2-4dpi
lissamine
I
Control MO (500μM)
ascl1a MO (500μM)
ascl1a
mmp9
J
2.5 dpi
DMSO
Cyclopamine (1μM)
Cyclopamine (10μM)
mmp9 MO (0.5 mM)
mmp9 MO (1.0 mM)
Shha
GS
K
DMSO-Ctl
SHH protein
L
ctl MO
shha MO
shha MO+ SHH protein
SHH protein
No MO
M
Control MO (500μM)
shha MO (500μM)
shha MO+ SHH protein (500μM, 200ng)
SHH protein (200ng)
N
6 dpi
Uninjured
PBS
SHH (200ng)
SHH (400ng)
Ascl1
Lin28a
Gapdh

Supplementary Figure S3: *mmp9* expression pattern and impact of SHH protein injection in regenerating retina.

(**A-C**) BF and IF microscopy images of *mmp9* and BrdU$^+$ cells at various time points post injury (**A**), and BrdU co-labeling with *mmp9* at 5dpi (**B**), quantified in (**C**). (**D,E**) ISH microscopy revealed increased *mmp9* expression in 4dpi retina with cyclopamine treatment (**D**), mRNA levels quantified in (**E**). (**F**) Quantification of *mmp9*$^+$ cells in 5% (v/v) DMSO control, 10µM cyclopamine treatment and *shha* or *sufu* knockdowns in 4dpi retina. (**G**) IF microscopy images show Salvianolic acid B and SB-3CT dependent decline in GFP$^+$ MGPCs in *1016 tuba:*GFP transgenic zebrafish at 4dpi. (**H**) MOs against control and *mmp9* were injected and electroporated at 2dpi, then an i.p. injection of BrdU was given on 4dpi, 3 hours before euthanasia, and no change in the number of BrdU$^+$ cells was found in both knockdowns. (**I**) Quantification of *ascl1a*$^+$ and *mmp9*$^+$ cells in control and *ascl1a* knockdown retina at 4dpi. (**J**) Western blotting assay indicating cyclopamine *or mmp9* knockdown in 2.5dpi retina caused decline in Shha expression levels. (**K**) Zone of BrdU$^+$ cells in the regenerating retina, increased upon injection of recombinant Shha protein (200ng) at 4dpi. (**L,M**) IF microscopy images revealed an increase in BrdU$^+$ cell number in combined injection of recombinant SHH protein and *shha* MO, and isolated injection of SHH protein (200ng) in 4dpi retina, whereas BrdU$^+$ cells declined in *shha* knockdown (**L**), which is quantified in (**M**), suggesting external SHH could impact retina regeneration even in absence of endogenous Shh protein. (**N**) RT-PCR analysis of *Ascl1* and *Lin28a* genes in 6dpi mouse retina exposed to recombinant SHH protein at the time of injury until harvest. Scale bars, 10 µm (**A,B,G,H,K,L**). Error bars are SD.

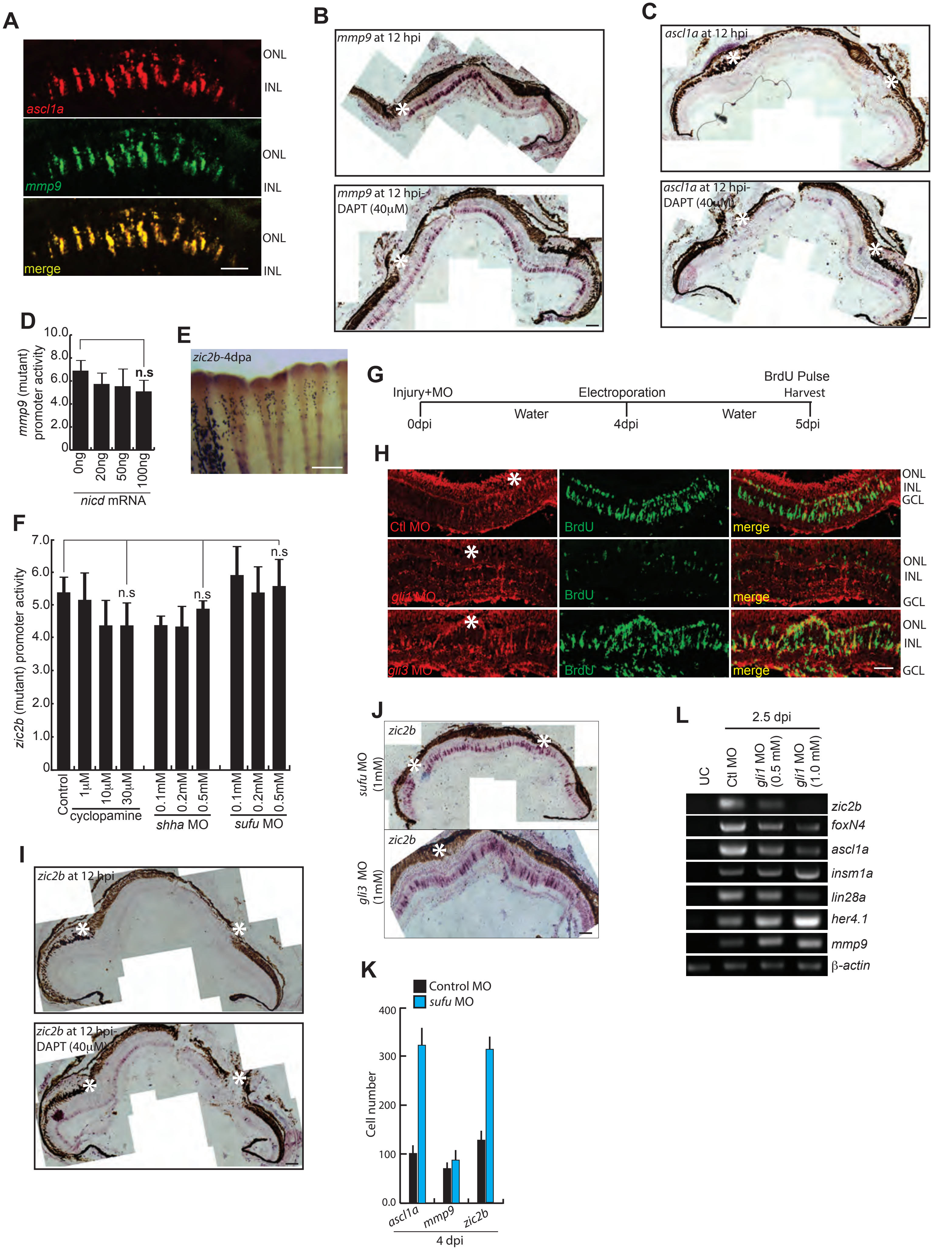
A
ascl1a
mmp9
merge
ONL
INL
B
mmp9 at 12 hpi
mmp9 at 12 hpi-DAPT (40μM)
C
ascl1a at 12 hpi
ascl1a at 12 hpi-DAPT (40μM)
D
mmp9 (mutant) promoter activity
10.0
8.0
6.0
4.0
2.0
0
n.s
0ng
20ng
50ng
100ng
nicd mRNA
E
zic2b-4dpa
F
zic2b (mutant) promoter activity
7.0
6.0
5.0
4.0
3.0
2.0
1.0
0
n.s
Control
1μM
10μM
30μM
cyclopamine
0.1mM
0.2mM
0.5mM
shha MO
sufu MO
G
Injury+MO
Electroporation
BrdU Pulse
Harvest
Water
0dpi
4dpi
5dpi
H
Ctl MO
gli1 MO
gli3 MO
BrdU
merge
GCL
I
zic2b at 12 hpi
zic2b at 12 hpi-DAPT (40μM)
J
zic2b
sufu MO (1mM)
gli3 MO (1mM)
K
Control MO
sufu MO
Cell number
400
300
200
100
0.0
ascl1a
mmp9
zic2b
4 dpi
L
2.5 dpi
UC
Ctl MO
gli1 MO (0.5 mM)
gli1 MO (1.0 mM)
foxN4
insm1a
lin28a
her4.1
β-actin

Supplementary Figure S4: Impact of DAPT treatment or *gli1*/*gli3* knockdowns in gene expression pattern and cell proliferation.

(**A**) FISH microscopy of *ascl1a* and *mmp9* in 4dpi retinal sections. (**B-C**) Low magnification BF microscopy images of mRNA *in situ* hybridization of *mmp9* (**B**) and *ascl1a* (**C**), in DAPT treated retina at 12hpi, showed an increase in its expression as compared with control. (**D**) Mutated Her/Hes binding sites abolished the impact of *nicd* over expression on *mmp9* promoter, in zebrafish embryo luciferase assay. (**E**) BF image of mRNA *in situ* hybridization of *zic2b* in 4 days post amputated zebrafish fin. (**F**) Luciferase assay showed that mutations to the Gli-BS abolished the impact of Shh signaling in *zic2b* promoter. (**G**) Schematic describing experimental regime of MO injection at the time of injury and electroporation at 4dpi, followed by an i.p. injection of BrdU at 5dpi before euthanasia. (**H**) IF microscopy images revealed decrease and an increase in $BrdU^+$ cells in *gli1 and gli3* knockdowns respectively. (**I**) Low magnification BF microscopy images of mRNA *in situ* hybridization of *zic2b* in DAPT treated retina, at 12hpi. (**J**) BF images of mRNA *in situ* hybridization of *zic2b* in *sufu* or *gli3* knockdown in 4dpi retina. (**K**) Quantification of *ascl1a*$^+$*, mmp9*$^+$ *and zic2b*$^+$ cells in control and *sufu* knockdown. (**L**) RT-PCR analysis of indicated genes' mRNA levels in uninjured retina, control and *gli1* knockdown retina in 2.5dpi. Scale bars, 10 µm (**A,B,C,H,I,J**) and 500 µm (**E**). Error bars are SD.

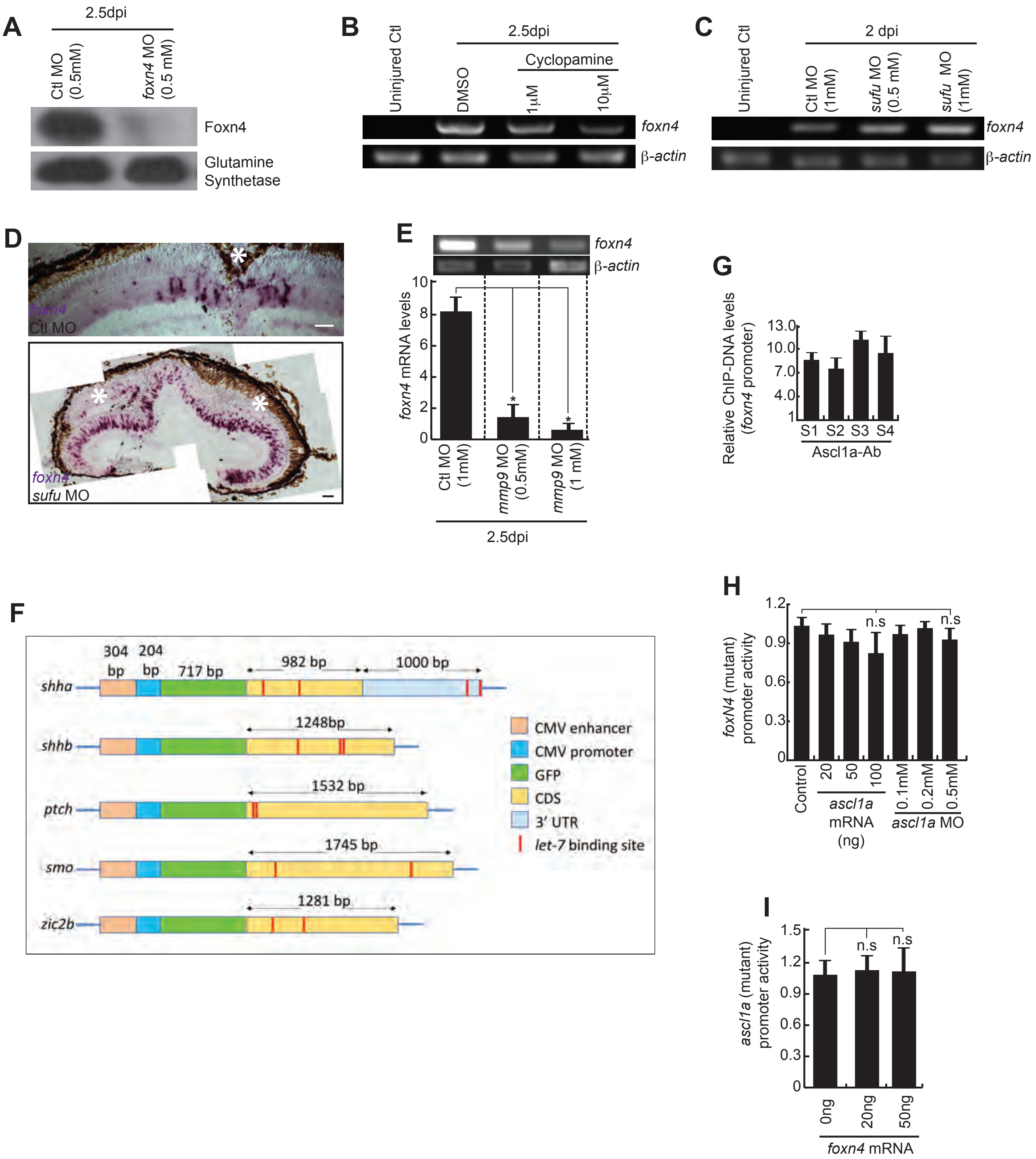
A
2.5dpi
Ctl MO (0.5mM)
foxn4 MO (0.5 mM)
Foxn4
Glutamine Synthetase
B
Uninjured Ctl
2.5dpi
DMSO
Cyclopamine
1μM
10μM
foxn4
β-actin
C
Uninjured Ctl
2 dpi
Ctl MO (1mM)
sufu MO (0.5 mM)
sufu MO (1mM)
foxn4
β-actin
D
Ctl MO
foxn4
sufu MO
E
foxn4
β-actin
foxn4 mRNA levels
Ctl MO (1mM)
mmp9 MO (0.5mM)
mmp9 MO (1 mM)
2.5dpi
G
Relative ChIP-DNA levels (foxn4 promoter)
S1 S2 S3 S4
Ascl1a-Ab
F
shha
shhb
ptch
smo
zic2b
304 bp
204 bp
717 bp
982 bp
1000 bp
1248bp
1532 bp
1745 bp
1281 bp
CMV enhancer
CMV promoter
GFP
CDS
3' UTR
let-7 binding site
H
foxN4 (mutant) promoter activity
n.s
Control
20
50
100
ascl1a mRNA (ng)
0.1mM
0.2mM
0.5mM
ascl1a MO
I
ascl1a (mutant) promoter activity
n.s
0ng
20ng
50ng
foxn4 mRNA

Supplementary Figure S5: Expression of *foxn4* in retina at various conditions

(**A**) Western blot analysis of Foxn4 in *foxn4*-MO electroporated retina, at 2.5dpi. (**B**) RT-PCR analysis of *foxn4* in uninjured control, 2.5dpi DMSO-treated, and 2.5dpi cyclopamine-treated whole retina. (**C**) RT-PCR analysis of *foxn4* from *sufu* MO-electroporated retina compared with control MO, at 2dpi. (**D**) BF microscopy images of *foxn4* mRNA ISH in retinal sections electroporated with control and *sufu* MOs at 4dpi. (**E**) RT-PCR (upper) and qPCR (lower) analysis of *foxn4* in control MO, and *mmp9* MO electroporated in 2.5dpi retina. $^{*}P<0.001$ in **E**, and error bars are SD. (**F**) Schematic representation of DNA constructs used in transfection experiments for examining the impact of *let-7* microRNA on various genes. (**G**)qPCR assay revealed the relative abundance of ChIP DNA fragments of *foxn4* promoter obtained by Ascl1a antibody which are normalized to control uninjured retina. (**H**) Luciferase assay revealed that mutated Ascl1a-BS on *foxn4* promoter had little effect on positive or negative regulation by *ascl1a* mRNA or MO respectively. (**I**) Luciferase assay revealed that mutated Foxn4-BS on *ascl1a* promoter had little effect on positive regulation by *foxn4* mRNA. Scale bars, 10 μm (**D**).

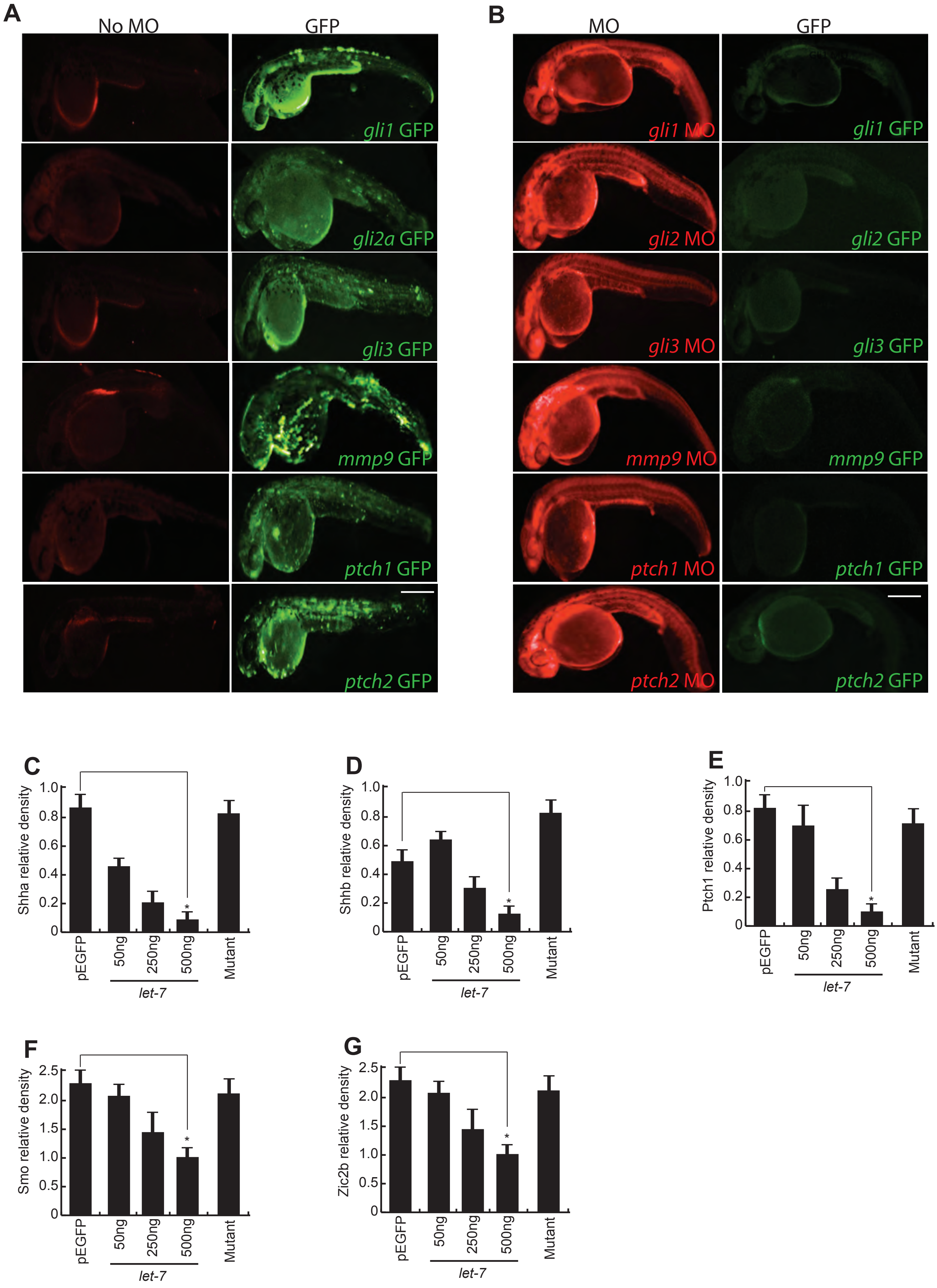

A
No MO
GFP
gli1 GFP
gli2a GFP
gli3 GFP
mmp9 GFP
ptch1 GFP
ptch2 GFP
B
MO
GFP
gli1 MO
gli1 GFP
gli2 MO
gli2 GFP
gli3 MO
gli3 GFP
mmp9 MO
mmp9 GFP
ptch1 MO
ptch1 GFP
ptch2 MO
ptch2 GFP
C
Shha relative density
D
Shhb relative density
E
Ptch1 relative density
F
Smo relative density
G
Zic2b relative density
pEGFP
50ng
250ng
500ng
Mutant
let-7

Supplementary Figure S6: MO assay in embryos. (**A,B**) The fusion mRNA, prepared by *in vitro* transcription using the clone containing GFP coding sequence in pCS2+ plasmid appended with the morpholino binding region of the respective genes, was injected alone (**A**), and along with morpholinos (**B**) in zebrafish embryos at single cell stage and imaged for GFP and lissamine fluorescence in a fluorescence microscope, at 24hpf. (**C-G**) Densitometry plots showing the expression of various GFP fusion proteins in *let-7* micro RNA dependent manner in HEK293T cells, normalized to transfection control mCherry. $^{*}P<0.0001$. Scale bars, 500 μm (**A,B**).

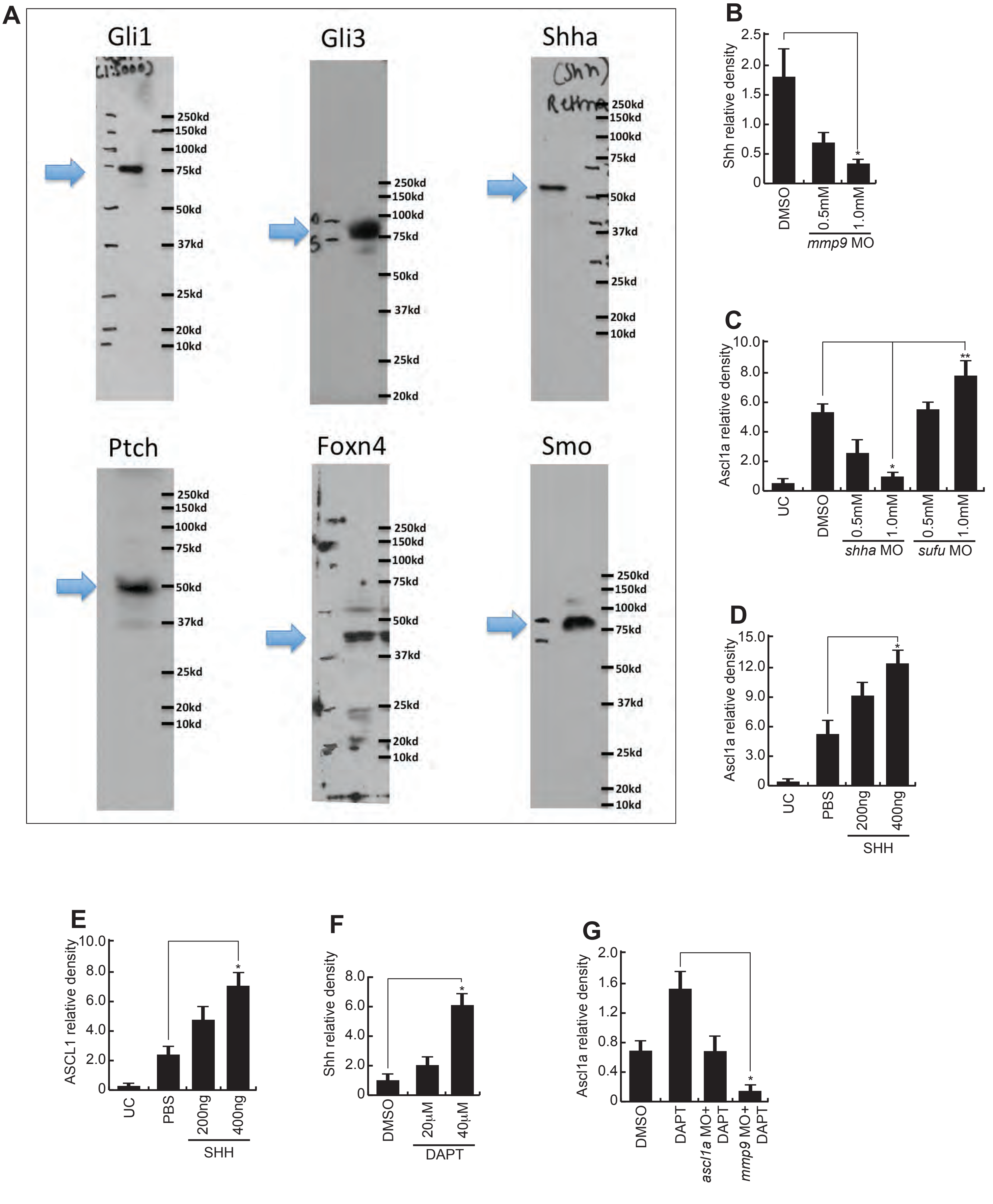
A
Gli1
Gli3
Shha
Ptch
Foxn4
Smo
250kd
150kd
100kd
75kd
50kd
37kd
25kd
20kd
10kd
B
Shh relative density
DMSO
0.5mM
1.0mM
mmp9 MO
C
Ascl1a relative density
UC
DMSO
0.5mM
1.0mM
0.5mM
1.0mM
shha MO
sufu MO
D
Ascl1a relative density
UC
PBS
200ng
400ng
SHH
E
ASCL1 relative density
UC
PBS
200ng
400ng
SHH
F
Shh relative density
DMSO
20μM
40μM
DAPT
G
Ascl1a relative density
DMSO
DAPT
ascl1a MO+ DAPT
mmp9 MO+ DAPT

Supplementary Figure S7: Western blotting of various proteins in retinal tissue. **(A)** The western blotting performed using total retina lysate revealed the unique expected bands (marked by arrows) of various proteins used in this study. (**B-G**) Densitometry plots showing the expression of various proteins in retina, normalized to control beta actin or glutamine synthetase. $^{*}P<0.0003$, $^{**}P<0.02$.

Lissamine tagged MO	# of injected fish	# of GFP + fish
gli1	63	1
gli2	72	0
gli3	61	1
mmp9	70	0
ptch1	67	0
ptch2	75	4

Supplemental Table 1. Statistical analysis of MO injection data. Related to Figure 1, Figure 2, Figure 3, Figure 4, Figure 5, and Figure S6. The MOs used in this study are validated in embryos co-injected with MO and the GFP construct with a prefixing of MO binding sequence in-frame with GFP.
The survival statistics is given in tables.

Gene	Wild type	Mutated
ascl1a (Mutated Gli BS)	GGGGCGTGGTCAGG	GGGGCGT**AAAA**A**A**G
	GGCGGGCCGCCGGCG	GGCG**AAAAAAAAAA**G
	CCGGAGCACCCCTG	CCGGA**AA**A**AAA**CTG
	TGAAGCCACACGTG	TGAA**AAA**A**A**ACGTG
	ACTGGGCAGTCCAA	ACT**AAAA**A**A**TCCAA
lin28 (Mutated Gli BS)	TTACACCACAGAAA	TTACA**AA**A**A**AGAAA
	GCAGTGTGATCGCT	GCAGTGT**A**AT**AAA**T
	GATGTGTGGTATTT	GA**TA**T**A**T**AA**TATTT
	TTTAGGAGGTGTGG	TTTAG**A**A**AA**T**A**TGG
	GATGTGTGGTATTT	GAT**A**T**A**T**AA**TATTT
zic2b (Mutated Gli BS)	ATACACGACGCACA	ATA**A**A**AA**ACGCACA
	AGAGACCCCAGAGA	AGAGA**AAA**AAGAGA
	GTGGGGTGCCCTGG	**A**T**A**GGGTG**AAA**TGG
	GGCGTGGGGTGCCC	**AAAA**T**A**GGGTG**AAA**
	CGTGAGGGGGCGTG	CGTGAGGG**AAAA**T**G**
foxn4 (Mutated Gli BS)	TTTGTGAGGGGTGT	TTTGT**A**A**AAAA**TGT
	TAAGTCTACCAAGG	TAAGT**AA**A**AA**AAGG
	TTAGACCACAGGTG	TTAGA**AA**A**A**A**AA**TG
	TCGGCCCTCCAGGG	T**AAAAAA**TCCAGGG
	TGTGAGGGGTGTAC	TGT**A**A**AAAA**TGTAC
foxn4 (Mutated Ascl1a BS)	CACCTG	**A**ACCTG
	CAGTTG	**A**AGTTG
ascl1a (Mutated Foxn4 BS)		
	ATAAGCGTAAA	**CCCC**GCGTAAA
mmp9 (mutated for Her4 BS)	CACAAG	**A**A**A**AAG
	CACAAG	**A**A**A**AAG
	CTGGTG	**A**T**AA**TG
	CTTGTG	CTT**AAA**

Micro RNA responsive elements

Gene	Wild type	Mutant
shha	GAGCTGTTGATATTACCACCTCT	GAGCTGTTGATATTA**A**CA**AAAA**T
	CACGACGCGACGTGTGTTTACGTCAT	CACGACGCGACGTGTGTT**AA**A**AAAAA**A**A**
	TGGCCATACCAGTTAACAAAAAATT	TGGCCATACCAGTTAACCTGCCTTT
	ATATTCAAACTGCTCCTTT	ATATTCAAACTG**AAAAAAA**
shhb	GGACGGGCAGTGGACATCACTACCTCAG	GGACGGGCAGTGGACATCACTA**AAAA**A**A**
	CACCAAGCTCACCCTCACTGCCGCGCAC	CACCAAGCTCACCCTC**AAAAAA**GCGCAC
	TGCCGCGCACCTAGTTTTCGTTGGAAACTCTTCAG	TGCCGCGCACCTAGTTTTCGTTGGAAACT**AAAA**AG
ptch1	GAATATGCACAGTTTCCCTTCTACCTCA	GAATATGCACAGTTTCCCTT**AA**A**AAAA**A
	GAGCCCATCGAATATGCACAGTTTCCCTTCTACCTCAA	GAGCCCATCGAATATGCACAGTTTCCCTT**AA**ACCTCAA

smo	CACTATGCGACTTGGAGAGCCATCA	CACTATGCGACTTGGAGA**AAA**A**AA**A
	AGTACGGCCAGCGGGTCCTGCAG	AGTACGGCCAGCGGGT**AAAA**A**A**
zic2b	CGGCGGCGCACGCTGCCTCT	CGGCGGCGCAC**AAAAAAAA**T
	GAGCGCATGCGGCGGCGCACGCTGCCTCT	GAGCGCATGCGGCGGCGCAC**AAAAAAAA**T

Supplemental Table 2. Wild type and mutated regions of various DNA constructs. Related to Figure 2, Figure 3, Figure 4, Figure 5, and Figure S6.The mutations, created on DNA sequences for disrupting transcription factor binding sites and let-7 micro RNA responsive elements of various constructs used in this study are highlighted in bold letters.

<table>
<tr><th>ZF Gene</th><th>Ensembl ID #</th><th>Position</th><th>Heteroduplex</th><th>let-7 miRNA</th></tr>
<tr><td rowspan="4">Shha</td><td rowspan="4">ENSDARG00000068567</td><td>750-772(CR)</td><td>GAGCUGUUGAUAUUACCACCUCU
 |||||| | |||| |||||
 UUGAUAUGUUGGAUGAUGGAGU</td><td>let-7a, b, c, d, e, f, g, h, i</td></tr>
<tr><td>1057-1083(CR)</td><td>CACGACGCGACGUGUGUUUUACGUCAU
 |||||||||| || |||| |||
UU GUUGUGUUGA AU GAUGGAGU</td><td>let-7h,i</td></tr>
<tr><td>2471-2494
(3'UTR)</td><td>UGGCCAUACCAGUUAACCUGCCUU
 |||||||| || || ||||||
 UUGGUAUG UUGGUU GAUGGAU</td><td>let-7d</td></tr>
<tr><td>2571-2589
(3'UTR)</td><td>AUAUUCAAACUGCU CCUUU
 |||||||| ||||| ||||
UUGAUAAGUUAGAUGAUGGAGU</td><td>let-7e</td></tr>
<tr><td rowspan="3">Shhb</td><td rowspan="3">ENSDARG00000038867</td><td>649-676(CR)</td><td>GGACGGG CAGUGGACAUC ACUACCTCAG
 ||| || ||| |||||||||
 UUGAUAAGU UAGAUGAUGGAGU</td><td>let-7e

let-7c, d</td></tr>
<tr><td>1005-1032(CR)</td><td>CACCAAGCUCACCCUCACUGCCGCGCAC
 |||| || ||| |||||| ||
UUGGUAUGUU GGA UGAUGGA GU</td><td></td></tr>
<tr><td>1023-1057(CR)</td><td>UGCCGCGC ACCUAGUUUUCGUUGGAAACU CUUCAG
 ||||||| |||| ||| |||||
UUGGUGUGUUGGA UGAUGGAGU</td><td>let-7b</td></tr>
<tr><td rowspan="2">Ptch1</td><td rowspan="2">ENSG00000185920</td><td>2590-2617(CR)</td><td>GAAUAUGCACAGUUUCCCUU CUACCUCA
 |||| |||||| ||| |||||||
 UUGU UGUGUU GAAUGAUGGAGU</td><td>Let-7h, i</td></tr>
<tr><td>2581-2618(CR)</td><td>GAGCCCAUCGAAUAUGCACAGUUUCCCUUCUACCUCAA
 |||| ||||| ||| ||||||||
 UUGG UAUGUU GGAUGAUGGAGU</td><td>Let-7b</td></tr>
<tr><td rowspan="2">Smo</td><td rowspan="2">ENSDARG00000002952</td><td>1472-1497(CR)</td><td>CACUAUGCGACUUGGAGAGCCAUCAU
 ||||||||||||| ||| |||
UUGAUAUGUUGGAUGA UGG AGU</td><td>let-7a, b, c, d, e, f, g, h, i</td></tr>
<tr><td>2612-2634(CR)</td><td>AGUACGGCCAGCGGGUCCUGCAG
 ||||||||||||| ||| ||
UUGGUAUGUUGGUUGAU GGA GU</td><td>let-7c, d</td></tr>
<tr><td rowspan="2">Zic2b</td><td rowspan="2">ENSDARG00000037178</td><td>500-519(CR)</td><td>CGGCGGCGCAC GCUGCCUCU
 ||||||||| ||||||||
 UUGUUGUGUUGAAUGAUGGAGU</td><td>let-7h

let-7c, d</td></tr>
<tr><td>491-519(CR)</td><td>GAGCGCAUGCGGCGGCGCACGCUGCCUCU
 ||| |||||||| ||||||||
 UUG GUAUGUUGGA UGAUGGAGU</td><td></td></tr>
</table>

Supplemental Table 4. List of genomic regions with let-7 micro RNA binding sites. Related to Figure 3 and Figure 5. The table shows a list of genomic regions of genes, mentioned in this study, with the micro RNA recognition elements (MREs) URL:https://bibiserv2.cebitec.unibielefeld.de/rnahybrid;jsessionid=be1041a93a76d436824f6e0f235b.
