# Supplementary material for: let-7 MicroRNA-Mediated Regulation of Shh Signaling and the Gene Regulatory Network Is Essential for Retina Regeneration
Source: Cell Rep. 2018 May 2;23(5):1409–23. doi: 10.1016/j.celrep.2018.04.002 (PMC5946716; doi:10.1016/j.celrep.2018.04.002)
Supplement: Document S2. Article plus Supplemental Information [file mmc4.pdf]

Cell Reports

Article

let-7 MicroRNA-Mediated Regulation of Shh Signaling and the Gene Regulatory Network Is Essential for Retina Regeneration

Graphical Abstract

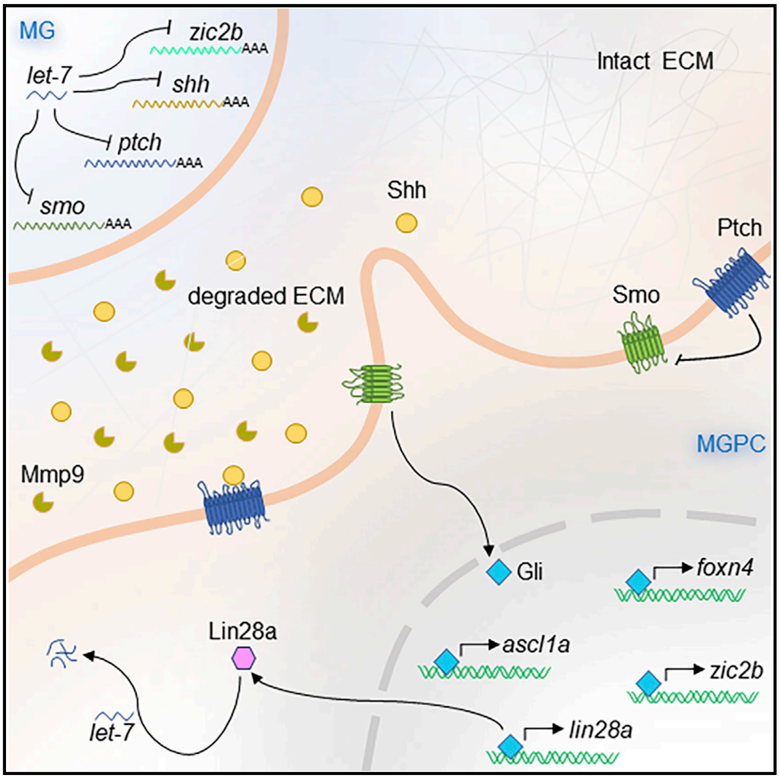

Highlights

- Shh signaling is essential for MG dedifferentiation during retina regeneration
- Shh signaling components are regulated by *let-7* microRNA in the zebrafish retina
- A regulatory feedback loop between Mmp9 and Shh signaling is active in the retina
- Shh signaling induced a gene-regulatory network involving *mmp9*, *ascl1a*, *zic2b*, and *foxn4*

Authors

Simran Kaur, Shivangi Gupta, Mansi Chaudhary, Mohammad Anwar Khursheed, Soumitra Mitra, Akshai Janardhana Kurup, Rajesh Ramachandran

Correspondence

rajeshra@iisermohali.ac.in

In Brief

Kaur et al. demonstrate that microRNA *let-7* in injured zebrafish retina regulates the translation of *shha*, *shhb*, *smo*, *ptch1*, and *zic2b* mRNAs. Further, Shh signaling is necessary during retina regeneration for inducing a pro-regenerative gene expression cascade involving several genes, including *ascl1a*, *lin28a*, *mmp9*, *foxn4*, and *zic2b*.

Data and Software Availability

GSE102063

Kaur et al., 2018, Cell Reports *23*, 1409–1423
May 1, 2018 © 2018 The Author(s).
https://doi.org/10.1016/j.celrep.2018.04.002

OPEN ACCESS
CellPress

let-7 MicroRNA-Mediated Regulation of Shh Signaling and the Gene Regulatory Network Is Essential for Retina Regeneration

Simran Kaur,[1] Shivangi Gupta,[1,2] Mansi Chaudhary,[1,2] Mohammad Anwar Khursheed,[1] Soumitra Mitra,[1] Akshai Janardhana Kurup,[1] and Rajesh Ramachandran[1,3,*]
[1]Department of Biological Sciences, Indian Institute of Science Education and Research, Mohali, Knowledge City, SAS Nagar, Sector 81, Manauli PO, 140306 Mohali, Punjab, India
[2]These authors contributed equally
[3]Lead Contact
*Correspondence: rajeshra@iisermohali.ac.in
https://doi.org/10.1016/j.celrep.2018.04.002

SUMMARY

Upon injury, Müller glia cells of the zebrafish retina reprogram themselves to progenitor cells with stem cell characteristics. This necessity for retina regeneration is often compromised in mammals. We explored the significance of developmentally inevitable Sonic hedgehog signaling and found its necessity in MG reprogramming during retina regeneration. We report on stringent translational regulation of *sonic hedgehog*, *smoothened*, and *patched1* by *let-7* microRNA, which is regulated by Lin28a, in Müller glia (MG)-derived progenitor cells (MGPCs). We also show Shh-signaling-mediated induction of Ascl1 in mouse and zebrafish retina. Moreover, Shh-signaling-dependent regulation of *matrix metalloproteinase9*, in turn, regulates Shha levels and genes essential for retina regeneration, such as *lin28a*, *zic2b*, and *foxn4*. These observations were further confirmed through whole-retina RNA-sequencing (RNA-seq) analysis. This mechanistic gene expression network could lead to a better understanding of retina regeneration and, consequently, aid in designing strategies for therapeutic intervention in human retinal diseases.

INTRODUCTION

In contrast to mammals, zebrafish retina possesses remarkable regenerative capacity after an acute injury, leading to functional restoration of vision (Sherpa et al., 2008). The Müller glia (MG) cells in zebrafish retina reprogram themselves to MG-derived progenitor cells (MGPCs) that systematically differentiate into all retinal neurons, namely rods, cones, horizontal, amacrine, ganglion, bipolar cells, and MG itself (Ramachandran et al., 2010b). Although induction of MGPCs immensely contributes to the successful regeneration of zebrafish retina, the complete mechanism remains elusive. While the mechanism of retina regeneration is histologically well described, only a subset of the involved genes/proteins has been identified and characterized functionally (Goldman, 2014; Wan and Goldman, 2016). Therefore, we attempted to identify previously uncharacterized regulators of zebrafish retina regeneration using the needle-poke method of injury, which reflects the situation of mechanical damage that occurs in nature.

Even though several studies have elucidated the importance of Delta-Notch, Wnt, and Fgf signaling during retina regeneration in zebrafish, the roles of developmentally important Shh signaling remain largely underexplored (Goldman, 2014; Sun et al., 2014; Wan and Goldman, 2016). Recent studies have revealed the potential roles of Shh signaling during tissue regeneration (Ando et al., 2017; Dunaeva and Waltenberger, 2017; Thomas et al., 2018; Todd and Fischer, 2015). Therefore, we investigated the mechanistic involvement of Shh signaling during zebrafish retina regeneration. Subsequently, we hypothesized that MG dedifferentiation may depend on Shh signaling and have some similarities to the reprogramming of somatic cells by pluripotency-inducing factors (Hochedlinger and Plath, 2009; van den Hurk et al., 2016). Since we were interested in the possible involvement of Shh signaling during the early regenerative response of MG to injury, we analyzed the retina within the first few days after blockade of Shh signaling. We identified expression pattern of several important genes induced by Shh signaling and vice versa that reveal the robust regulatory network associated with retina regeneration. These include the interplay of Shh/Notch signaling components, transcription factors (namely, Ascl1a, Zic2b, Foxn4, and Insm1a), the matrix metalloproteinase Mmp9, the RNA-binding protein Lin28a, and microRNA *let-7*. Complete retina regeneration in zebrafish has provided valuable clues as to why their mammalian counterparts often fail (Goldman, 2014; Wan and Goldman, 2016). The findings from this study add clarity to the enigmatic process of retina regeneration lacking in mammals.

RESULTS

Injury-Dependent Induction of Shh Signaling Is Essential for Regeneration

We explored the temporal expression pattern of Shh signaling component genes such as *sonic hedgehog* (*shha*, *shhb*), smoothened (*smo*), *patched1* (*ptch1*), *patched2* (*ptch2*), *dispatched1* (*disp1*), *dispatched2* (*disp2*), and *glioma-associated*

Check for updates

This is an open access article under the CC BY license (http://creativecommons.org/licenses/by/4.0/).

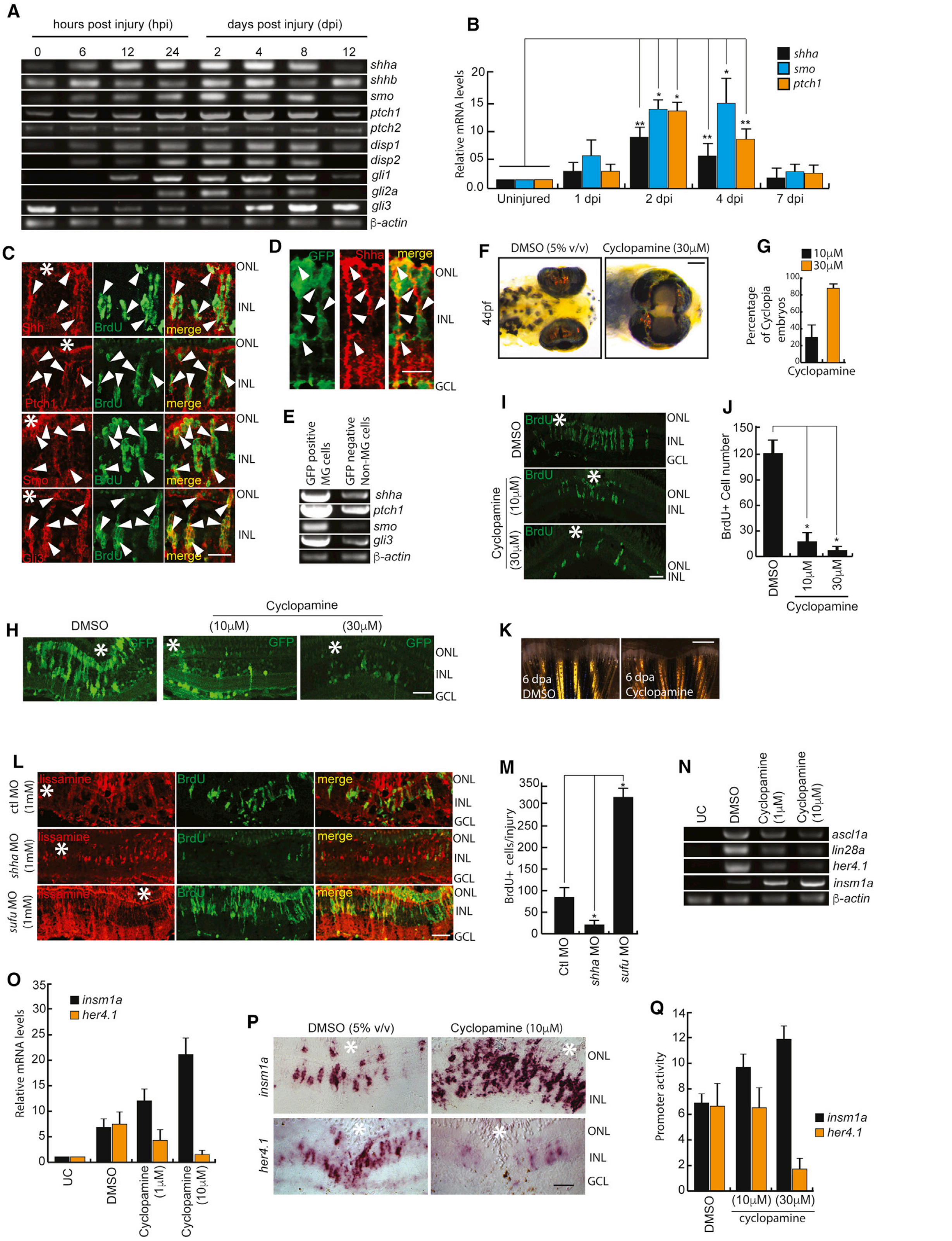

(legend on next page)

oncogene (*gli1*, *gli2a*, and *gli3*) in total retina. We found that most of these genes were upregulated after retinal injury, except *gli3*, which showed a downregulation (Figures 1A and 1B). Moreover, the Shh signaling components Shh, Ptch1, Smo, and Gli3 showed co-localization with bromodeoxyuridine (BrdU)$^+$ MGPCs (Figures 1C, S1A, S1B, and S7A). Western blot analysis revealed a temporal upregulation of Shh protein with a peak of expression at 4 days post-injury (dpi) (Figures S1C and S7A). The Shh protein is expressed in MG cells of wild-type (WT) injured retina marked by glutamine synthetase (GS) at 4 dpi (Figure S1D). Using *tuba1a1016*:GFP transgenic zebrafish (Fausett and Goldman, 2006), we showed the expression of Shh and its signaling components in proliferating MGPCs marked by GFP. Immunofluorescence (IF) studies and cell sorting revealed a relative abundance of Shh protein and its signaling components in GFP$^+$ MGPCs compared with the rest of the cells of *tuba1a1016*:GFP transgenic retina at 4 dpi (Figures 1D and 1E). We confirmed the secretion of Shha and its probable autocrine action in MG using brefeldin A, a protein transport inhibitor, (Miller et al., 1992) and observed an expected increase in intracellular Shha and a decline in BrdU$^+$ cells (Figures S1E and S1F).

To decipher the influence of Shh signaling on retina regeneration, we used the pharmacological agent cyclopamine (Incardona et al., 1998), a potent inhibitor of Smo (Chen et al., 2002). We found that at 30 μM concentration, 90% of zebrafish embryos exhibited cyclopia, a hallmark of impaired Shh signaling, which also impacted developmentally important genes (Figures 1F, 1G, and S1G). We then explored the impact of continuous cyclopamine exposure on MGPC induction and regeneration in WT and *tuba1a1016*:GFP transgenic retina at 4 dpi. Interestingly, 10 μM and 30 μM concentrations significantly inhibited MGPC induction (Figures 1H–1J, S1H, and S1I), which was not the result of enhanced apoptosis (Figure S1J). A similar reduction in fin blastema was also seen with cyclopamine treatment on the 6th day post-amputation (Figure 1K), suggesting a conserved Shh signaling mechanism across tissues during regeneration. The few residual BrdU$^+$ MGPCs in cyclopamine-treated retina failed to form any retinal cell types (Figure S1K). Moreover, morpholino (MO)-based targeted gene knockdown of Shh signaling component genes such as *shha*, *shhb*, *ptch1*, *ptch2*, and *gli2a* caused progenitor reduction, and that of negative regulators *sufu* (*suppressor of fused*) (Figures 1L and 1M and S2A–S2C) and *gli3* (Figures 5I, S6A, and S6B; Table S1) enhanced MGPC induction as compared with control retina at 4 dpi. These increased MGPCs when traced until 20 dpi revealed the formation of amacrine, bipolar, and MG cells, indicating their functional potential to give rise to different retinal cell types (Figures S2D and S2E). These results emphasize the importance of Shh signaling during retina regeneration.

We also performed whole-retina RNA sequencing (RNA-seq) at 12 hr post-injury (hpi), 4 dpi, and 4 dpi with cyclopamine treatment compared with uninjured controls to get a holistic view of the blockade of Shh signaling. We found that several transcription factor genes, including *ascl1a*, *zic2b*, *foxn4*, and matrix metalloproteinase *mmp9*, are regulated with cyclopamine treatment (Table S3; Figures S1L and S1M; GEO: GSE102063).

Shh Signaling Affects Expression of Repressor Genes

We then explored the impact of compromised Shh signaling in the expression pattern of well-known regeneration-associated repressor genes such as *her4.1* and *insm1a* (Goldman, 2014). RT-PCR and qPCR analysis in cyclopamine-treated retina revealed that the pivotal regeneration-associated genes are downregulated, with the exception of *insm1a* and a few Notch signaling genes (Figures 1N, 1O, and S2F). Insm1a, a known transcriptional repressor in MGPC induction and cell-cycle exit (Ramachandran et al., 2012; Zhang et al., 2009), showed upregulation, whereas levels of *her4.1*, one of the effectors of Notch signaling (Pasini et al., 2004; Wilson et al., 2016), showed downregulation, which was confirmed by mRNA *in situ* hybridization (ISH) and luciferase assays (Figures 1P and 1Q). Upregulation of *insm1a* and downregulation of *her4.1* with blocked Shh signaling in post-injured retina led us to hypothesize the involvement of a well-known transcription factor such as Ascl1a in this regulatory loop. Insm1a, a known transcriptional repressor of *ascl1a* (Ramachandran et al., 2012), could influence its

Figure 1. Shh Signaling Is Necessary for MG Dedifferentiation in the Injured Retina

(A and B) RT-PCR (A) and qPCR (B) analysis of Shh signaling component genes in the retina at indicated time points post-injury; n = 6 biological replicates. *p < 0.001; **p < 0.003.

(C and D) Immunofluorescence (IF) microscopy images of Shh signaling components in wild-type BrdU$^+$ MGPCs (C), and Shh expression in *1016 tuba1a:*GFP transgenic fish at 4 dpi (D). Arrowheads mark protein expression in cells in (C) and (D).

(E) RT-PCR assay of Shh signaling component genes in GFP-positive MGPCs and the rest of the cells from *1016 tuba1a*:GFP transgenic retina at 4 dpi.

(F and G) Bright-field (BF) images of 4-days post-fertilized embryos treated with 5% (v/v) DMSO and 30 μM cyclopamine (F), and quantification of the number of cyclopia embryos (G).

(H–J) IF microscopy images showing a dose-dependent decline in GFP$^+$ and BrdU$^+$ MGPCs in *1016 tuba1a:*GFP transgenic (H) and wild-type (I) retinae, respectively, at 4 dpi upon cyclopamine treatment, which is quantified in (J).

(K) BF microscopy images of blastema during caudal fin regeneration in cyclopamine-treated wild-type zebrafish at 6 days post-amputation.

(L and M) IF microscopy images of retinal sections with *shha* or *sufu* knockdowns (L), and quantification of the number of BrdU$^+$ cells at the injury site (M). *p < 0.0001; n = 4 biological replicates. Lissamine tag on MO shows red fluorescence in (L).

(N–P) RT-PCR analysis of *ascl1a, lin28a, her4.1*, and *insm1a* in uninjured control, 2.5 dpi DMSO-treated, and 2.5 dpi cyclopamine-treated retina (N); qPCR analysis of mRNA levels of *insm1a* and *her4.1* with cyclopamine treatment (O); and BF images of corresponding mRNA *in situ* hybridization (ISH) of these genes in the retina at 4 dpi (P).

(Q) Single-cell-stage embryos were injected with *insm1a:luciferase* or *her4.1:luciferase* vectors along with Renilla luciferase mRNA for normalization and then treated with cyclopamine for 24 hr before lysing for quantification of *insm1a* and *her4.1* promoter activity using a dual luciferase assay.

Scale bars represent 10 μm in (C), (D), (H), (I), (L), and (P) and 500 μm in (F) and (K). Asterisk indicates the injury site (C, H, I, L, and P). Error bars represent SD. *p < 0.0001 (J); *p < 0.001 (M). n = 6 biological replicates. GCL, ganglion cell layer; INL, inner nuclear layer; ONL, outer nuclear layer; UC, uninjured control. See also Figures S1, S2, S6, and S7.

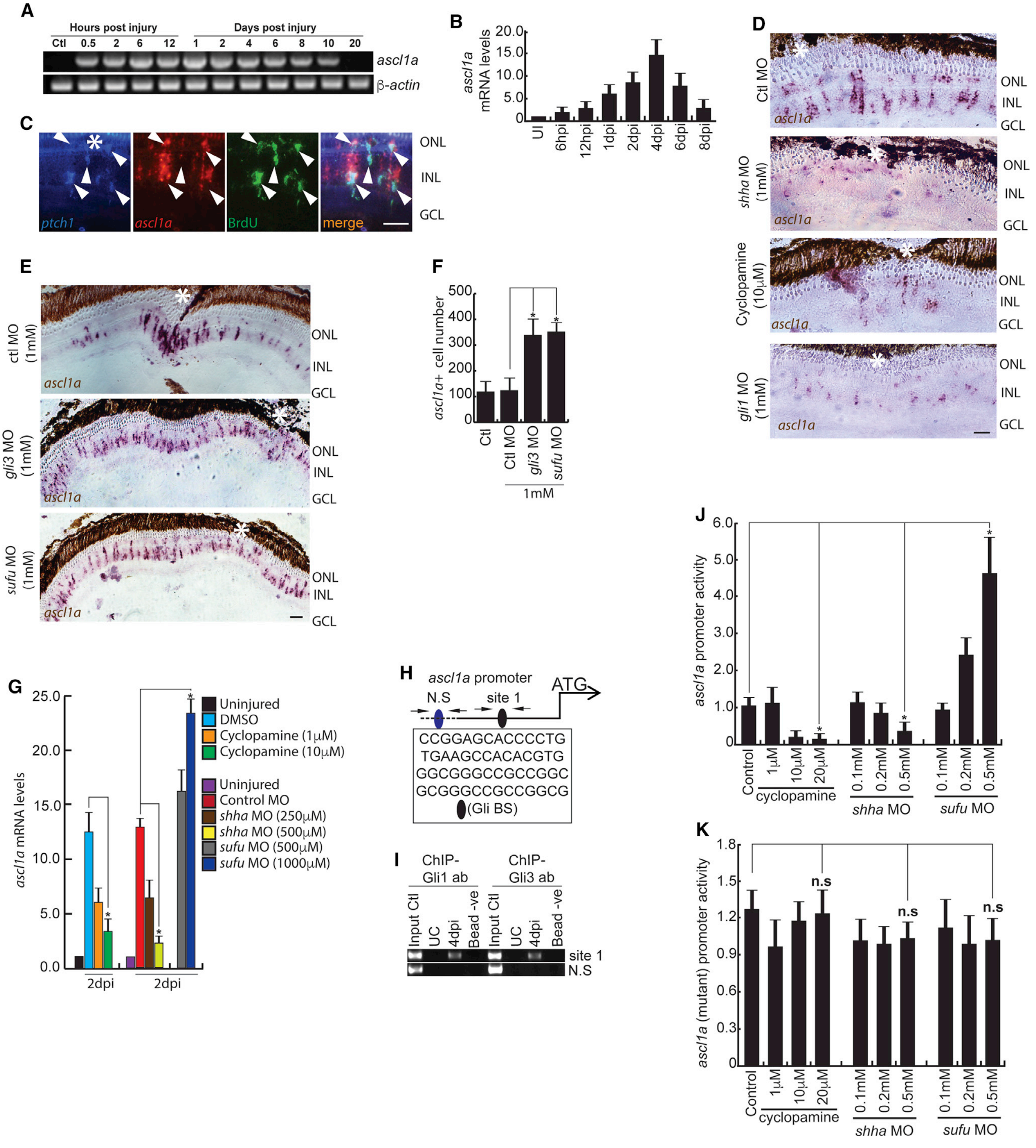

Figure 2. Shh-Signaling-Dependent *ascl1a* Regulation in the Injured Retina

(A and B) RT-PCR (A) and qPCR (B) analysis of *ascl1a* in the post-injured retina; n = 6 biological replicates.

(C) Fluorescence ISH (FISH) and IF microscopy images of a 0.5-μm-thick optical section of retina showing co-localization of *ascl1a* with *ptch1* in BrdU+ MGPCs at 4 dpi. Arrowheads mark co-expression of genes in BrdU+ cells.

(D–F) BF microscopy images of *ascl1a* mRNA ISH in retina at 4 dpi with cyclopamine treatment, *shha* or *gli1* knockdowns (D), and *gli3* or *sufu* knockdowns (E). The number of *ascl1a*+ cells from (E) is quantified in (F).

(G) qPCR analysis of *ascl1a* mRNA with cyclopamine treatment and *shha* or *sufu* knockdown in 2 dpi retina.

(legend continued on next page)

expression in a Shh-signaling-dependent manner. Moreover, Ascl1a could impact the expression of *delta* genes (Henke et al., 2009; Nelson et al., 2009), the ligand of Notch signaling, capable of inducing *her4.1* expression in Notch-expressing cells (Takke et al., 1999). Thus, the Shh-signaling-dependent increase in Insm1a could cause a downregulation of *ascl1a*, which in turn reduces *her4.1* levels in injured retina. These results suggest possible crosstalk between Shh and Notch signaling, contributing to retina regeneration.

Shh Signaling Induces *ascl1a* during Retina Regeneration

Apart from the potential involvement of Insm1a in repressing *ascl1a* levels, we also speculated its direct regulation mediated through Shh signaling. This is presumably true, as the temporal expression pattern of *ascl1a* by RT-PCR and qPCR matched that of Shh signaling components (Figures 1A, 1B, 2A, and 2B). We found the co-expression of *ptch1*, a bona fide marker of active Shh signaling (Jeong and McMahon, 2005), with *ascl1a* mRNA in retina at 4 dpi (Figure 2C). This suggests the potential involvement of Shh signaling in *ascl1a* induction and vice versa. Inhibition of Shh signaling, by cyclopamine treatment or knockdown of *gli1* or *shha*, significantly downregulated *ascl1a* expression (Figures 1N and S2G), which was also confirmed by mRNA ISH and qPCR in retina (Figures 2D and 2G). Conversely, knockdown of negative regulators of Shh signaling, *gli3* and *sufu*, caused an upregulation of *ascl1a* (Figures 2E–2G), suggesting its possible direct regulation. This is supported by the presence of several Gli-binding sites on the *ascl1a* promoter, revealed by *in silico* analysis (Figure 2H). Further, we performed a post-injured retinal chromatin immunoprecipitation (ChIP) assay using antibodies against the Shh signaling effector proteins Gli1 and Gli3 separately to examine whether these Gli-binding sites (Gli-BSs) are functional. Interestingly, both antibodies could separately precipitate Gli-bound chromatin, supporting the direct physical interaction of Gli1/Gli3 on the *ascl1a* promoter (Figures 2I and S2K). Furthermore, a luciferase assay performed in zebrafish embryos confirmed the effect of stimulators and inhibitors of Shh signaling on *ascl1a* expression (Figure 2J). The Gli-BS mutations in the *ascl1a* promoter almost completely abolished the effect of inhibitors and stimulators as revealed by the luciferase assay (Table S2; Figure 2K). These results suggest that Shh signaling regulates the important gene *ascl1a*.

Shh Signaling/*lin28a*/*let-7* Regulatory Loop Is Essential for MGPC Induction

We then explored whether the RNA-binding protein and pluripotency-inducing factor Lin28a, a necessary and well-known target of Ascl1a during retina regeneration, is regulated directly through Shh signaling (Ramachandran et al., 2010a). This was supported by the co-expression of *ptch1* and *lin28a* in 4 dpi retinal sections (Figure 3A), suggesting the possible interdependency or hierarchical regulation. We further evaluated the expression pattern of *lin28a* that goes down with inhibited Shh signaling in retinal cross sections (Figure 3B). This was also proven by qPCR (Figure 3C). The opposite expression pattern of *lin28a* was found with *sufu* knockdown, as expected (Figures 3B and 3C). Evaluation of the *lin28a* promoter revealed putative Gli-BSs (Figure 3D) located as clusters, which were probed using Gli1 and Gli3 antibodies for a ChIP assay in the post-injured retina. Interestingly, both Gli1 and Gli3 bind to one of these Gli-BS clusters (Figures 3E and S2K), suggesting direct regulation of *lin28a* by Gli proteins. These results were further confirmed by luciferase assay performed in zebrafish embryos co-injected with *lin28a:*GFP-luciferase vector along with MOs against positive and negative regulators of Shh signaling (Figure 3F). The introduction of Gli-BS mutations in the *lin28a* promoter alleviated the impact of inhibitors and stimulators as revealed by a luciferase assay (Table S2; Figure 3G). Furthermore, *let-7* microRNA, which is downregulated by Lin28a (Ramachandran et al., 2010a), was abundant in the uninjured inner nuclear layer (INL) in BrdU$^+$ MGPCs at 4 dpi (Figure 3H). This *let-7* downregulation in MGPCs is opposite to the IF pattern of Shh (Figures 3H and 3I), which suggested possible regulation of *shha* mRNA by *let-7* microRNA. The mRNA ISH of *shha* and *ptch1* also revealed a diffused expression pattern in both uninjured and 4 dpi retina (Figures S2H–S2J). *In silico* analysis predicted several *let-7* microRNA-binding sites present in *shha*, *shhb*, *smo*, and *ptch1* genes (Table S4). We cloned these four genes in-frame with GFP reporter regulated by the cytomegalovirus (CMV) promoter and transfected these constructs with increasing concentrations of *let-7a* and *let-7f* microRNA expression plasmid (Ramachandran et al., 2010a) in HEK293T cells (Figure S5F). The results showed a dose-dependent decline in GFP expression (Figure 3J), which was quantified (Figures S6C–S6F). The knockdown of *lin28a* led to an expected decline in Shha protein at 4 dpi (Figure 3K). These findings suggest that *lin28a*-mediated suppression of *let-7* is required for the translational regulation of Shh signaling components in MGPCs as a part of positive feedback loop mediated through the Ascl1a-*lin28a* axis.

Mmp9 Regulates *ascl1a* through Shh Signaling

We also investigated the involvement of *mmp9*, a gene highly induced in regenerating MG cells, as revealed in microarray analysis (Ramachandran et al., 2012) and whole-retina RNA-seq done in the present study. Mmp9 is not only an important enzyme prerequisite for proliferative and pro-differentiative roles (Mannello et al., 2006), but also essential during fin regeneration (LeBert et al., 2015; Yoshinari et al., 2009). We found that *mmp9* is rapidly induced in the injured retina, with a peak expression at

(H) Schematic of the *ascl1a* promoter with a putative Gli-binding site (Gli-BS) cluster. Arrows mark ChIP primers, N.S marks the negative control, and capital letters mark putative Gli-BSs.

(I) Retinal ChIP assay at 4 dpi showing both Gli1 and Gli3 bound to the *ascl1a* promoter.

(J) Luciferase assay in 24 hpf embryos co-injected with *ascl1a:*GFP-*luciferase* vector and *sufu* or *shha* MOs.

(K) Luciferase assay was done with mutated Gli-BS of *ascl1a* promoter in an experiment similar to (J).

Scale bars represent 10 μm in (C) and 20 μm in (D) and (E). Asterisk indicates the injury site (C–E). Error bars represent SD. *p < 0.0001 (F); *p < 0.01 (G); *p < 0.01 (J). n = 6 biological replicates (F and G); n = 3 (J). See also Figures S2, S6, and S7.

OPEN ACCESS
CellPress

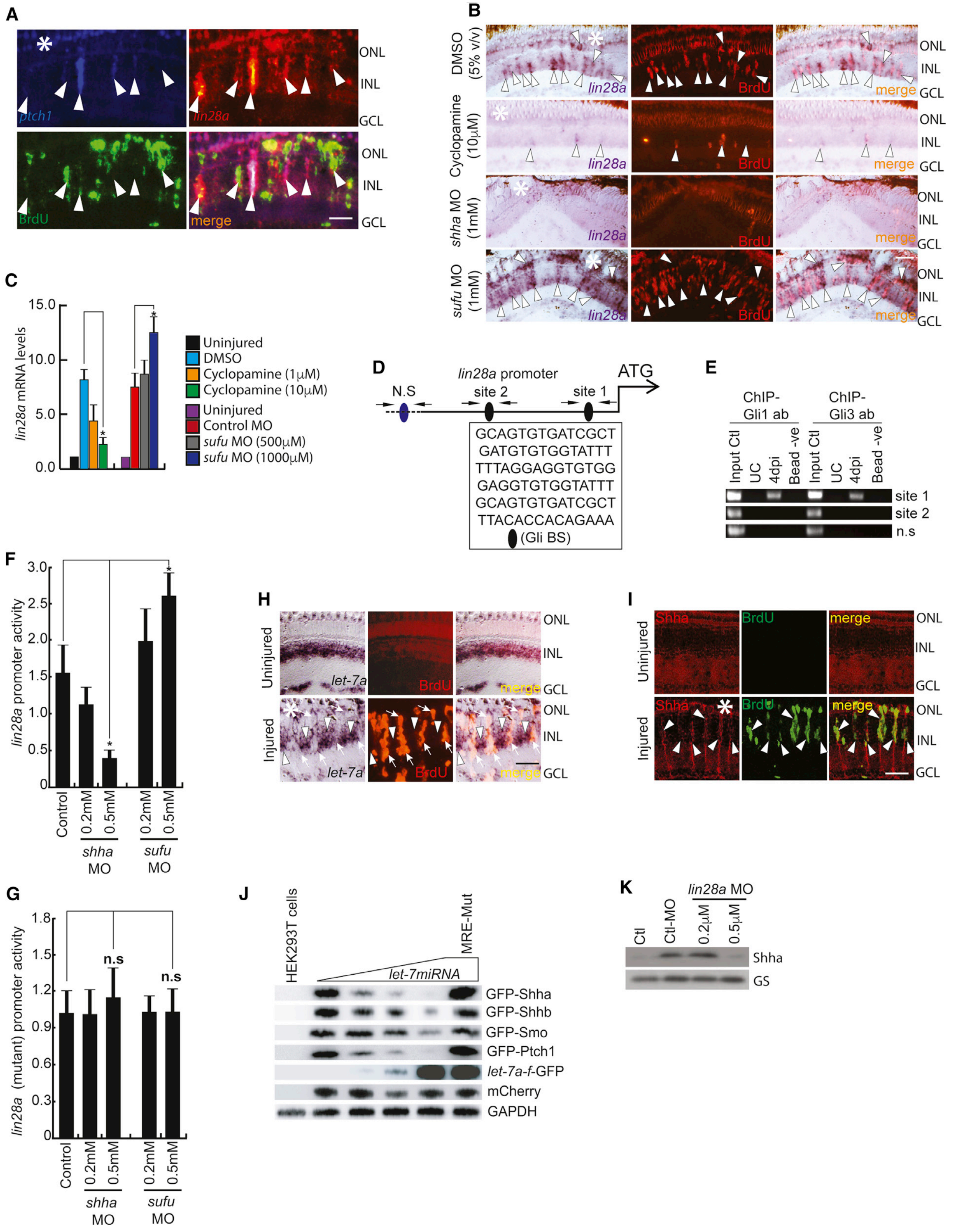

(legend on next page)

24 hpi (Figures 4A and S3A), and later (at 4 dpi), *mmp9* levels were restricted to the neighboring cells of BrdU⁺ MGPCs (Figures S3B and S3C). Interestingly, inhibition of Shh signaling caused a significant upregulation of *mmp9*, and an opposite effect was seen with *sufu* knockdown (Figures 4B and S3D–S3F), which was confirmed by qPCR (Figure 4C) and a luciferase assay performed in zebrafish embryos injected with *mmp9*:GFP-luciferase vector (Figure 4D). These results suggest a negative correlation between *mmp9* and active cell proliferation. However, upon inhibition of Mmp9 using pharmacological agents such as salvianolic acid B and SB-3CT, or by *mmp9* targeting MO (Figures S6A, and S6B; Table S1), we found a drastic decline in $BrdU^+$ cells in WT or GFP^+ cells in *tuba1016* transgenic retina (Figures 4E–4G and S3G). Interestingly, no impact was seen with *mmp9* blockade after 2 dpi (Figure S3H), suggesting that its role preludes cell proliferation. To evaluate this further, we analyzed the expression pattern of an important gene, *ascl1a*, in *mmp9*-expressing cells in 4 dpi retina. We found significant co-localization of $ascl1a^+$ cells with *mmp9* expression (Figures 4H and S4A). Moreover, *mmp9* knockdown caused a decline in *ascl1a* expression, whereas *ascl1a* knockdown caused an upregulation of *mmp9* in 4 dpi retina (Figures 4I and S3I). Since the regulation of *ascl1a* is established through Shh signaling, we further explored whether Mmp9-mediated regulation of *ascl1a* was through Shha. Knockdown of *mmp9* abolished the expression of Shha, as found with cyclopamine treatment (Figures 4J, S3J, and S7B). We also found an Shh-signaling-dependent regulation of Ascl1a protein with both *shha* or *sufu* knockdowns in 2 dpi retina (Figures 4K and S7C). Recombinant-SHH could induce Ascl1a expression and cell proliferation in zebrafish retina, similar to *sufu* knockdown (Figures 4L, S3K–S3M, and S7D). Interestingly, we also found a drastic increase in mRNA levels of *Ascl1*, *Lin28a*, and ASCL1 protein in injured mouse retina treated with recombinant-SHH (Figures 4M, S3N, and S7E).

Inhibition of Notch signaling through *N*-[*N*-(3,5-difluorophenylacetyl)-L-alanyl]-*S*-phenylglycine *t*-butyl ester (DAPT) treatment, which causes a decline in Her4.1 levels and enhancement of MGPCs during retina regeneration (Conner et al., 2014; Wan et al., 2012), increased *mmp9, ascl1a* mRNA, and Shh protein levels (Figures S4B, S4C, 4N, and S7F). We further explored whether *ascl1a* upregulation seen with DAPT treatment is mediated through the Mmp9/Shh axis. Interestingly, we found that in the DAPT-treated retina, *ascl1a* translation was nullified with *mmp9* knockdown (Figures 4O, 4P, and S7G). We speculated that upregulation of *mmp9* with blockade of Notch signaling is possibly due to a lack of Her4.1-mediated transcriptional repression. Expression of *mmp9* and *her4.1* showed co-labeling in a few and co-exclusion in the majority of retinal cells (Figure 4Q). *In silico* analysis of the *mmp9* promoter revealed several *hairy enhancer of split* (Hes/Her)-binding N-boxes (Kageyama et al., 2007), suggesting its potential regulation through Notch signaling (Figure 4R). We performed a luciferase assay in zebrafish embryos co-injected with notch intracellular domain (*nicd*) mRNA along with *mmp9*:GFP-luciferase vector. *nicd* mRNA could cause an upregulation of Her4.1 (Nakahara et al., 2016; Wilson et al., 2016), and the luciferase assay showed dose-dependent downregulation of *mmp9* promoter activity (Figure 4S), while mutations in Her4-binding sites abolished this impact (Figure S4D; Table S2). In summary, these results suggest that active Notch-signaling-mediated induction of *her4.1* restricts the span of *mmp9* expression at the site of injury. Further, Mmp9 coaxes MG to regenerate through Shh signaling and Ascl1a induction during retina regeneration.

Shh Signaling Regulates *zic2b* Expression during Regeneration

We explored a zinc-finger transcription factor, Zic2, essential for normal brain patterning during development (Elms et al., 2003), which upon mutation shows holoprosencephaly (HPE) or cyclopia (Brown et al., 2001; Teslaa et al., 2013), a phenotype similar to cyclopamine treatment. Zic2 is also known to collaborate with Gli proteins (Koyabu et al., 2001). Therefore, we investigated whether a relationship exists between Gli proteins and Zic2 during retina regeneration, because both proteins occupy the same DNA sequence of the target genes' promoters (Vokes et al., 2007). *zic2b*, orthologous to the mammalian Zic2 gene, showed upregulation in the retina microarray (Ramachandran et al., 2012) and our RNA-seq analysis. *zic2b* is also expressed in fin blastema (Figure S4E). The temporal expression pattern of *zic2b* in post-injured retina showed a peak expression at 4 dpi, a time when cell proliferation is at the maximum level (Figure 5A). Pulse labeling of MGPCs with BrdU also revealed its co-localization with *zic2b* (Figure 5B). Co-expression of *ptch1*

Figure 3. Lin28a-*let-7* Axis Regulates Shh Signaling Component Genes in the Injured Retina

(A) FISH and IF microscopy images of a 0.5-μm-thick optical section of retina showed co-localization of *lin28a* with *ptch1* in $BrdU^+$ MGPCs at 4 dpi. Arrowheads mark co-expression of genes in $BrdU^+$ cells.

(B and C) BF microscopy images of *lin28a* mRNA ISH in the retina at 4 dpi with cyclopamine treatment and *shha* or *sufu* knockdown (B), which was quantified by qPCR (C). Arrowheads mark co-expression of genes in $BrdU^+$ cells in (B).

(D and E) Schematic of the *lin28a* promoter with a potential Gli-BS cluster, where arrows mark ChIP primers and capital letters mark consensus sequence of Gli-BS (D). A 4 dpi retinal ChIP assay showed both Gli1 and Gli3 bound to one of the two Gli-BS clusters (E).

(F) Luciferase assay in 24 hpf embryos co-injected with *lin28a:*GFP-*luciferase* vector and *sufu* or *shha* MOs.

(G) Luciferase assay with mutated Gli-BSs of the *lin28a* promoter in an experiment similar to (F).

(H and I) ISH and IF microscopy of retina showing co-exclusion of *let-7a* microRNA (H) and co-localization of Shha protein (I) in $BrdU^+$ MGPCs in the retina at 4 dpi. Arrowheads mark expression of *let-7a* in $BrdU^-$ cells and arrows mark co-exclusion of *let-7a* from $BrdU^+$ cells in (H). Arrowheads mark co-expression of Shha in $BrdU^+$ cells in (I).

(J) *let-7* microRNA downregulated the translation of GFP fused with the indicated gene constructs harboring microRNA-binding regions in a dose-dependent manner in HEK293T cells.

(K) Western blot of Shha in *lin28a*-MO electroporated retina at 4 dpi.

Scale bars represent 10 μm (A, H, and I) and 20 μm (B). Asterisk indicates the injury site (A, B, H, and I). Error bars represent SD.*$p < 0.001$ (C); *$p < 0.001$ (F). n = 6 biological replicates (C, F, and G). GS, glutamine synthetase. See also Figures S3, S6, and S7.

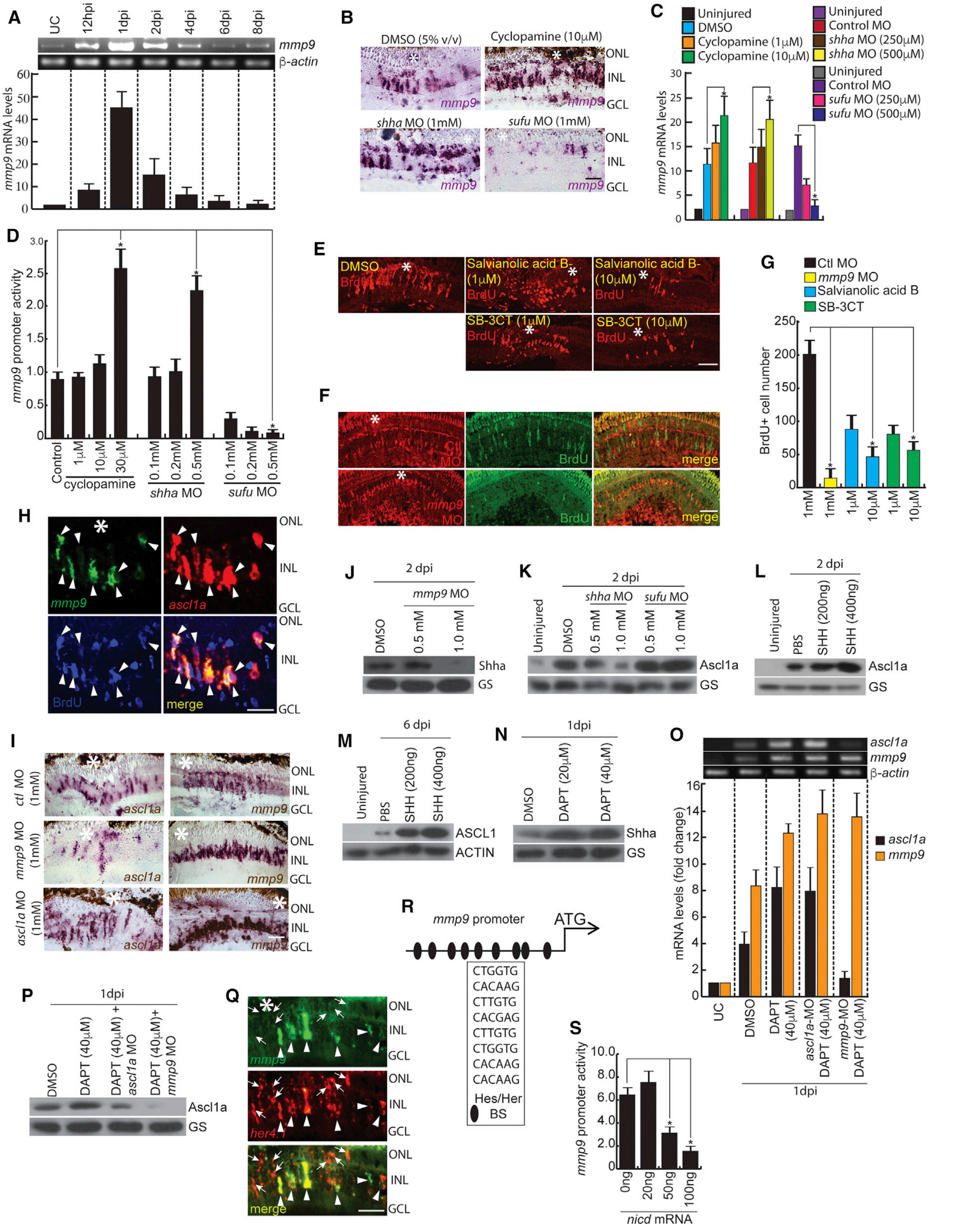

(legend on next page)

with *zic2b* in BrdU$^+$ cells suggests their interaction during regeneration (Figure 5C). The *zic2b* showed downregulation with blockade of Shh signaling and an upregulation with *sufu* knockdown (Figures 5D and 5E). These results were also confirmed by a luciferase assay done in zebrafish embryos injected with *zic2b*:GFP-luciferase construct along with MOs against *shha* and *sufu* and also exposed to cyclopamine (Figure 5F). Analysis of the *zic2b* promoter revealed a cluster of Gli-BSs (Figure 5G), and spanning chromatin was pulled down using both Gli1 and Gli3 antibodies separately (Figures 5H and S2K). Gene knockdowns of *gli1*, *gli3*, and *zic2b* significantly influenced MGPCs proliferation in 4 dpi retina (Figures 5I, 5J, S6A, and S6B; Table S1). The luciferase assay revealed that Shh signaling inhibitors and stimulators had a small impact on *zic2b* promoter activity with mutated Gli-BSs (Table S2; Figure S4F). Early or late knockdowns of *gli1*/*zic2b* caused a decline in the number of BrdU$^+$ cells in the retina, but the opposite was seen with *gli3* knockdown (Figures 5I, 5J, S4G, and S4H). *zic2b* showed a pan retinal expression pattern with DAPT treatment, and the same was seen with *gli3*/*sufu* knockdowns (Figures S4I–S4K). Interestingly, *zic2b* knockdown nullified the enhancement of MGPCs with *gli3* knockdown (Figures 5I and 5J). Moreover, the induction of Gli3 seems to block the responsiveness of MGPCs to Gli1, as the late knockdowns and double knockdown of *gli1* and *gli3* also caused a drastic decline in cell proliferation (Figures 5I, 5J, S4G and S4H). The *gli1* knockdown significantly impacted several regeneration-associated genes as the possible cause of the lack of MGPC induction (Figure S4L). These results suggest that the induction of *zic2b* in MGPCs largely triggers a proliferative phase mediated through Shh signaling, and it may collaborate with or outcompete Gli proteins in targeting Gli-BSs to drive MGPCs toward differentiation.

We also examined whether *zic2b* expression depends on the *mmp9-shha-ascl1a* signaling axis, because a substantial proportion of BrdU$^+$ MGPCs co-expressed *ascl1a* and *zic2b* (Figure 5K). We probed for *zic2b* expression in 4 dpi retina electroporated with *mmp9* and *ascl1a* MOs separately and found that *zic2b* levels declined drastically, as found with blockade of Shh signaling (Figures 5D and 5L). We further speculated that apart from its transcriptional control, *zic2b* might be regulated at translational levels. This speculation is mainly because of the presence of bona fide *let-7* microRNA-binding sites in the *zic2b* coding region (Figure S5F). Surprisingly, we found a downregulation in the translation of GFP protein from an expression cassette appended with *zic2b* in HEK293T cells (Figure 5M), which was quantified (Figure S6G). These results suggest that *zic2b* is an essential regeneration-associated gene in zebrafish retina that is regulated through the *mmp9-shha-ascl1a-lin28a-let-7* pathway.

The Foxn4/Ascl1a/Shh/Zic2b Regulatory Loop Is Associated with Regeneration

Foxn4, a member of the forkhead box family of proteins and discovered in retina microarray (Ramachandran et al., 2012) and RNA-seq analyses performed in the present study, showed an upregulation, with a peak expression at 4 dpi (Figures 6A and 6B). Foxn4 expression was restricted to BrdU$^+$ MGPCs at 4 dpi (Figure 6C). Furthermore, we explored the significance of *foxn4* induction during retina regeneration. Interestingly, MO-mediated gene knockdown of *foxn4* inhibited MGPC induction up to 90% (Figures 6D, 6E, and S5A).

To ascertain whether *foxn4* is regulated through Shh signaling or its downstream effector genes, we adopted a pharmacological inhibition or gene-knockdown approach. Blockade of Shh signaling with cyclopamine or MOs against *shha* or *gli1* significantly abolished *foxn4* expression in the retina (Figures 6F, S4L, and S5B), whereas the opposite was seen with *sufu* knockdown (Figures S5C and S5D). Analysis of the *foxn4* promoter revealed 2 putative Gli-BS clusters (Figure 6G) that were strongly bound by Gli1 and Gli3, as revealed by a ChIP assay (Figures 6H and S2K), suggesting a direct involvement of Shh signaling in its expression. As discussed earlier, the influence of Mmp9 on expression levels of Shha led us to suspect its involvement in the regulation of *foxn4*. Knockdown of *mmp9* in 4 dpi retina caused a significant downregulation of *foxn4* (Figures 6I and S5E).

Figure 4. Shh-Mmp9-Ascl1a Interplay Is Necessary during MG Reprogramming

(A) RT-PCR (top) and qPCR (bottom) analysis of injury-dependent *mmp9* expression in the retina; n = 6 biological replicates.

(B–D) BF microscopy images of *mmp9* mRNA ISH in the retina at 4 dpi with cyclopamine treatment and *shha* or *sufu* knockdown (B), as quantified by qPCR (C), and a luciferase assay in 24 hpf embryos injected with *mmp9:*GFP-*luciferase* vector (D).

(E–G) IF microscopy images of 4 dpi retina with Mmp9 blockade using drugs (E) and MO against *mmp9* (F). The number of BrdU$^+$ MGPCs is quantified in (G).

(H) FISH and IF microscopy images of a 0.5-μm-thick optical section of retina showing co-localization of *mmp9* and *ascl1a* in BrdU$^+$ MGPCs at 4 dpi. Arrowheads mark co-expression of genes in BrdU$^+$ cells.

(I) BF microscopy images of *ascl1a* and *mmp9* mRNA ISH in *ascl1a* and *mmp9* knockdowns in 4 dpi retina.

(J) Western blotting experiment showing Shh levels in 2 dpi retina with the *mmp9* knockdown.

(K) Western blotting assay of Ascl1a in 2 dpi retina with *shha or sufu* knockdowns.

(L) Western blotting assay of Ascl1a in 2 dpi zebrafish retina injected with recombinant SHH protein.

(M) Western blotting assay of ASCL1 in 6 dpi mouse retina injected with recombinant SHH protein.

(N) Western blotting assay of Shha in DAPT-treated retina at 1dpi.

(O and P) RT-PCR (top) and qPCR (bottom) analysis of *ascl1a* and *mmp9* in DAPT-treated retina, with or without *ascl1a* or *mmp9* knockdown (O), and confirmed by western blotting assay (P).

(Q) FISH and IF microscopy images of a 0.5-μm-thick optical section of retina showed substantial co-exclusion and marginal co-localization of *mmp9* with *her4.1* at 4 dpi. Arrowheads mark co-expression of the gene, and arrows mark *her4.1*$^+$ cells.

(R and S) Schematic of the *mmp9* promoter with potential Hes/Her-BS binding sites (inside box), and luciferase assay in 24 hpf embryos co-injected with *mmp9:*GFP-*luciferase* construct and notch intracellular domain (*nicd*) mRNA (S).

Scale bars represent 10 μm (H and Q) and 20 μm (B, E, F, and I). Asterisk indicates the injury site (B, E, F, H, I and Q). Error bars represent SD. *p < 0.001 (C, D, G, and S). Biological replicates n = 6 in (C) and (G), and n = 3 in (D) and (S). See also Figures S3, S4, S6, and S7.

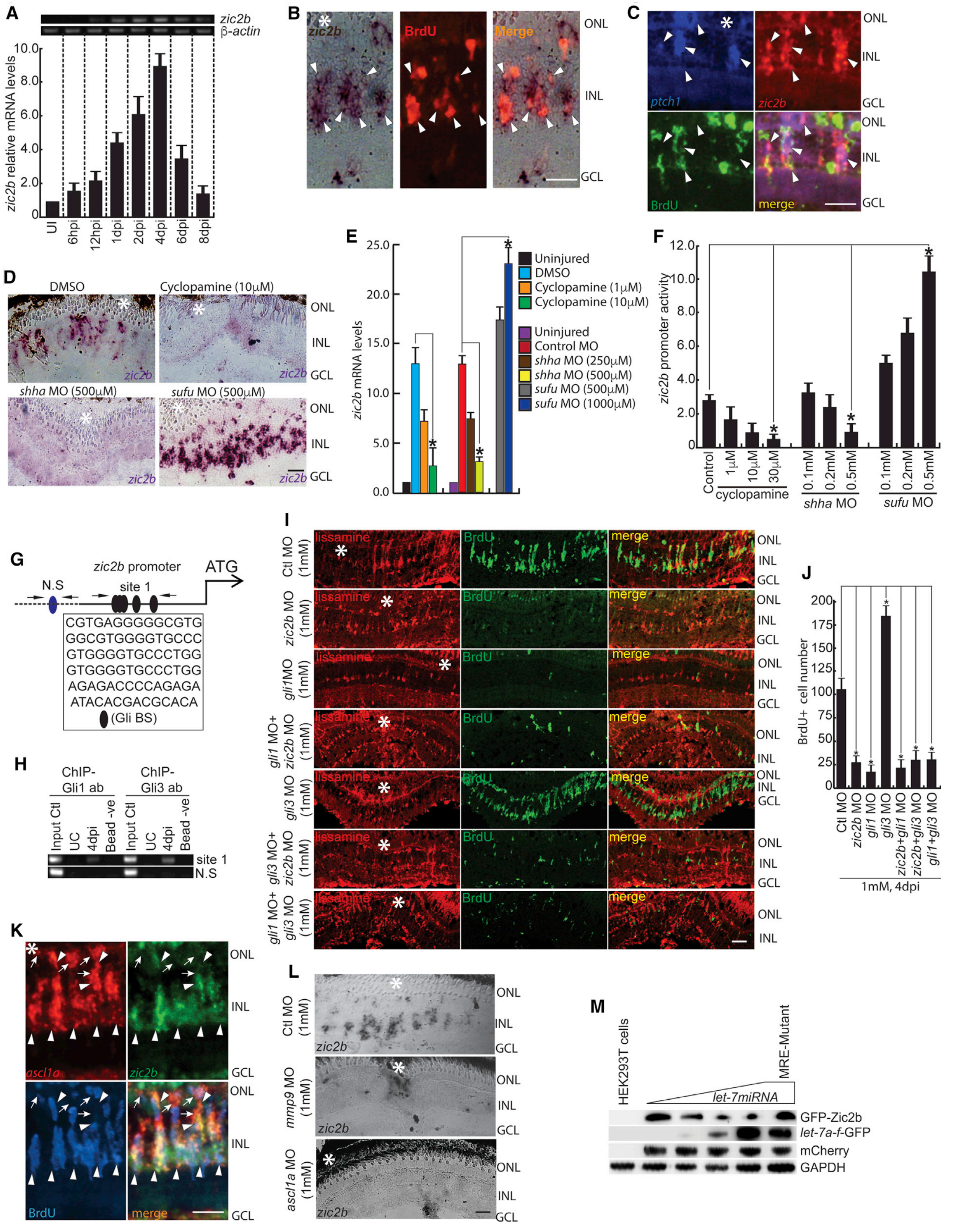

(legend on next page)

The temporal gene expression pattern and co-localization of *foxn4* with MGPCs prompted us to investigate its potential parallels with *ascl1a* gene. Fluorescence ISH (FISH) analysis showed co-expression of *ascl1a* and *foxn4* in $BrdU^+$ MGPCs (Figure 6J). We then explored the possibility of a hierarchical regulation between *ascl1a* and *foxn4* during retina regeneration, as there is already a reported role for Foxn4 in the regulation of Ascl1 expression in mouse and chick (Del Barrio et al., 2007). We found significant downregulation of *foxn4* expression in retinal sections with knockdown of *ascl1a* (Figure 6I). *foxn4* promoter analysis predicted several Ascl1a-binding E-boxes (Bertrand et al., 2002; Li et al., 2006; Ramachandran et al., 2010a, 2011), and binding was confirmed by a ChIP assay (Figures 6K, 6L, and S5G). The transactivation of the *foxn4* promoter by Ascl1a was confirmed with a luciferase assay, which was done by co-injection of *ascl1a* mRNA or MO against it, along with the promoter of *foxn4* driving the GFP-luciferase fusion construct in zebrafish embryos (Figure 6M). The mutation of Ascl1a-BS in the *foxn4* promoter had a negligible effect on its promoter activity both by *ascl1a* mRNA or by MO co-injections in zebrafish embryos (Figure S5H; Table S2).

We then explored, using a knockdown approach in the retina, whether Foxn4 impacted *ascl1a* or other regeneration-associated genes such as *zic2b* and *mmp9*. We found that both *ascl1a* and *zic2b* were downregulated, which also explained the downregulation of *foxn4* itself, whereas no appreciable change was seen in *mmp9* levels (Figure 6N). A luciferase assay confirmed transactivation of the *ascl1a* promoter by Foxn4, which was done by co-injection of *foxn4* mRNA or MO against it, along with the promoter of *ascl1a* driving the GFP-luciferase fusion construct in zebrafish embryos (Figure 6O). Both the *ascl1a* and *zic2b* promoters harbor 2 potential Foxn4-binding sites (Luo et al., 2012) (Figure 6P), and this was confirmed by a ChIP assay, which was done using an antibody targeting Foxn4 (Figure 6Q). Mutated Foxn4-BS on the *ascl1a* promoter caused an almost complete alleviation of upregulated luciferase activity, as seen by its overexpression (Figures 6O and S5I; Table S2). These results suggest that *foxn4* expression is dependent on Shh signaling directly as well as through other genes such as *ascl1a*, which in turn regulates another regeneration-associated gene such as *zic2b* in a feedback loop. The findings from this study are summarized in a model (Figures 7A and 7B).

DISCUSSION

In this study, we explored the significance and potential regulators of Shh signaling during zebrafish retina regeneration. Our findings unravel mechanisms through which Shh signaling contributes to retina regeneration. We propose that Shh-dependent induction of Ascl1a and Lin28a contributes to Müller glia dedifferentiation through *let-7* microRNA-mediated translational downregulation of *shha*, *shhb*, *smo*, *ptch1*, and *zic2b* from respective mRNAs. Such stringent translational regulation probably accounts for the lack of an immature regenerative response despite the marginal expression of Shh signaling components such as *shha*, *shhb*, *smo*, and *ptch* in the uninjured retina. Cyclopamine-mediated repression of MGPCs might result from a decline in the regeneration-specific genes *ascl1a* and *lin28a*. This situation could be further exacerbated by upregulation of the repressor *insm1a* and the lack of the Delta-Notch signaling effector *her4.1*. These observations suggest the ability of Shh signaling to impinge upon various other signaling pathways important for regeneration.

Our results also show that Shh signaling impacted regeneration not only through transcription factors but also through negative regulation of enzymes such as Mmp9. Moreover, Mmp9-dependent expression of Shha causes the induction of Ascl1a as a prelude to MG dedifferentiation and MGPC induction. The increased expression of Mmp9 in a regeneration-compromised scenario like cyclopamine treatment (*shha* or *ascl1a* knockdown retina) suggests the existence of a feedback loop between Mmp9 and Shh signaling. The abundance of Mmp9 is probably due to the lack of Shha protein to give a feedback response for a decrease in its expression in MG to induce MGPCs. This observation is also supported by the *sufu* knockdown-mediated decline in *mmp9* expression. Co-labeling of *ascl1a* and *mmp9*, which was seen in a good number of cells, may appear paradoxical, but they all need not be Shh-positive or $BrdU^+$. Only a subset of *ascl1a*-positive cells is *ptch1* positive and can have active Shh

Figure 5. The Shh-Mediated Zic2b Axis Is Necessary during Retina Regeneration

(A) RT-PCR (top) and qPCR (bottom) analysis of injury-dependent *zic2b* expression in the retina; n = 6 biological replicates.

(B) ISH and IF microscopy revealed co-localization of *zic2b* mRNA with $BrdU^+$ MGPCs in 4 dpi retina.

(C) FISH and IF microscopy images of a 0.5-μm-thick optical section of retina showing co-localization of *zic2b* with *ptch1* in $BrdU^+$ MGPCs at 4 dpi.

(D and E) BF microscopy images of *zic2b* mRNA ISH in 4 dpi retina, with cyclopamine treatment, MO mediated *shha* or *sufu* knockdown done separately (D), which is quantified in (E).

(F) Luciferase assay in 24 hpf embryos injected with *zic2b:*GFP-*luciferase* vector with cyclopamine treatment and *shha* or *sufu* knockdowns.

(G) Schematic of the *zic2b* promoter with a putative Gli-BS. Arrows mark ChIP primers, N.S marks negative control devoid of Gli-BSs, and capital letters mark consensus of Gli-BSs.

(H) Retinal ChIP assay at 4 dpi showing both Gli1 and Gli3 bound to the *zic2b* promoter.

(I) IF microscopy images of $BrdU^+$ cells in the regenerating retina with *zic2b*, *gli1*, and *gli3* knockdowns in isolation or combination, delivered at the time of injury, compared with control MO.

(J) $BrdU^+$ cells are quantified in the indicated knockdowns.

(K) FISH and IF microscopy images of a 0.5-μm-thick optical section of retina showing co-localization of *zic2b* with *ascl1a* in $BrdU^+$ MGPCs at 4 dpi. Arrowheads indicate *ascl1a* and *zic2b* co-expression, whereas arrows indicate $ascl1a^+$ but $zic2b^-$ cells.

(L) ISH microscopy retinal images of *zic2b* mRNA with *mmp9* or *ascl1a* knockdown at 4 dpi.

(M) *let-7* microRNA downregulated translation of the GFP construct appended with *zic2b* harboring microRNA responsive regions in a dose-dependent manner in HEK293T cells.

Scale bars represent 10 μm (B, C, and K) and 20 μm (D, I, and L). Asterisk indicates the injury site (B, C, D, I, K, and L). Error bars represent SD. *p < 0.001 (E, F, and J). n = 6 biological replicates (E and J); n = 3 (F). See also Figures S4–S7.

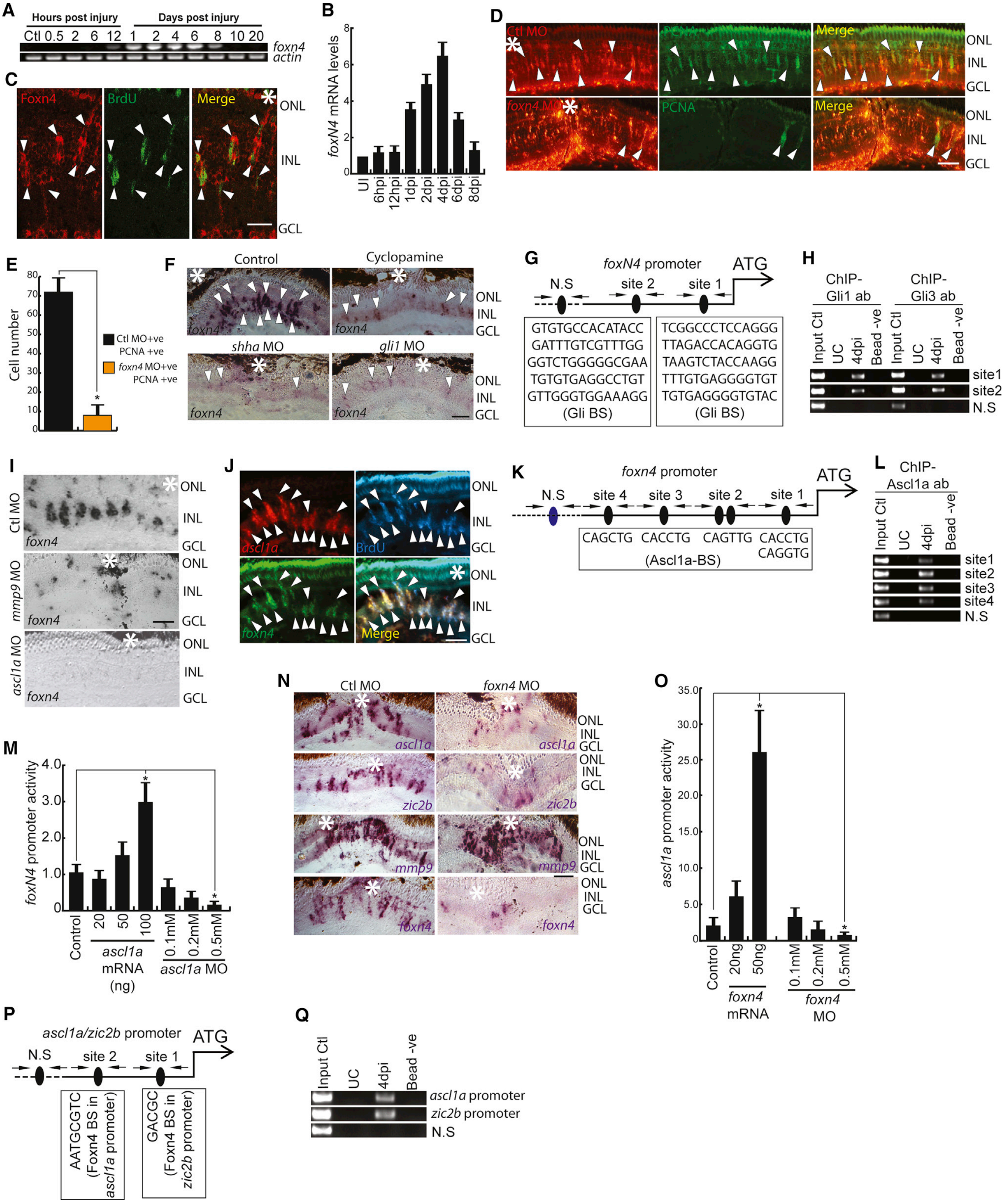

Figure 6. Expression Dynamics and Necessity of Foxn4 during Regeneration

(A and B) RT-PCR (A) and qPCR (B) analysis of injury-dependent *foxn4* expression in the retina; n = 6 biological replicates. (C) IF microscopy of a 0.5-μm-thick optical section of retina revealing co-localization of Foxn4 with BrdU$^+$ MGPCs in 4 dpi retina.

(legend continued on next page)

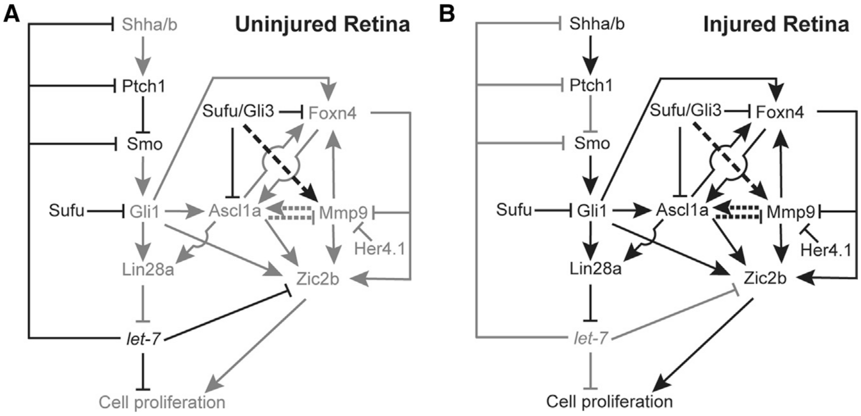

Figure 7. Schematic Representation of the Gene Regulatory Network during Retina Regeneration

(A and B) Genetic interrelationships in uninjured (A) and injured (B) retina. Faded arrows and gene names show absence and bold shows presence. See also Figures S1–S7.

signaling and downregulated *mmp9*. The remainder of the *ascl1a* positive cells can have upregulated *mmp9* due to the lack of Shh signaling. Moreover, the Mmp9 expression is necessary for normal cycling of MGPCs during regeneration, and the repression of *mmp9* by Her4.1 could enable its expression restricted to the injury site at a later time. We anticipate a much wider role for the Shha-Mmp9-Ascl1a-Lin28a-*let-7* regulatory loop during retinal regeneration.

The induction of repressor Gli3 might cause the exit of MGPCs from the cell cycle to restrict the impact of a transcriptional activator, Gli1. This is evident from the knockdown results of *gli1* and *gli3* either in isolation or in combination. The *gli1* knockdown indicated a decline in the number of MGPCs, whereas *gli3* inhibition caused an expansion of MGPCs. Interestingly, double knockdown of *gli1* and *gli3* resulted in significant decline in MGPCs, suggesting that the Gli3 is necessary to quit the cell cycle as a prelude to differentiation. Similar results were seen with *zic2b* knockdown or cyclopamine treatment. This could be due to the impact of Shh signaling on the expression of downstream genes through Zic2b, although both Gli and Zic2b may compete or collaborate with the same binding sites on DNA. As *zic2b* mRNA shows a translational regulation through *let-7* microRNA, one could speculate that the role of Zic2b protein is restricted to Ascl1a- or Lin28a-expressing MGPCs.

The forkhead box gene family member *foxn4* is unique in its expression pattern during zebrafish development, with multiple isoforms in the thymus, skin, and brain (Danilova et al., 2004). We show the brain-specific isoform of *foxn4* is rapidly induced by Shh signaling, which orchestrates a series of gene expression events in response to retinal injury. Gli-BSs on the *foxn4* promoter is functional and probably explains the lack of its expression in the cyclopamine-treated retina. The regeneration-associated transcription factor Ascl1a significantly contributes to the induction of *foxn4*, suggesting dual control of its expression. Moreover, Foxn4 deficiency caused a significant reduction in MGPC number, probably through its effect on other regeneration-associated genes, which form a regulatory loop. To support this view, the proof that FoxN4 binds to promoters of *ascl1a* and *zic2b* at its consensus-binding sites (obtained from ChIP) makes it one of the central pillars of regeneration.

Taken together, our study sheds light on the mechanisms of MGPC induction in zebrafish retina in response to injury in an Shh-signaling-dependent manner and the significance of its downstream effector genes such as *ascl1a*, *lin28a*, *zic2b*, *foxn4*, and *mmp9*. These findings also suggest ways to coax mammalian MG dedifferentiation that may enable us to find ample solutions to intervene therapeutically for an efficient regenerative response.

EXPERIMENTAL PROCEDURES

Further details and an outline of resources used in this work can be found in Supplemental Experimental Procedures.

Animals and Retinal Injury

Zebrafish were maintained at 26–28°C on a 14 hr/10 hr light/dark cycle for all experiments unless otherwise specified. The retinal injury was performed

(D and E) IF microscopy images of the retina with *foxn4* knockdown at 4 dpi (D). The number of PCNA$^+$ MGPCs is quantified in (E).

(F) BF microscopy images of *foxn4* mRNA ISH in retinal sections with cyclopamine treatment and *shha* or *gli1* knockdowns.

(G and H) Schematic of *foxn4* promoter with a putative Gli-BS cluster, where arrows mark ChIP primers, N.S marks negative control, and capital letters mark putative Gli-BSs (G). A retinal ChIP assay at 4 dpi showing both Gli1 and Gli3 bound to the *foxn4* promoter (H).

(I) BF microscopy images of *foxn4* mRNA ISH in retinal sections with *mmp9* or *ascl1a* knockdowns.

(J) FISH and IF microscopy images of a 0.5-μm-thick optical section of retina showing co-localization of *foxn4* and *ascl1a* in BrdU$^+$ MGPCs at 4 dpi. Arrowheads mark co-expression of genes in BrdU$^+$ cells.

(K and L) Schematic of the *foxn4* promoter with a putative Ascl1a-binding site cluster, where arrows mark ChIP primers, N.S marks negative control, and capital letters mark putative Ascl1a-BS (K). A retinal ChIP assay at 4 dpi showing Ascl1a bound to the *foxn4* promoter (L).

(M) Luciferase assay showing *foxn4* promoter activity with overexpression or knockdown of *ascl1a* in 24 hpf embryos.

(N) BF microscopy images of mRNA ISH in retinal sections with *foxn4* knockdown showing levels of genes (namely, *ascl1a*, *zic2b*, *mmp9*, and *foxn4*) at 4 dpi.

(O) Luciferase assay showing *ascl1a* promoter activity with overexpression or knockdown of *foxn4* in 24 hpf embryos.

(P and Q) Schematic of *ascl1a* and *zic2b* promoter with a putative Foxn4-binding site cluster, where arrows mark ChIP primers, N.S marks negative control, and capital letters mark putative Foxn4-BS (P). A retinal ChIP assay at 4 dpi showing Foxn4 bound to both the *ascl1a* and *zic2b* promoters (Q).

Scale bars represent 10 μm (C, D, F, I, J, and N). Error bars represent SD. $^*p < 0.001$ (M); $^*p < 0.04$ (O). Biological replicates n = 6 in (M) and O, and n = 3 in (B). Asterisk marks injury spots in (C),(D),(F), (J) and (N). See also Figures S5–S7.

using a 30G needle as described previously (Fausett and Goldman, 2006). The C57BL/6 mice used in this study were maintained on a 12 hr/12 hr light/dark cycle with continuous access to food and water.

RNA-Seq Analysis

The RNA-seq analysis of the total RNA of the retina at different time points post-injury and with cyclopamine treatment was performed as described previously (Brooks et al., 2012).

Statistical Analysis

Observed data were analyzed for statistical significance by comparisons done using a two-tailed unpaired Student's t test to analyze data from all experiments. Error bars represent SD in all histograms.

DATA AND SOFTWARE AVAILABILITY

The accession number for the RNA-seq data reported in this paper is GEO: GSE102063.

SUPPLEMENTAL INFORMATION

Supplemental Information includes Supplemental Experimental Procedures, seven figures, and five tables and can be found with this article online at https://doi.org/10.1016/j.celrep.2018.04.002.

ACKNOWLEDGMENTS

This work was supported by the Department of Biotechnology (DBT) (S.K.), the Indian Council of Medical Research (S.G.), IISER Mohali (S.M., M.A.K., and M.C.), the Wellcome Trust/DBT India Alliance (Intermediate Fellowship IA/I/12/2/500630, to R.R.), extramural research funding from DBT India (102/IFD/SAN/3975/2015-2016 and 102/IFD/SAN/2255/2017-2018, to R.R.), and intramural funding support from IISER Mohali. The authors would like to express their gratitude to Daniel Goldman (Molecular and Behavioral Neuroscience Institute, University of Michigan) for sharing promoter clones of *1016tuba1a*, *lin28a*, *ascl1a*, and *insm1a* genes. The authors also thank Dr. Kuljeet Singh Sandhu and Mr. Arashdeep Singh (IISER Mohali) for their help predicting Gli-BSs, Dr. Mahak Sharma's laboratory (IISER Mohali) for cell transfection experiments, and Dr. Adrene Freeda D'cruz and Dr. Rayman Matharu (IISER Mohali) for critical reading of the manuscript.

AUTHOR CONTRIBUTIONS

R.R. conceived the study and designed experiments. S.K. performed the majority of experiments. S.G. and M.C. contributed to western blotting assays. M.A.K. conducted the fin experiments, S.M. performed RNA-seq Venn diagrams, and A.J.K. helped with cell sorting. R.R., S.K., and S.M. analyzed the experimental data. R.R. wrote the manuscript with critical input from S.K., S.G., M.C., and S.M.

DECLARATION OF INTERESTS

The authors declare no competing interests.

Received: October 13, 2017
Revised: February 3, 2018
Accepted: March 30, 2018
Published: May 1, 2018

REFERENCES

Ando, K., Shibata, E., Hans, S., Brand, M., and Kawakami, A. (2017). Osteoblast production by reserved progenitor cells in zebrafish bone regeneration and maintenance. Dev. Cell *43*, 643–650.

Bertrand, N., Castro, D.S., and Guillemot, F. (2002). Proneural genes and the specification of neural cell types. Nat. Rev. Neurosci. *3*, 517–530.

Brooks, M.J., Rajasimha, H.K., and Swaroop, A. (2012). Retinal transcriptome profiling by directional next-generation sequencing using 100 ng of total RNA. Methods Mol. Biol. *884*, 319–334.

Brown, L.Y., Odent, S., David, V., Blayau, M., Dubourg, C., Apacik, C., Delgado, M.A., Hall, B.D., Reynolds, J.F., Sommer, A., et al. (2001). Holoprosencephaly due to mutations in ZIC2: alanine tract expansion mutations may be caused by parental somatic recombination. Hum. Mol. Genet. *10*, 791–796.

Chen, J.K., Taipale, J., Cooper, M.K., and Beachy, P.A. (2002). Inhibition of Hedgehog signaling by direct binding of cyclopamine to Smoothened. Genes Dev. *16*, 2743–2748.

Conner, C., Ackerman, K.M., Lahne, M., Hobgood, J.S., and Hyde, D.R. (2014). Repressing notch signaling and expressing TNFα are sufficient to mimic retinal regeneration by inducing Müller glial proliferation to generate committed progenitor cells. J. Neurosci. *34*, 14403–14419.

Danilova, N., Visel, A., Willett, C.E., and Steiner, L.A. (2004). Expression of the winged helix/forkhead gene, foxn4, during zebrafish development. Brain Res. Dev. Brain Res. *153*, 115–119.

Del Barrio, M.G., Taveira-Marques, R., Muroyama, Y., Yuk, D.I., Li, S., Wines-Samuelson, M., Shen, J., Smith, H.K., Xiang, M., Rowitch, D., and Richardson, W.D. (2007). A regulatory network involving Foxn4, Mash1 and delta-like 4/Notch1 generates V2a and V2b spinal interneurons from a common progenitor pool. Development *134*, 3427–3436.

Dunaeva, M., and Waltenberger, J. (2017). Hh signaling in regeneration of the ischemic heart. Cell. Mol. Life Sci. *74*, 3481–3490.

Elms, P., Siggers, P., Napper, D., Greenfield, A., and Arkell, R. (2003). Zic2 is required for neural crest formation and hindbrain patterning during mouse development. Dev. Biol. *264*, 391–406.

Fausett, B.V., and Goldman, D. (2006). A role for alpha1 tubulin-expressing Müller glia in regeneration of the injured zebrafish retina. J. Neurosci. *26*, 6303–6313.

Goldman, D. (2014). Müller glial cell reprogramming and retina regeneration. Nat. Rev. Neurosci. *15*, 431–442.

Henke, R.M., Meredith, D.M., Borromeo, M.D., Savage, T.K., and Johnson, J.E. (2009). Ascl1 and Neurog2 form novel complexes and regulate Delta-like3 (Dll3) expression in the neural tube. Dev. Biol. *328*, 529–540.

Hochedlinger, K., and Plath, K. (2009). Epigenetic reprogramming and induced pluripotency. Development *136*, 509–523.

Incardona, J.P., Gaffield, W., Kapur, R.P., and Roelink, H. (1998). The teratogenic Veratrum alkaloid cyclopamine inhibits sonic hedgehog signal transduction. Development *125*, 3553–3562.

Jeong, J., and McMahon, A.P. (2005). Growth and pattern of the mammalian neural tube are governed by partially overlapping feedback activities of the hedgehog antagonists patched 1 and Hhip1. Development *132*, 143–154.

Kageyama, R., Ohtsuka, T., and Kobayashi, T. (2007). The Hes gene family: repressors and oscillators that orchestrate embryogenesis. Development *134*, 1243–1251.

Koyabu, Y., Nakata, K., Mizugishi, K., Aruga, J., and Mikoshiba, K. (2001). Physical and functional interactions between Zic and Gli proteins. J. Biol. Chem. *276*, 6889–6892.

LeBert, D.C., Squirrell, J.M., Rindy, J., Broadbridge, E., Lui, Y., Zakrzewska, A., Eliceiri, K.W., Meijer, A.H., and Huttenlocher, A. (2015). Matrix metalloproteinase 9 modulates collagen matrices and wound repair. Development *142*, 2136–2146.

Li, J., Liu, Q., Qiu, M., Pan, Y., Li, Y., and Shi, T. (2006). Identification and analysis of the mouse basic/Helix-Loop-Helix transcription factor family. Biochem. Biophys. Res. Commun. *350*, 648–656.

Luo, H., Jin, K., Xie, Z., Qiu, F., Li, S., Zou, M., Cai, L., Hozumi, K., Shima, D.T., and Xiang, M. (2012). Forkhead box N4 (Foxn4) activates Dll4-Notch signaling to suppress photoreceptor cell fates of early retinal progenitors. Proc. Natl. Acad. Sci. USA *109*, E553–E562.

Mannello, F., Tonti, G.A., Bagnara, G.P., and Papa, S. (2006). Role and function of matrix metalloproteinases in the differentiation and biological characterization of mesenchymal stem cells. Stem Cells *24*, 475–481.

Miller, S.G., Carnell, L., and Moore, H.H. (1992). Post-Golgi membrane traffic: brefeldin A inhibits export from distal Golgi compartments to the cell surface but not recycling. J. Cell Biol. *118*, 267–283.

Nakahara, Y., Muto, A., Hirabayashi, R., Sakuma, T., Yamamoto, T., Kume, S., and Kikuchi, Y. (2016). Temporal effects of Notch signaling and potential cooperation with multiple downstream effectors on adenohypophysis cell specification in zebrafish. Genes Cells *21*, 492–504.

Nelson, B.R., Hartman, B.H., Ray, C.A., Hayashi, T., Bermingham-McDonogh, O., and Reh, T.A. (2009). Acheate-scute like 1 (Ascl1) is required for normal delta-like (Dll) gene expression and notch signaling during retinal development. Dev. Dyn. *238*, 2163–2178.

Pasini, A., Jiang, Y.J., and Wilkinson, D.G. (2004). Two zebrafish Notch-dependent hairy/Enhancer-of-split-related genes, her6 and her4, are required to maintain the coordination of cyclic gene expression in the presomitic mesoderm. Development *131*, 1529–1541.

Ramachandran, R., Fausett, B.V., and Goldman, D. (2010a). Ascl1a regulates Müller glia dedifferentiation and retinal regeneration through a Lin-28-dependent, let-7 microRNA signalling pathway. Nat. Cell Biol. *12*, 1101–1107.

Ramachandran, R., Reifler, A., Parent, J.M., and Goldman, D. (2010b). Conditional gene expression and lineage tracing of tuba1a expressing cells during zebrafish development and retina regeneration. J. Comp. Neurol. *518*, 4196–4212.

Ramachandran, R., Zhao, X.F., and Goldman, D. (2011). Ascl1a/Dkk/beta-catenin signaling pathway is necessary and glycogen synthase kinase-3beta inhibition is sufficient for zebrafish retina regeneration. Proc. Natl. Acad. Sci. USA *108*, 15858–15863.

Ramachandran, R., Zhao, X.F., and Goldman, D. (2012). Insm1a-mediated gene repression is essential for the formation and differentiation of Müller glia-derived progenitors in the injured retina. Nat. Cell Biol. *14*, 1013–1023.

Sherpa, T., Fimbel, S.M., Mallory, D.E., Maaswinkel, H., Spritzer, S.D., Sand, J.A., Li, L., Hyde, D.R., and Stenkamp, D.L. (2008). Ganglion cell regeneration following whole-retina destruction in zebrafish. Dev. Neurobiol. *68*, 166–181.

Sun, L., Li, P., Carr, A.L., Gorsuch, R., Yarka, C., Li, J., Bartlett, M., Pfister, D., Hyde, D.R., and Li, L. (2014). Transcription of the SCL/TAL1 interrupting Locus (Stil) is required for cell proliferation in adult Zebrafish Retinas. J. Biol. Chem. *289*, 6934–6940.

Takke, C., Dornseifer, P., v Weizsäcker, E., and Campos-Ortega, J.A. (1999). her4, a zebrafish homologue of the Drosophila neurogenic gene E(spl), is a target of NOTCH signalling. Development *126*, 1811–1821.

Teslaa, J.J., Keller, A.N., Nyholm, M.K., and Grinblat, Y. (2013). Zebrafish Zic2a and Zic2b regulate neural crest and craniofacial development. Dev. Biol. *380*, 73–86.

Thomas, J.L., Morgan, G.W., Dolinski, K.M., and Thummel, R. (2018). Characterization of the pleiotropic roles of Sonic Hedgehog during retinal regeneration in adult zebrafish. Exp. Eye Res. *166*, 106–115.

Todd, L., and Fischer, A.J. (2015). Hedgehog signaling stimulates the formation of proliferating Müller glia-derived progenitor cells in the chick retina. Development *142*, 2610–2622.

van den Hurk, M., Kenis, G., Bardy, C., van den Hove, D.L., Gage, F.H., Steinbusch, H.W., and Rutten, B.P. (2016). Transcriptional and epigenetic mechanisms of cellular reprogramming to induced pluripotency. Epigenomics *8*, 1131–1149.

Vokes, S.A., Ji, H., McCuine, S., Tenzen, T., Giles, S., Zhong, S., Longabaugh, W.J., Davidson, E.H., Wong, W.H., and McMahon, A.P. (2007). Genomic characterization of Gli-activator targets in sonic hedgehog-mediated neural patterning. Development *134*, 1977–1989.

Wan, J., and Goldman, D. (2016). Retina regeneration in zebrafish. Curr. Opin. Genet. Dev. *40*, 41–47.

Wan, J., Ramachandran, R., and Goldman, D. (2012). HB-EGF is necessary and sufficient for Müller glia dedifferentiation and retina regeneration. Dev. Cell *22*, 334–347.

Wilson, S.G., Wen, W., Pillai-Kastoori, L., and Morris, A.C. (2016). Tracking the fate of her4 expressing cells in the regenerating retina using her4:Kaede zebrafish. Exp. Eye Res. *145*, 75–87.

Yoshinari, N., Ishida, T., Kudo, A., and Kawakami, A. (2009). Gene expression and functional analysis of zebrafish larval fin fold regeneration. Dev. Biol. *325*, 71–81.

Zhang, T., Liu, W.D., Saunee, N.A., Breslin, M.B., and Lan, M.S. (2009). Zinc finger transcription factor INSM1 interrupts cyclin D1 and CDK4 binding and induces cell cycle arrest. J. Biol. Chem. *284*, 5574–5581.

Cell Reports, Volume *23*

Supplemental Information

let-7 MicroRNA-Mediated Regulation of Shh Signaling and the Gene Regulatory Network Is Essential for Retina Regeneration

Simran Kaur, Shivangi Gupta, Mansi Chaudhary, Mohammad Anwar Khursheed, Soumitra Mitra, Akshai Janardhana Kurup, and Rajesh Ramachandran

Experimental Procedures

Animals, fin cut, retinal injury and drugs.

Zebrafish were maintained at 26-28 °C on a 14 h:10 h light/dark cycle for all experiments unless specified. The *1016 tuba1a:*GFP transgenic fish used in this study have been characterized previously(Fausett and Goldman, 2006). Tricaine methanesulfonate is used as anesthetic. Fish embryos for all assays were obtained by natural breeding in laboratory. The Shh signaling inhibitor, cyclopamine; Mmp blockers, Salvianolic acid B and SB-3CT; protein transport inhibitor, Brefeldin A; Notch signaling blocker, *N*-[*N*-(3,5-difluorophenylacetyl)-L-alanyl]- *S*-phenylglycine *t*-butyl ester (DAPT), were made to a stock of 1mM, in DMSO for all experiments (all drugs were from Sigma-Aldrich). Drugs were delivered either through dipping or administration to the eye using a Hamilton syringe equipped with a 30-gauge needle. Retinal injury performed were previously described(Fausett and Goldman, 2006). All experiments were done to a minimum of six times for consistency and s.d.

C57BL /6 mice were used in this study. They were maintained at a cycle of 12 h light and 12 h dark cycle with continuous food and water accessibility. Animals were anaesthetized using isoflurane and eyes were injured or injected with a 30-gauge needle. Before harvesting the eyes animals were exposed to CO2 for euthanasia. The animal ethical committee at IISER Mohali approved these experiments.

RNAseq and analysis.

RNA was obtained from total retina from uninjured (control), 12 hours post injury and 4dpi, as previously described(Ramachandran et al., 2011), with or without cyclopamine treatment. The RNAseq was performed as shown previously(Brooks et al., 2012). The post sequence analysis were performed using TopHat and Cufflinks as reported earlier(Trapnell et al., 2012). The Supplementary Table S1 was created using a code developed in Python and the RNAseq data with special reference to transcription factors, obtained from the database AnimalTFDB2.0(Zhang et al., 2015), were analysed using it. The Venn diagrams in Figures S1J and S1K are created using FunRich (Functional Enrichment Analysis Tool; version 3.0) software(Pathan et al., 2015). The RNAseq data is deposited in repository at GEO Submission with ID of GSE102063.

Primers and plasmid construction

All primers are listed in Supplementary Table S5. The promoter of *her4.1* was amplified from zebrafish genomic DNA using primer pairs Xho-*her4.1*pro-F and Bam-*her4.1* pro-R (~4 kb). The digested PCR amplicon was cloned into a pEL luciferase expression vector to create *her4.1:*GFP-*luciferase* constructs. The *ascl1a:*GFP-*luciferase*, *lin28a:*GFP-*luciferase*, *insm1a:*GFP-*luciferase* constructs were described previously(Ramachandran et al., 2010a; Ramachandran et al., 2012).

Genes like *ascl1a*, *insm1a*, *lin28a*, and *nicd* were cloned from complementary DNA amplified from zebrafish retina RNA at 4 dpi using primer pairs Bam-Ascl1a FL-F and Xho-Ascl1a FL-R (~0.6 kb); Bam-*insm1a*-F and Xho-*insm1a*-R (~1.1kb); Bam-*lin28a* FL-F and Xho-*lin28a* FL-R (~0.6 kb). Post-digested PCR amplicons were cloned into their respective enzyme sites in pCS2^{+} plasmid to obtain *cmv:ascl1a*, *cmv:insm1a* and *cmv:lin28a.* The *nicd* mRNA was prepared from PCR amplification using primer pairs T7-HSP M-F and Sv40-R (~2kb) from a clone of *nicd* driven by Hsp70 promoter, which in turn was made in pTAL plasmid vector by digesting an amplicon of *nicd* obtained using PCR primers Hind2X-flag-*nicd*-F and MluI NICD-R.
Micro-RNA response elements (MRE) sequences of *shhb*, *ptch1* and *smo* were cloned in pEGFP-C1 vector using BamHI and MfeI restriction sites, and *shha* and *zic2b* were cloned using BamHI and XhoI restriction sites. Site directed mutagenesis of various constructs were performed as described previously(Ramachandran et al., 2012).

For the confirmation of MO activity, an adaptor having respective MO targeted region for *gli1*, *gli2a*, *gli3*, *mmp9* or *ptch1* was cloned in pEGFP-N1 in XhoI and BamHI restriction sites, and *ptch2* was cloned in XhoI and AgeI site, which append in-frame to GFP reporter. The plasmid with and without respective MOs was injected to observe the absence or presence of GFP fluorescence under fluorescence microscope.

Total RNA isolation, RT-PCR and qPCR analysis.

Total RNA was isolated from dark-adapted zebrafish retinae of control, injured and drug treated/MO electroporated group using TRIzol (Invitrogen). A combination of oligo-dT and random hexamers were used to reverse transcribe approximately 5 μg of RNA using Superscript II reverse transcriptase

(Invitrogen) to generate cDNA. PCR reactions used Taq or Phusion (New England Biolabs) thermo polymerase and gene-specific primers (Supplementary Table S5) with previously described cycling conditions(Ramachandran et al., 2010a). Quantitative PCR (qPCR) was carried out in triplicate with KOD SYBR qPCR mix (Genetix, QKD-201) on a real-time PCR detection system (Eppendorf MasterCycler RealPlex4). The relative expression of mRNAs in control and injured retinae was deciphered using the ΔΔCt method and normalized to ribosomal protein *l*-24 or β-actin mRNA levels.

mRNA synthesis, embryo micro-injection and ChIP assay.
Gene clone of *nicd* cDNA in pCS2$^+$ plasmid was linearized and capped mRNAs were synthesized using the mMESSAGE mMACHINE (Ambion) *in vitro* transcription system. For luciferase assay experiments, single-cell zebrafish embryos were injected with a total volume of ~1nl solution containing 0.02 pg of *Renilla* luciferase mRNA (normalization), 5 pg of *promoter:*GFP-*luciferase* vector and 0-6 pg of *nicd* mRNA or 0.1 to 0.5mM *shha*/*sufu* MOs. To assure consistency of results, a master mix was made for daily injections and ~300 embryos were injected at single cell stage. 24 hours later, embryos were divided into 3 groups (~ 70 embryos/group) and lysed for dual luciferase reporter assays (Promega, catalogue number E1910).

Chromatin immunoprecipitation (ChIP) assays were done in adult retina at 4dpi using ~50 adult retinae after dark adaptation. Chromatin was isolated by sonication as described previously(Lindeman et al., 2009). The chromatin after sonication was distributed into three equal aliquots; two were probed with an anti-zebrafish Gli1, Gli3, Shha and Ascl1a antibodies (described below) and the third served as a control. Primers used for ChIP assays are described in Supplementary Table S5.

Morpholino (MO) electroporation.
MOs tagged with lissamine (Gene Tools) of approximately 0.5 µl (0.5 to 1.0 mM) were injected at the time of injury using a Hamilton syringe of 2 µl volume capacity. MO delivery to cells was accomplished by electroporation as previously described (Fausett et al., 2008). The control and *ascl1a* targeting MOs have been previously described (Ramachandran et al., 2012). Morpholinos targeting *shha*, *shhb*, *sufu*, *gli1*, *gli2a*, *gli3*, *patched1*, *patched2*, *mmp9* and *zic2b* are: *shha*(1)-5'-GCACTCTCGTCAAAAGCCGCATTTT-3'; *shha*(2)-5'-CACGCTGAAT CTCGCTGCGGTGTTC-3'; *shhb*-5'-TCAGATGCAGCCTTACGTCCATGAC-3'; *patched1*-5'-AGGAGACATTAACAGCCGAGGCCAT-3';*patched2*-5'-CCGGGTCT CTGGGATCCGAGGCCAT-3'; *gli1*-5'-CTCCATGATGAGACTTCTTGGATGA-3'; *gli2a*-5'-GGGTTCCATGACAACTGGGCATTCC-3'; *gli3*-5'-GTTCCATGACA ACTGGGCATTCCTC-3'; *sufu*-5'-ACGCCAGGACTCCAAGTCTCATTTT-3'; *mmp9*-5'-GCTGCATATCCACTGGCATCGAGAC-3'; *zic2b*(1)-5'-GGTGGCCGGC GTCCAGTAACATCAC-3'; *zic2b*(2)-5'-CACGATTATTGACCAAAGAATGCGT-3'

Cell culture and transfection and western blotting.
The HEK293T cells were grown in a 90 mm petriplate before seeding into 24-well plate at approximately 40% confluence and grown in Dulbecco's Modified Eagle Medium (DMEM), 10% (v/v) fetal bovine serum, with antibiotics and antimycotics in a 37 °C incubator with 5% CO2. Cells were then transfected after 24 h from time of plating. To examine involvement of *let-7* microRNA in regulation of gene expression, cells were transfected with 50 ng of pEGFP-C1 vector harboring GFP-reporter tagged to *shha*, *shhb*, *smo*, *ptch1* or *zic2b* cDNA, along with 0, 50, 200 or 500 ng of the *ubC:let-7a/let-7f* vector and 50 ng of the *β-actin2:mCherry* normalization vector. 48 h post-transfection, cells were harvested and protein expression was assayed by western blotting.
For *in vivo* experiments, Western blotting was performed using whole retina tissue using 4 retinae per experimental sample, lysed in Laemmli buffer, size fractioned in 12% acrylamide gel with SDS at denaturing conditions before transferring on to Immun-Blot PVDF membrane (Biorad Catalogue number 162-0177), followed by probing with specific primary antibodies and HRP conjugated secondary for chemiluminescence assay using Clarity Western ECL (Biorad Catalogue number 170-5061).

BrdU llabeling, Retina tissue preparation for mRNA *in situ* hybridization, immunofluorescence microscopy, and TUNEL Assay
BrdU labeling was performed by single i.p. injection of 20 µl of BrdU (20 mM) 3 h before euthanasia and retina dissection, unless mentioned specifically. Some animals required for long-term cell tracing experiments received more BrdU injections over multiple days. Fish were given higher dose of tricaine methane sulphonate and eyes were dissected, lens removed, fixed in 4% paraformaldehyde and

sectioned as described previously(Fausett and Goldman, 2006). The mRNA *in situ* hybridization (ISH) was performed on retinal sections with fluorescein or digoxigenin-labelled complementary RNA probes (FL/DIG RNA llabeling kit, Roche Diagnostics)(Barthel and Raymond, 2000) The micro RNA *let-7* ISH were done as described previously (Ramachandran et al., 2010a). Fluorescence ISH was performed according to the manufacturer's directions (Thermo Fisher Scientific, catalogue numbers T20917, B40955, B40953). Sense probes were used in every ISH separately as control, to assess the potential of background signal. Immunofluorescence microscopy protocols and antibodies were previously described(Ramachandran et al., 2010b; Ramachandran et al., 2012). Immunofluorescence microscopy was performed rabbit polyclonal antibody against human ASCL1/MASH1 (Abcam, catalogue number ab74065); Rat monoclonal antibody against BrdU (Abcam, catalogue number ab6326); Mouse monoclonal antibody against human proliferating cell nuclear antigen, PCNA (Santa Cruz, catalogue number sc-25280); Rabbit polyclonal antibody against zebrafish Gli1 (Anaspec, catalogue number AS-55627); Rabbit polyclonal antibody against zebrafish Gli3 (Anaspec, catalogue number AS-55630); Rabbit polyclonal antibody against zebrafish Patched 1 (Anaspec, catalogue number AS-55641); Rabbit polyclonal antibody against zebrafish Shha (Anaspec, catalogue number AS-55574s); Rabbit polyclonal antibody against zebrafish Smo (Anaspec, catalogue number AS-55647); Mouse polyclonal antibody against GFP (Abcam, catalogue number ab-38689); Rabbit polyclonal antibody against zebrafish mCherry (Abcam, catalogue number ab-183628); Mouse monoclonal antibody against Actin (Santa Cruz, catalogue number sc-81178); Rabbit polyclonal antibody against mouse glutamine synthetase (Abcam, catalogue number ab93439); Mouse polyclonal antibody against HuD (Santa Cruz, catalogue number sc-48421); Goat polyclonal antibody against protein kinase C β1 (PKCβ1) (Santa Cruz, catalogue number sc-209-G) at 1:500 dilution. Before BrdU immunofluorescence microscopy, retinal sections were treated with 2 N HCl at 37 °C for 20 min, equilibrated with 100mM sodium borate (pH 8.5) for 10 min, twice and then processed using standard procedures(Senut et al., 2004). BrdU labelled MGPC lineage-tracing experiments were done in retinal sections from single eye sections of 8 μm thickness, distributed across five slides. Individual slide was first processed for immunofluorescence based detection of specific antigen or mRNA and then BrdU or PCNA staining was performed as mentioned above using respective antibodies(Powell et al., 2012; Ramachandran et al., 2012). The total number of $BrdU^+$ cells and the number of co-labelled $BrdU^+$ cells that also stained with a specific ISH probe and subsequent enzymatic reaction, were quantified on each slide. TUNEL assay was performed on retinal sections using In Situ Cell death Detection Fluorescein kit (Roche, Ref no:11684795910) as per manufacturer recommended protocol.

Fluorescence and confocal microscopy, cell counting and statistical analysis.
After the completion of staining experiments, the slides were examined with a Nikon N*i*-E fluorescence microscope equipped with fluorescence optics and Nikon A1 confocal imaging system. The $PCNA^+$ and $BrdU^+$ cells were counted by observation of their fluorescence in retinal sections, ISH^+ cells through bright field, visualized in the same microscope and quantified. Every sections of the stained retina were mounted, observed and analysed, and at least three retinae from separate fish were used. Observed data were analysed for statistical significance by comparisons done using a two-tailed unpaired Student's *t*-test to analyse data from all experiments. For all other comparisons, analysis of variance (ANOVA) was performed and subsequently a Bonferroni/Dunn *post hoc t*-test was done using Stat View software. Error bars represent s.d in all histograms.

Fluorescence based cell sorting.
RNA was obtained from FACS purified MG and MG-derived progenitors at 4 dpi as previously described (Ramachandran et al., 2011, 2012). Briefly uninjured and injured retinas were isolated from *1016 tuba1a:*GFP transgenic fish. GFP+ MGPCs from *1016 tuba1a:*GFP retinas at 4 dpi were isolated by treating retinas with hyaluronidase and trypsin and then sorted on a BD FACS Aria Fusion high speed cell sorter. Approximately 40 injured retinas from *1016 tuba1a:*GFP fish yielded 80,000 GFP positive and 170,000 GFP cells.

References
Barthel, L.K., and Raymond, P.A. (2000). In situ hybridization studies of retinal neurons. Methods Enzymol *316*, 579-590.
Brooks, M.J., Rajasimha, H.K., and Swaroop, A. (2012). Retinal transcriptome profiling by directional next-generation sequencing using 100 ng of total RNA. Methods Mol Biol *884*, 319-334.

Fausett, B.V., and Goldman, D. (2006). A role for alpha1 tubulin-expressing Muller glia in regeneration of the injured zebrafish retina. J Neurosci *26*, 6303-6313.
Fausett, B.V., Gumerson, J.D., and Goldman, D. (2008). The proneural basic helix-loop-helix gene ascl1a is required for retina regeneration. J Neurosci *28*, 1109-1117.
Lindeman, L.C., Vogt-Kielland, L.T., Alestrom, P., and Collas, P. (2009). Fish'n ChIPs: chromatin immunoprecipitation in the zebrafish embryo. Methods Mol Biol *567*, 75-86.
Pathan, M., Keerthikumar, S., Ang, C.S., Gangoda, L., Quek, C.Y., Williamson, N.A., Mouradov, D., Sieber, O.M., Simpson, R.J., Salim, A., *et al.* (2015). FunRich: An open access standalone functional enrichment and interaction network analysis tool. Proteomics *15*, 2597-2601.
Powell, C., Elsaeidi, F., and Goldman, D. (2012). Injury-dependent Muller glia and ganglion cell reprogramming during tissue regeneration requires Apobec2a and Apobec2b. J Neurosci *32*, 1096-1109.
Ramachandran, R., Fausett, B.V., and Goldman, D. (2010a). Ascl1a regulates Muller glia dedifferentiation and retinal regeneration through a Lin-28-dependent, let-7 microRNA signalling pathway. Nat Cell Biol *12*, 1101-1107.
Ramachandran, R., Reifler, A., Parent, J.M., and Goldman, D. (2010b). Conditional gene expression and lineage tracing of tuba1a expressing cells during zebrafish development and retina regeneration. J Comp Neurol *518*, 4196-4212.
Ramachandran, R., Zhao, X.F., and Goldman, D. (2011). Ascl1a/Dkk/beta-catenin signaling pathway is necessary and glycogen synthase kinase-3beta inhibition is sufficient for zebrafish retina regeneration. Proc Natl Acad Sci U S A *108*, 15858-15863.
Ramachandran, R., Zhao, X.F., and Goldman, D. (2012). Insm1a-mediated gene repression is essential for the formation and differentiation of Muller glia-derived progenitors in the injured retina. Nat Cell Biol *14*, 1013-1023.
Senut, M.C., Gulati-Leekha, A., and Goldman, D. (2004). An element in the alpha1-tubulin promoter is necessary for retinal expression during optic nerve regeneration but not after eye injury in the adult zebrafish. J Neurosci *24*, 7663-7673.
Trapnell, C., Roberts, A., Goff, L., Pertea, G., Kim, D., Kelley, D.R., Pimentel, H., Salzberg, S.L., Rinn, J.L., and Pachter, L. (2012). Differential gene and transcript expression analysis of RNA-seq experiments with TopHat and Cufflinks. Nat Protoc *7*, 562-578.
Zhang, H.M., Liu, T., Liu, C.J., Song, S., Zhang, X., Liu, W., Jia, H., Xue, Y., and Guo, A.Y. (2015). AnimalTFDB 2.0: a resource for expression, prediction and functional study of animal transcription factors. Nucleic Acids Res *43*, D76-81.

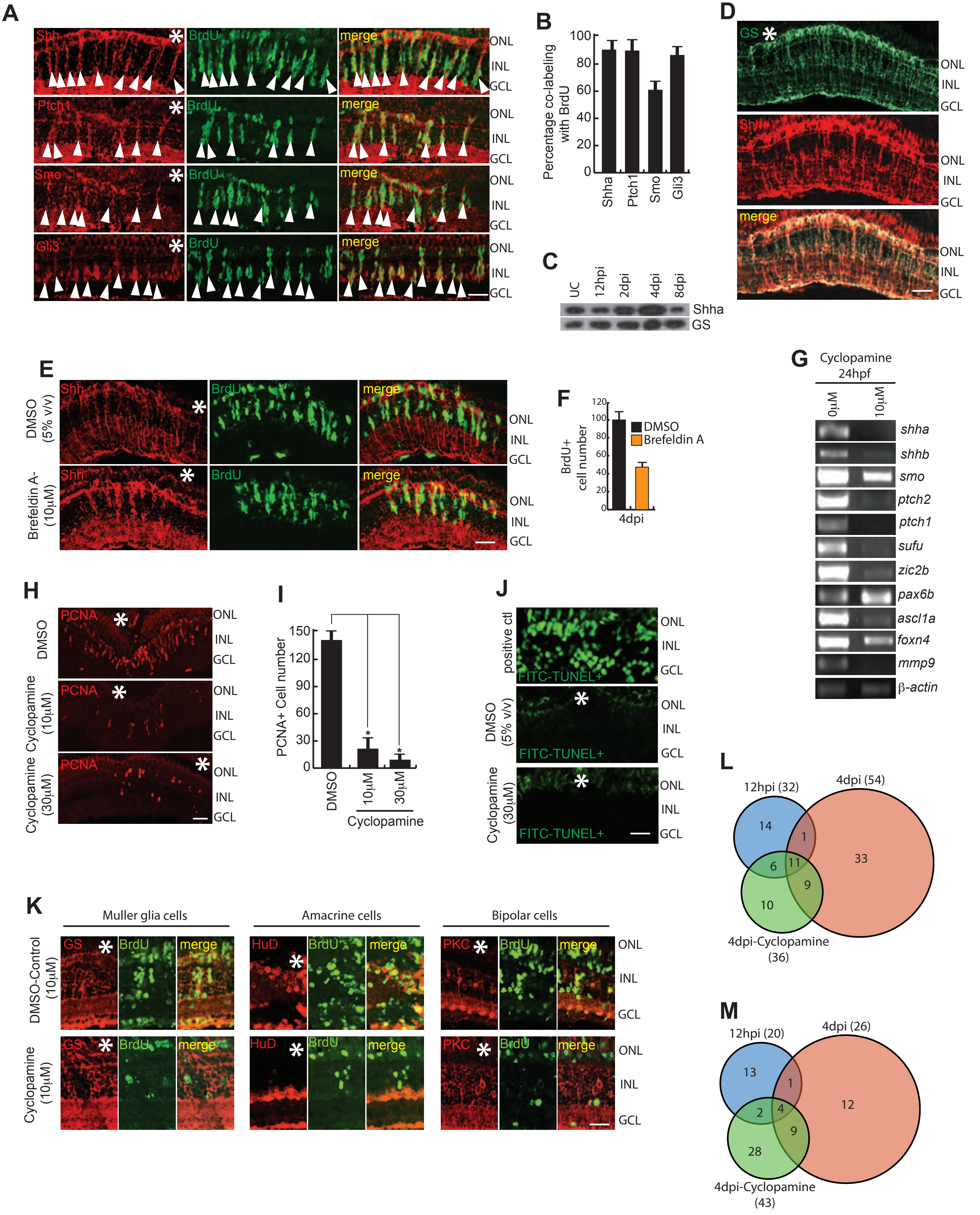

A
Shh
BrdU
merge
Ptch1
Smo
Gli3
ONL
INL
GCL
B
Percentage co-labeling with BrdU
100
80
60
40
20
0
Shha
Ptch1
Smo
Gli3
C
UC
12hpi
2dpi
4dpi
8dpi
Shha
GS
D
GS
Shh
merge
E
DMSO (5% v/v)
Brefeldin A- (10μM)
F
BrdU+ cell number
DMSO
Brefeldin A
4dpi
G
Cyclopamine 24hpf
0μM
10μM
shha
shhb
smo
ptch2
ptch1
sufu
zic2b
pax6b
ascl1a
foxn4
mmp9
β-actin
H
PCNA
DMSO
Cyclopamine (10μM)
Cyclopamine (30μM)
I
PCNA+ Cell number
150
120
90
60
30
0
DMSO
10μM
30μM
Cyclopamine
J
positive ctl
FITC-TUNEL+
DMSO (5% v/v)
Cyclopamine (30μM)
K
Muller glia cells
Amacrine cells
Bipolar cells
DMSO-Control (10μM)
Cyclopamine (10μM)
HuD
PKC
L
12hpi (32)
4dpi (54)
14
1
6
11
33
9
10
4dpi-Cyclopamine (36)
M
12hpi (20)
4dpi (26)
13
1
2
4
12
9
28
4dpi-Cyclopamine (43)

Supplementary Figure S1. Shh signaling mediated gene expression and lineage tracing of MGPCs in cyclopamine treatment.

(**A,B**) High magnification immunofluorescence microscopy (IF) images of 4dpi retinal sections showed co-localization of Shh signaling components with $BrdU^+$ MGPCs (**A**)**,** which is quantified in (**B**). (**C**) Western blotting assay showed regulation of Shha protein following injury at various time points. (**D**) IF microscopy images of wild type 4dpi retinal sections revealed significant co-localization of Shha with Glutamine Synthetase (GS), which marks all the Muller glia, at the injury site. (**E**) IF microscopy of Shha in $BrdU^+$ MGPCs, in 4dpi retina, with Brefeldin A treatment, which is a protein transport inhibitor. (**F**) $BrdU^+$ cells were quantified in Brefeldin A treatment. (**G**) RT-PCR analysis of indicated genes mRNA levels in DMSO and cyclopamine treated 24hpf embryos. (**H,I**) IF microscopy images showed a cyclopamine dose-dependent decline in $PCNA^+$ MGPCs wild-type (**H**), retinae at 4 dpi, which is quantified in (**I**) (**J**) Terminal deoxynucleotidyl transferase (TdT) dUTP Nick-End Labeling (TUNEL) assay done on DNase treated positive control, 4dpi and cyclopamine treated 4dpi retinal sections, showed presence of $TUNEL^+$ cells only in positive control. (**K**) Cell-fate tracing experiment was done by injuring the fish followed by treating them with 5% (v/v) DMSO or 10μM cyclopamine for first four days followed by an i.p injection of BrdU and then were transferred to water for next 26 days until euthanasia. IF microscopy images of 30dpi retinal sections revealed co-localization of GS, which marks Muller glia, HuD which marks amacrine cells and PKC which marks horizontal cells, with $BrdU^+$ cells in DMSO treated retina but not in cyclopamine treated retina. (**L,M**) Whole retina RNAseq analysis of DNA binding proteins and transcription factors at 12hpi, 4dpi and 4dpi cyclopamine treatment were compared with uninjured retina showed upregulated genes (**L**), and downregulated genes (**M**). Scale bars, 10 μm (**A,E,H,J** and **K**) and 20 μm (**D**). Error bars are SD.

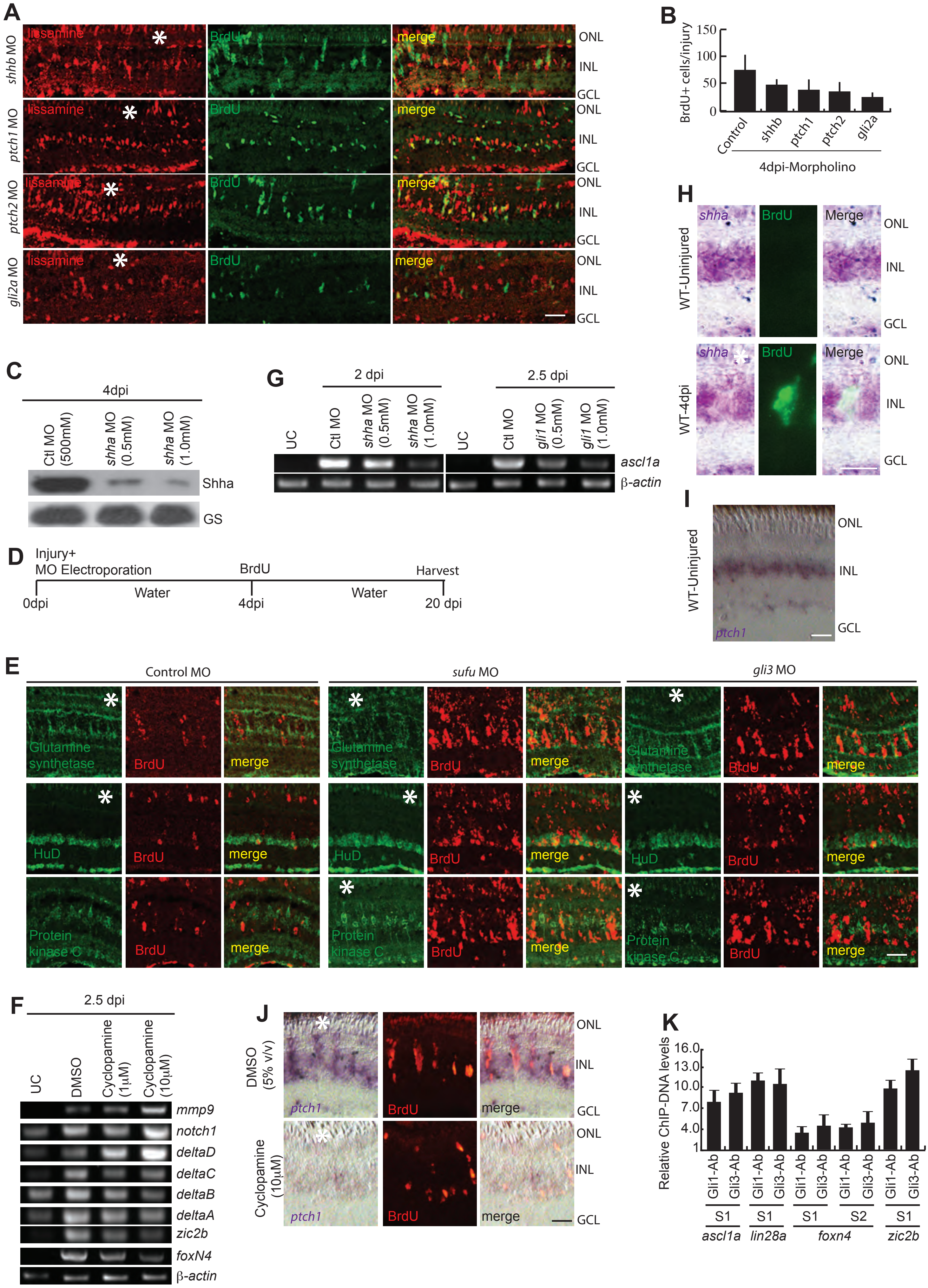
A
shhb MO
ptch1 MO
ptch2 MO
gli2a MO
lissamine
BrdU
merge
ONL
INL
GCL
B
BrdU+ cells/injury
150
100
50
0
Control
shhb
ptch1
ptch2
gli2a
4dpi-Morpholino
C
4dpi
Ctl MO (500mM)
shha MO (0.5mM)
shha MO (1.0mM)
Shha
GS
D
Injury+
MO Electroporation
BrdU
Harvest
Water
Water
0dpi
4dpi
20 dpi
G
2 dpi
2.5 dpi
UC
Ctl MO
shha MO (0.5mM)
shha MO (1.0mM)
UC
Ctl MO
gli1 MO (0.5mM)
gli1 MO (1.0mM)
ascl1a
β-actin
H
WT-Uninjured
WT-4dpi
shha
BrdU
Merge
ONL
INL
GCL
I
WT-Uninjured
ptch1
ONL
INL
GCL
E
Control MO
sufu MO
gli3 MO
Glutamine synthetase
HuD
Protein kinase C
BrdU
merge
F
2.5 dpi
UC
DMSO
Cyclopamine (1μM)
Cyclopamine (10μM)
mmp9
notch1
deltaD
deltaC
deltaB
deltaA
zic2b
foxN4
β-actin
J
DMSO (5% v/v)
Cyclopamine (10μM)
ptch1
BrdU
merge
ONL
INL
GCL
K
Relative ChIP-DNA levels
16.0
13.0
10.0
7.0
4.0
1
Gli1-Ab
Gli3-Ab
S1
S1
S1
S2
S1
ascl1a
lin28a
foxn4
zic2b

Supplementary Figure S2: Shh signaling component genes' knockdowns and lineage tracing of MGPCs in enhanced Shh signaling.

(**A,B**) IF microscopy images of 4dpi retina revealed decline in proliferation marked by reduction in $BrdU^+$ cells in *shhb*, *ptch1, ptch2* and *gli2a* knockdowns (**A**), which was quantified in (**B**). (**C**) Western blotting assay indicating *shha* knockdown caused downregulation in the expression of Shha protein. (**D,E**) The schematic of lineage tracing experiment, wherein control, *sufu* or *gli3* MOs were injected and electroporated while injuring the retina, and an i.p. injection of BrdU was given on 4dpi and eyes were harvested at 20dpi (**D**), the increased number of $BrdU^+$ cells could make retinal cell types (**E**). (**F**) RT-PCR analysis of indicated genes' mRNA levels in DMSO and cyclopamine treated 2.5dpi retina. (**G**) RT-PCR analysis of *ascl1a* mRNA levels in uninjured retina, control knockdown and *gli1* knockdown in 2.5dpi retina (**H**) Bright field (BF) and IF microscopy revealed the expression of *shha* mRNA and BrdU in uninjured and 4dpi retina. (**I,J**) ISH and IF of *ptch1* and BrdU respectively, in uninjured (**I**), 4dpi and 4dpi with cyclopamine treatment (**J**). (**K**) Relative abundance of ChIP DNA fragments obtained from Gli1 and Gli3 antibodies from various gene promoters, assayed by qPCR, which are normalized to control uninjured retina. Scale bars, 10 µm (**A,E,H,I,J**). Error bars are SD.

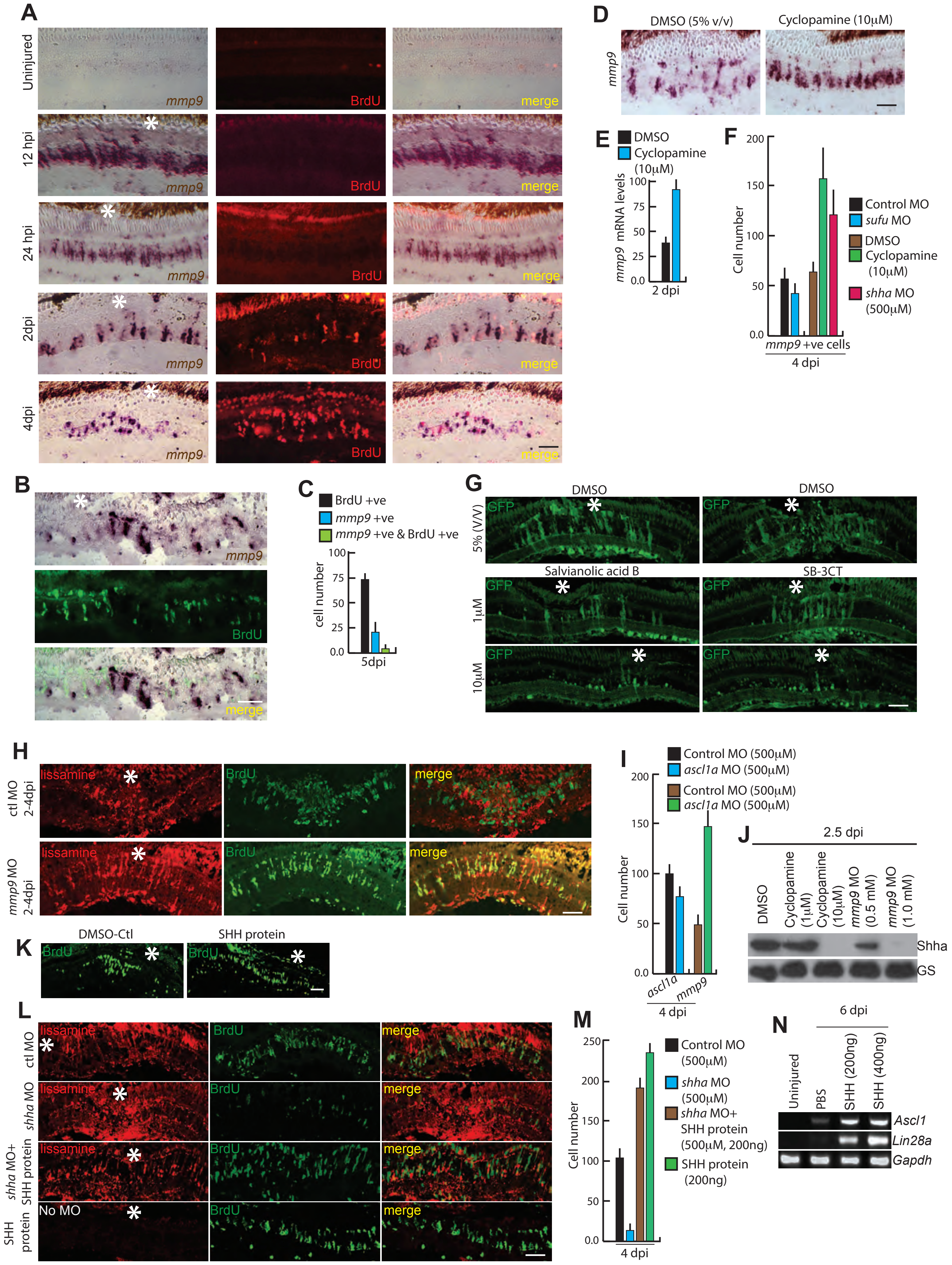
A
Uninjured
12 hpi
24 hpi
2dpi
4dpi
mmp9
BrdU
merge
D
DMSO (5% v/v)
Cyclopamine (10μM)
E
DMSO
Cyclopamine (10μM)
mmp9 mRNA levels
2 dpi
F
Control MO
sufu MO
DMSO
Cyclopamine (10μM)
shha MO (500μM)
Cell number
mmp9 +ve cells
4 dpi
B
C
BrdU +ve
mmp9 +ve
mmp9 +ve & BrdU +ve
cell number
5dpi
G
DMSO
5% (V/V)
Salvianolic acid B
SB-3CT
1μM
10μM
GFP
H
lissamine
ctl MO 2-4dpi
mmp9 MO 2-4dpi
I
Control MO (500μM)
ascl1a MO (500μM)
Cell number
ascl1a
mmp9
4 dpi
J
2.5 dpi
DMSO
Cyclopamine (1μM)
Cyclopamine (10μM)
mmp9 MO (0.5 mM)
mmp9 MO (1.0 mM)
Shha
GS
K
DMSO-Ctl
SHH protein
L
ctl MO
shha MO
shha MO+ SHH protein
SHH protein
No MO
M
Control MO (500μM)
shha MO (500μM)
shha MO+ SHH protein (500μM, 200ng)
SHH protein (200ng)
Cell number
4 dpi
N
6 dpi
Uninjured
PBS
SHH (200ng)
SHH (400ng)
Ascl1
Lin28a
Gapdh

Supplementary Figure S3: *mmp9* expression pattern and impact of SHH protein injection in regenerating retina.

(**A-C**) BF and IF microscopy images of *mmp9* and BrdU$^+$ cells at various time points post injury (**A**), and BrdU co-labeling with *mmp9* at 5dpi (**B**), quantified in (**C**). (**D,E**) ISH microscopy revealed increased *mmp9* expression in 4dpi retina with cyclopamine treatment (**D**), mRNA levels quantified in (**E**). (**F**) Quantification of *mmp9*$^+$ cells in 5% (v/v) DMSO control, 10μM cyclopamine treatment and *shha* or *sufu* knockdowns in 4dpi retina. (**G**) IF microscopy images show Salvianolic acid B and SB-3CT dependent decline in GFP$^+$ MGPCs in *1016 tuba:*GFP transgenic zebrafish at 4dpi. (**H**) MOs against control and *mmp9* were injected and electroporated at 2dpi, then an i.p. injection of BrdU was given on 4dpi, 3 hours before euthanasia, and no change in the number of BrdU$^+$ cells was found in both knockdowns. (**I**) Quantification of *ascl1a*$^+$ and *mmp9*$^+$ cells in control and *ascl1a* knockdown retina at 4dpi. (**J**) Western blotting assay indicating cyclopamine *or mmp9* knockdown in 2.5dpi retina caused decline in Shha expression levels. (**K**) Zone of BrdU$^+$ cells in the regenerating retina, increased upon injection of recombinant Shha protein (200ng) at 4dpi. (**L,M**) IF microscopy images revealed an increase in BrdU$^+$ cell number in combined injection of recombinant SHH protein and *shha* MO, and isolated injection of SHH protein (200ng) in 4dpi retina, whereas BrdU$^+$ cells declined in *shha* knockdown (**L**), which is quantified in (**M**), suggesting external SHH could impact retina regeneration even in absence of endogenous Shh protein. (**N**) RT-PCR analysis of *Ascl1* and *Lin28a* genes in 6dpi mouse retina exposed to recombinant SHH protein at the time of injury until harvest. Scale bars, 10 μm (**A,B,G,H,K,L**). Error bars are SD.

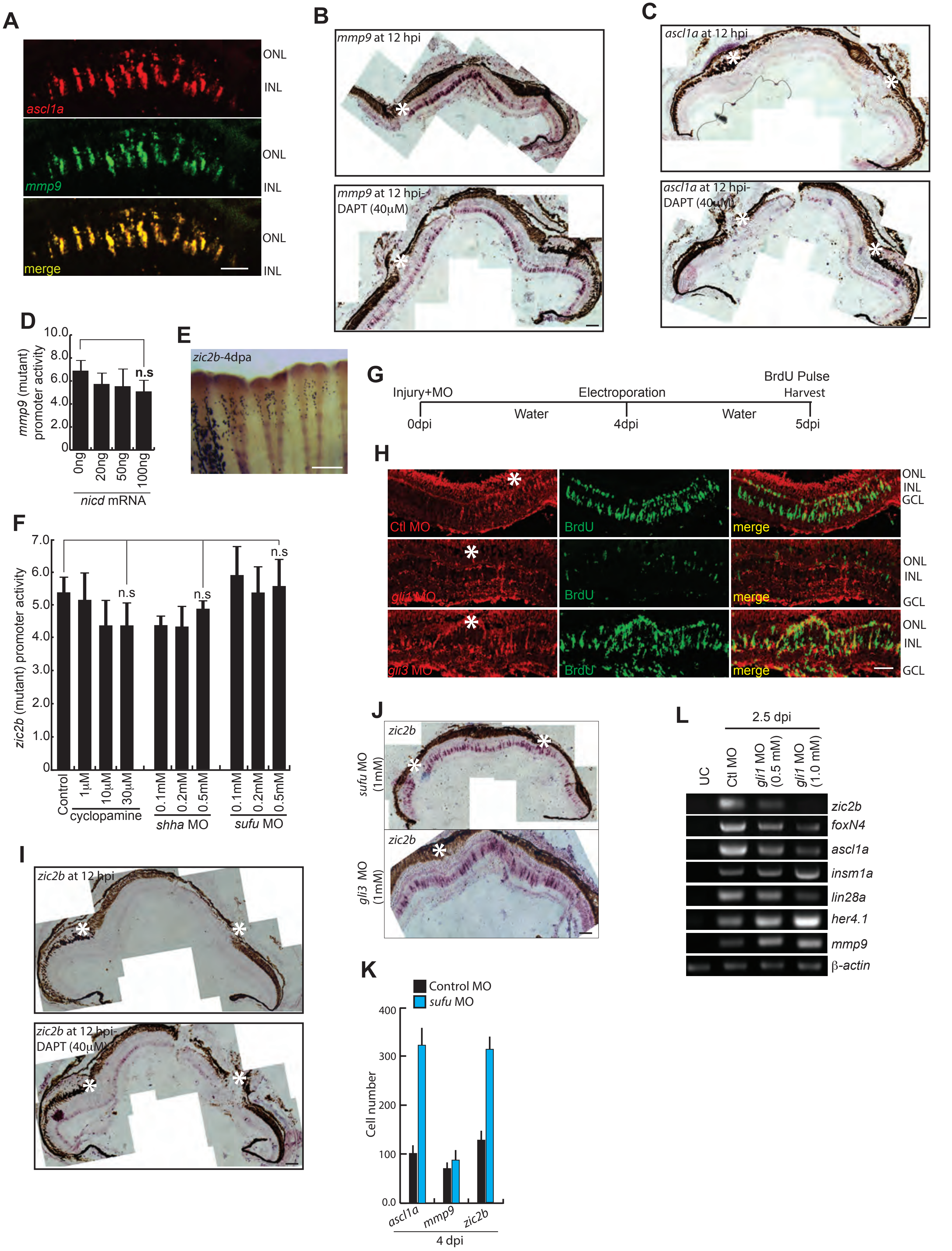
A
ascl1a
mmp9
merge
ONL
INL
B
mmp9 at 12 hpi
mmp9 at 12 hpi-DAPT (40μM)
C
ascl1a at 12 hpi
ascl1a at 12 hpi-DAPT (40μM)
D
mmp9 (mutant) promoter activity
n.s
0ng
20ng
50ng
100ng
nicd mRNA
E
zic2b-4dpa
F
zic2b (mutant) promoter activity
n.s
Control
1μM
10μM
30μM
cyclopamine
0.1mM
0.2mM
0.5mM
shha MO
sufu MO
G
Injury+MO
Electroporation
BrdU Pulse
Harvest
Water
0dpi
4dpi
5dpi
H
Ctl MO
gli1 MO
gli3 MO
BrdU
merge
GCL
I
zic2b at 12 hpi
zic2b at 12 hpi-DAPT (40μM)
J
zic2b
sufu MO (1mM)
gli3 MO (1mM)
K
Control MO
sufu MO
Cell number
ascl1a
mmp9
zic2b
4 dpi
L
2.5 dpi
UC
Ctl MO
gli1 MO (0.5 mM)
gli1 MO (1.0 mM)
foxN4
insm1a
lin28a
her4.1
β-actin

Supplementary Figure S4: Impact of DAPT treatment or *gli1*/*gli3* knockdowns in gene expression pattern and cell proliferation.
(**A**) FISH microscopy of *ascl1a* and *mmp9* in 4dpi retinal sections. (**B-C**) Low magnification BF microscopy images of mRNA *in situ* hybridization of *mmp9* (**B**) and *ascl1a* (**C**), in DAPT treated retina at 12hpi, showed an increase in its expression as compared with control. (**D**) Mutated Her/Hes binding sites abolished the impact of *nicd* over expression on *mmp9* promoter, in zebrafish embryo luciferase assay. (**E**) BF image of mRNA *in situ* hybridization of *zic2b* in 4 days post amputated zebrafish fin. (**F**) Luciferase assay showed that mutations to the Gli-BS abolished the impact of Shh signaling in *zic2b* promoter. (**G**) Schematic describing experimental regime of MO injection at the time of injury and electroporation at 4dpi, followed by an i.p. injection of BrdU at 5dpi before euthanasia. (**H**) IF microscopy images revealed decrease and an increase in $BrdU^+$ cells in *gli1 and gli3* knockdowns respectively. (**I**) Low magnification BF microscopy images of mRNA *in situ* hybridization of *zic2b* in DAPT treated retina, at 12hpi. (**J**) BF images of mRNA *in situ* hybridization of *zic2b* in *sufu* or *gli3* knockdown in 4dpi retina. (**K**) Quantification of *ascl1a*$^+$*, mmp9*$^+$ *and zic2b*$^+$ cells in control and *sufu* knockdown. (**L**) RT-PCR analysis of indicated genes' mRNA levels in uninjured retina, control and *gli1* knockdown retina in 2.5dpi. Scale bars, 10 μm (**A,B,C,H,I,J**) and 500 μm (**E**). Error bars are SD.

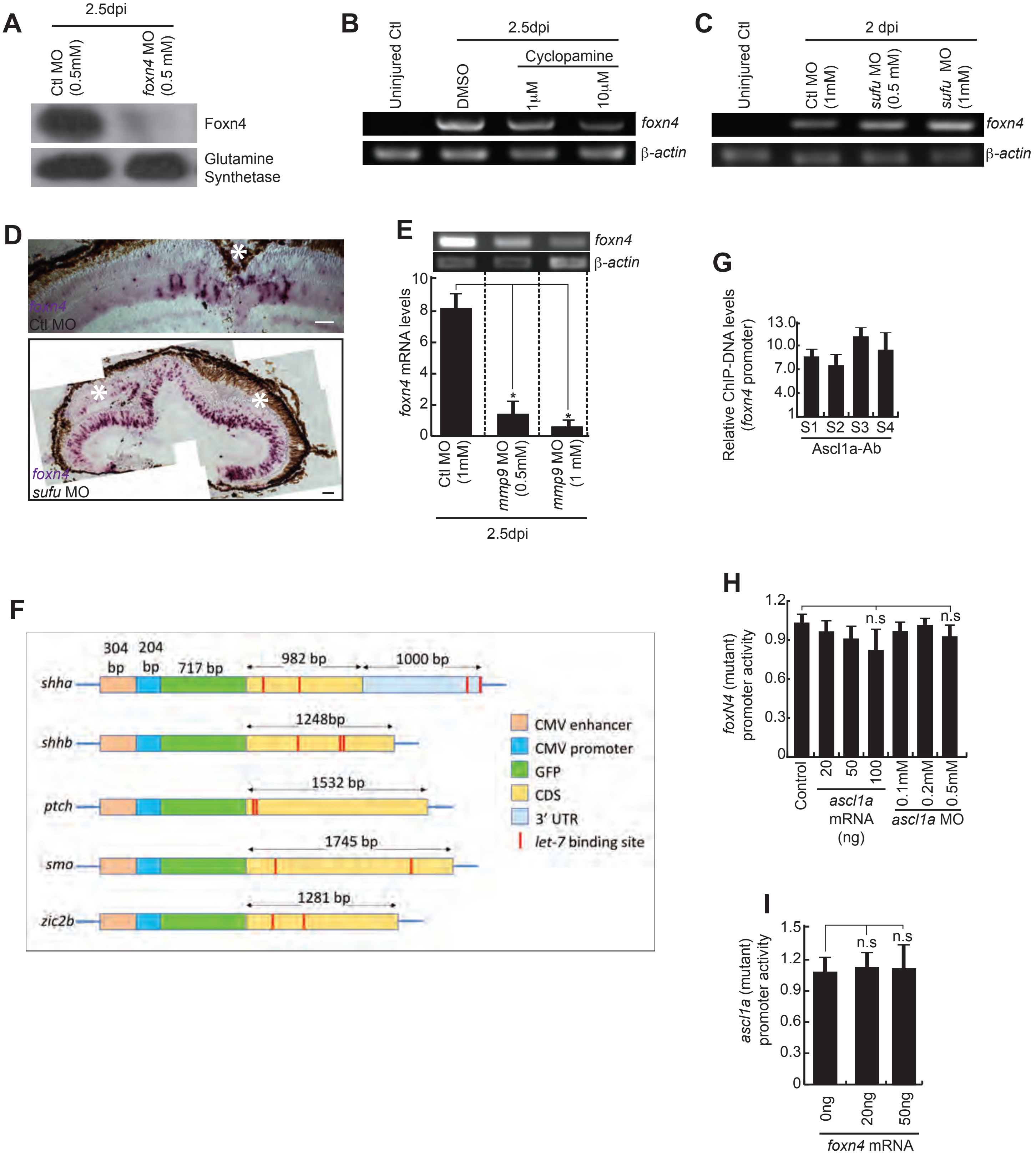

A
2.5dpi
Ctl MO (0.5mM)
foxn4 MO (0.5 mM)
Foxn4
Glutamine Synthetase
B
Uninjured Ctl
2.5dpi
DMSO
Cyclopamine
1μM
10μM
foxn4
β-actin
C
Uninjured Ctl
2 dpi
Ctl MO (1mM)
sufu MO (0.5 mM)
sufu MO (1mM)
foxn4
β-actin
D
Ctl MO
foxn4
sufu MO
E
foxn4
β-actin
foxn4 mRNA levels
Ctl MO (1mM)
mmp9 MO (0.5mM)
mmp9 MO (1 mM)
2.5dpi
G
Relative ChIP-DNA levels (foxn4 promoter)
S1 S2 S3 S4
Ascl1a-Ab
F
304 bp
204 bp
717 bp
982 bp
1000 bp
shha
shhb
1248bp
ptch
1532 bp
smo
1745 bp
zic2b
1281 bp
CMV enhancer
CMV promoter
GFP
CDS
3' UTR
let-7 binding site
H
foxN4 (mutant) promoter activity
n.s
n.s
Control
20
50
100
ascl1a mRNA (ng)
0.1mM
0.2mM
0.5mM
ascl1a MO
I
ascl1a (mutant) promoter activity
n.s
n.s
0ng
20ng
50ng
foxn4 mRNA

Supplementary Figure S5: Expression of *foxn4* in retina at various conditions

(**A**) Western blot analysis of Foxn4 in *foxn4*-MO electroporated retina, at 2.5dpi. (**B**) RT-PCR analysis of *foxn4* in uninjured control, 2.5dpi DMSO-treated, and 2.5dpi cyclopamine-treated whole retina. (**C**) RT-PCR analysis of *foxn4* from *sufu* MO-electroporated retina compared with control MO, at 2dpi. (**D**) BF microscopy images of *foxn4* mRNA ISH in retinal sections electroporated with control and *sufu* MOs at 4dpi. (**E**) RT-PCR (upper) and qPCR (lower) analysis of *foxn4* in control MO, and *mmp9* MO electroporated in 2.5dpi retina. $^{*}P<0.001$ in **E**, and error bars are SD. (**F**) Schematic representation of DNA constructs used in transfection experiments for examining the impact of *let-7* microRNA on various genes. (**G**)qPCR assay revealed the relative abundance of ChIP DNA fragments of *foxn4* promoter obtained by Ascl1a antibody which are normalized to control uninjured retina. (**H**) Luciferase assay revealed that mutated Ascl1a-BS on *foxn4* promoter had little effect on positive or negative regulation by *ascl1a* mRNA or MO respectively. (**I**) Luciferase assay revealed that mutated Foxn4-BS on *ascl1a* promoter had little effect on positive regulation by *foxn4* mRNA. Scale bars, 10 µm (**D**).

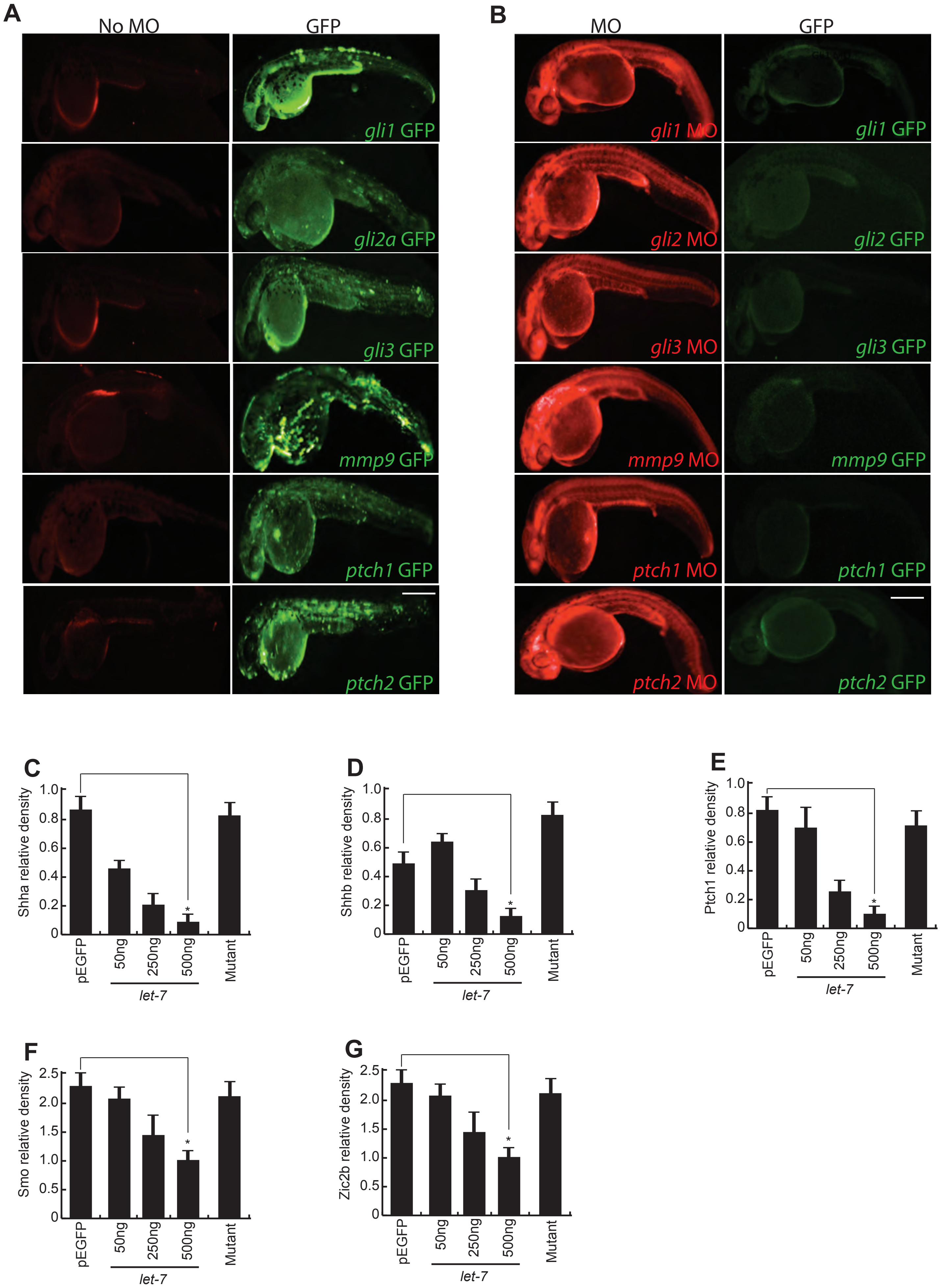
A
No MO
GFP
gli1 GFP
gli2a GFP
gli3 GFP
mmp9 GFP
ptch1 GFP
ptch2 GFP
B
MO
GFP
gli1 MO
gli1 GFP
gli2 MO
gli2 GFP
gli3 MO
gli3 GFP
mmp9 MO
mmp9 GFP
ptch1 MO
ptch1 GFP
ptch2 MO
ptch2 GFP
C
Shha relative density
D
Shhb relative density
E
Ptch1 relative density
F
Smo relative density
G
Zic2b relative density
pEGFP
50ng
250ng
500ng
Mutant
let-7

Supplementary Figure S6: MO assay in embryos. (**A,B**) The fusion mRNA, prepared by *in vitro* transcription using the clone containing GFP coding sequence in pCS2+ plasmid appended with the morpholino binding region of the respective genes, was injected alone (**A**), and along with morpholinos (**B**) in zebrafish embryos at single cell stage and imaged for GFP and lissamine fluorescence in a fluorescence microscope, at 24hpf. (**C-G**) Densitometry plots showing the expression of various GFP fusion proteins in *let-7* micro RNA dependent manner in HEK293T cells, normalized to transfection control mCherry. $^{*}P<0.0001$. Scale bars, 500 μm (**A,B**).

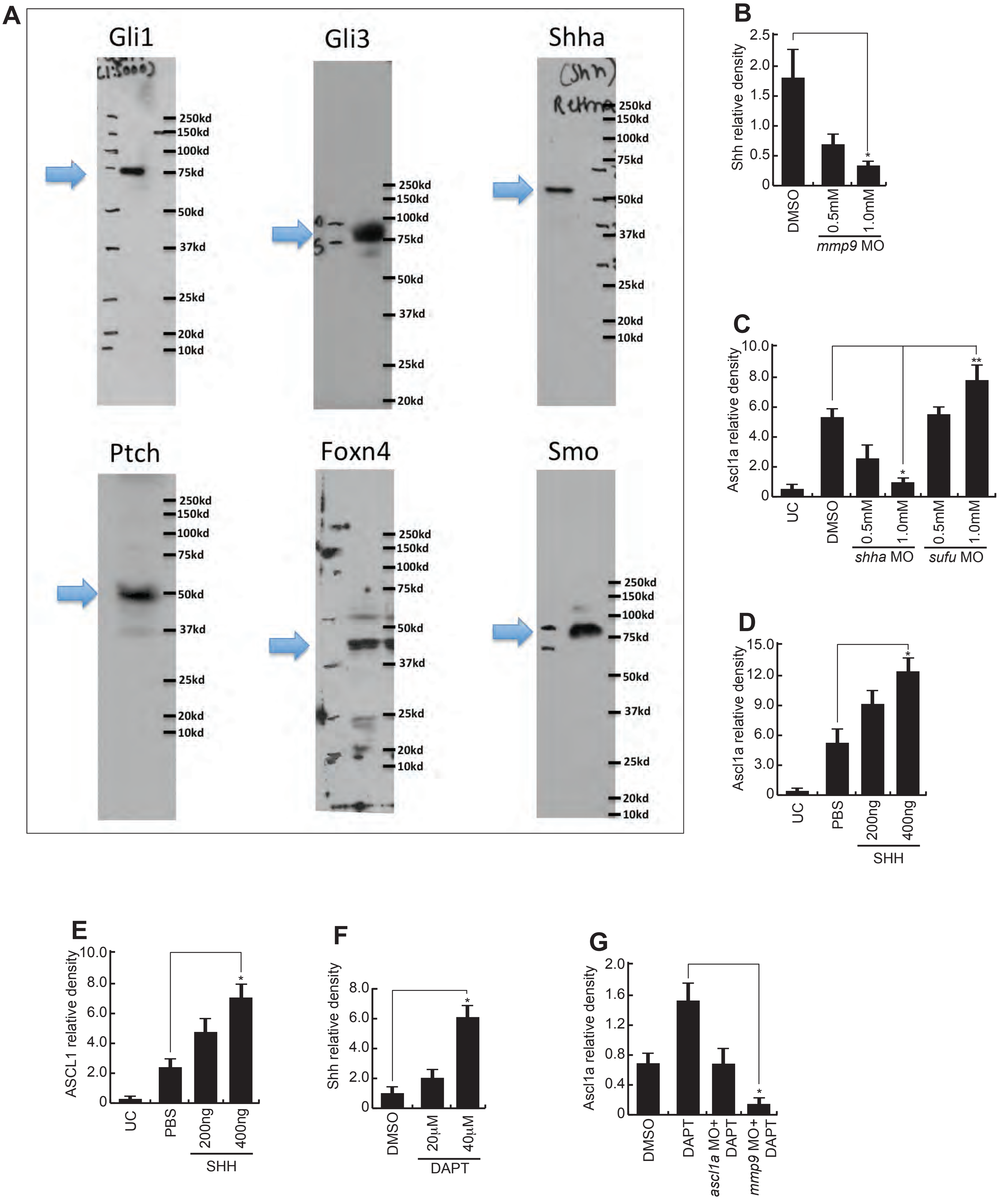
A
Gli1
Gli3
Shha
Ptch
Foxn4
Smo
250kd
150kd
100kd
75kd
50kd
37kd
25kd
20kd
10kd
B
Shh relative density
2.5
2.0
1.5
1.0
0.5
0
DMSO
0.5mM
1.0mM
mmp9 MO
C
Ascl1a relative density
10.0
8.0
6.0
4.0
2.0
0
UC
DMSO
0.5mM
1.0mM
shha MO
0.5mM
1.0mM
sufu MO
D
Ascl1a relative density
15.0
12.0
9.0
6.0
3.0
0
UC
PBS
200ng
400ng
SHH
E
ASCL1 relative density
10.0
8.0
6.0
4.0
2.0
0
UC
PBS
200ng
400ng
SHH
F
Shh relative density
8.0
6.0
4.0
2.0
0
DMSO
20µM
40µM
DAPT
G
Ascl1a relative density
2.0
1.6
1.2
0.8
0.4
0
DMSO
DAPT
ascl1a MO+ DAPT
mmp9 MO+ DAPT

Supplementary Figure S7: Western blotting of various proteins in retinal tissue. **(A)** The western blotting performed using total retina lysate revealed the unique expected bands (marked by arrows) of various proteins used in this study. (**B-G**) Densitometry plots showing the expression of various proteins in retina, normalized to control beta actin or glutamine synthetase. $^{*}P<0.0003$, $^{**}P<0.02$.

Lissamine tagged MO	# of injected fish	# of GFP + fish
gli1	63	1
gli2	72	0
gli3	61	1
mmp9	70	0
ptch1	67	0
ptch2	75	4

Supplemental Table 1. Statistical analysis of MO injection data. Related to Figure 1, Figure 2, Figure 3, Figure 4, Figure 5, and Figure S6. The MOs used in this study are validated in embryos co-injected with MO and the GFP construct with a prefixing of MO binding sequence in-frame with GFP.
The survival statistics is given in tables.

Table S2

Gene	Wild type	Mutated
ascl1a (Mutated Gli BS)	GGGGCGTGGTCAGG	GGGGCGT**AAAA**A**A**G
	GGCGGGCCGCCGGCG	GGCG**AAAAAAAAAA**G
	CCGGAGCACCCCTG	CCGGA**AA**A**AAA**CTG
	TGAAGCCACACGTG	TGAA**AAA**A**A**ACGTG
	ACTGGGCAGTCCAA	ACT**AAAA**A**A**TCCAA
lin28 (Mutated Gli BS)	TTACACCACAGAAA	TTACA**AA**A**A**AGAAA
	GCAGTGTGATCGCT	GCAGTGT**A**AT**AAA**T
	GATGTGTGGTATTT	GA**TA**T**A**T**AA**TATTT
	TTTAGGAGGTGTGG	TTTAG**A**A**AA**T**A**TGG
	GATGTGTGGTATTT	GAT**A**T**A**T**AA**TATTT
zic2b (Mutated Gli BS)	ATACACGACGCACA	ATA**A**A**AA**ACGCACA
	AGAGACCCCAGAGA	AGAGA**AAA**AAGAGA
	GTGGGGTGCCCTGG	**A**T**A**GGGTG**AAA**TGG
	GGCGTGGGGTGCCC	**AAAA**T**A**GGGTG**AAA**
	CGTGAGGGGGCGTG	CGTGAGGG**AAAA**T**G**
foxn4 (Mutated Gli BS)	TTTGTGAGGGGTGT	TTTGT**A**A**AAAA**TGT
	TAAGTCTACCAAGG	TAAGT**AA**A**AA**AAGG
	TTAGACCACAGGTG	TTAGA**AA**A**A**A**AA**TG
	TCGGCCCTCCAGGG	T**AAAAAA**TCCAGGG
	TGTGAGGGGTGTAC	TGT**A**A**AAAA**TGTAC
foxn4 (Mutated Ascl1a BS)	CACCTG	**A**ACCTG
	CAGTTG	**A**AGTTG
ascl1a (Mutated Foxn4 BS)		
	ATAAGCGTAAA	**CCCC**GCGTAAA
mmp9 (mutated for Her4 BS)	CACAAG	**A**A**A**AAG
	CACAAG	**A**A**A**AAG
	CTGGTG	**A**T**AA**TG
	CTTGTG	CTT**AAA**

Micro RNA responsive elements

Gene	Wild type	Mutant
shha	GAGCTGTTGATATTACCACCTCT	GAGCTGTTGATATTA**A**CA**AAAA**T
	CACGACGCGACGTGTGTTTTACGTCAT	CACGACGCGACGTGTGTT**AA**A**AAAA**A**A**
	TGGCCATACCAGTTAACAAAAAATT	TGGCCATACCAGTTAACCTGCCTTT
	ATATTCAAACTGCTCCTTT	ATATTCAAACTG**AAAAAAA**
shhb	GGACGGGCAGTGGACATCACTACCTCAG	GGACGGGCAGTGGACATCACTA**AAAA**A**A**
	CACCAAGCTCACCCTCACTGCCGCGCAC	CACCAAGCTCACCCTC**AAAAAA**GCGCAC
	TGCCGCGCACCTAGTTTTCGTTGGAAACTCTTCAG	TGCCGCGCACCTAGTTTTCGTTGGAAACT**AAAA**AG
ptch1	GAATATGCACAGTTTCCCTTCTACCTCA	GAATATGCACAGTTTCCCTT**AA**A**AAAA**A
	GAGCCCATCGAATATGCACAGTTTCCCTTCTACCTCAA	GAGCCCATCGAATATGCACAGTTTCCCTT**AA**ACCTCAA

smo	CACTATGCGACTTGGAGAGCCATCA	CACTATGCGACTTGGAGA**AAA**A**AA**A
	AGTACGGCCAGCGGGTCCTGCAG	AGTACGGCCAGCGGGT**AAAA**A**A**
zic2b	CGGCGGCGCACGCTGCCTCT	CGGCGGCGCAC**AAAAAAAA**T
	GAGCGCATGCGGCGGCGCACGCTGCCTCT	GAGCGCATGCGGCGGCGCAC**AAAAAAAA**T

Supplemental Table 2. Wild type and mutated regions of various DNA constructs. Related to Figure 2, Figure 3, Figure 4, Figure 5, and Figure S6.The mutations, created on DNA sequences for disrupting transcription factor binding sites and let-7 micro RNA responsive elements of various constructs used in this study are highlighted in bold letters.

<table>
<tr><th>ZF Gene</th><th>Ensembl ID #</th><th>Position</th><th>Heteroduplex</th><th>let-7 miRNA</th></tr>
<tr><td>Shha</td><td>ENSDARG00000068567</td><td>750-772(CR)</td><td>GAGCUGUUGAUAUUACCACCUCU
|||||| | |||| |||||
UUGAUAUGUUGGAUGAUGGAGU</td><td>let-7a, b, c, d, e, f, g, h, i</td></tr>
<tr><td></td><td></td><td>1057-1083(CR)</td><td>CACGACGCGACGUGUGUUUUACGUCAU
|||||||||| || |||| |||
UU GUUGUGUUGA AU GAUGGAGU</td><td>let-7h,i</td></tr>
<tr><td></td><td></td><td>2471-2494
(3'UTR)</td><td>UGGCCAUACCAGUUAACCUGCCUU
|||||||| || || ||||||
UUGGUAUG UUGGUU GAUGGAU</td><td>let-7d</td></tr>
<tr><td></td><td></td><td>2571-2589
(3'UTR)</td><td>AUAUUCAAACUGCU CCUUU
|||||||| ||||| ||||
UUGAUAAGUUAGAUGAUGGAGU</td><td>let-7e</td></tr>
<tr><td>Shhb</td><td>ENSDARG00000038867</td><td>649-676(CR)</td><td>GGACGGG CAGUGGACAUC ACUACCTCAG
||| || ||| |||||||||
UUGAUAAGU UAGAUGAUGGAGU</td><td>let-7e

let-7c, d</td></tr>
<tr><td></td><td></td><td>1005-1032(CR)</td><td>CACCAAGCUCACCCUCACUGCCGCGCAC
|||| || ||| |||||| ||
UUGGUAUGUU GGA UGAUGGA GU</td><td></td></tr>
<tr><td></td><td></td><td>1023-1057(CR)</td><td>UGCCGCGC ACCUAGUUUUCGUUGGAAACU CUUCAG
||||||| |||| ||| |||||
UUGGUGUGUUGGA UGAUGGAGU</td><td>let-7b</td></tr>
<tr><td>Ptch1</td><td>ENSG00000185920</td><td>2590-2617(CR)</td><td>GAAUAUGCACAGUUUCCCUU CUACCUCA
|||| |||||| ||| |||||||
UUGU UGUGUU GAAUGAUGGAGU</td><td>Let-7h, i</td></tr>
<tr><td></td><td></td><td>2581-2618(CR)</td><td>GAGCCCAUCGAAUAUGCACAGUUUCCCUUCUACCUCAA
|||| ||||| ||| ||||||||
UUGG UAUGUU GGAUGAUGGAGU</td><td>Let-7b</td></tr>
<tr><td>Smo</td><td>ENSDARG00000002952</td><td>1472-1497(CR)</td><td>CACUAUGCGACUUGGAGAGCCAUCAU
||||||||||||| ||| |||
UUGAUAUGUUGGAUGA UGG AGU</td><td>let-7a, b, c, d, e, f, g, h, i</td></tr>
<tr><td></td><td></td><td>2612-2634(CR)</td><td>AGUACGGCCAGCGGGUCCUGCAG
||||||||||||| ||| ||
UUGGUAUGUUGGUUGAU GGA GU</td><td>let-7c, d</td></tr>
<tr><td>Zic2b</td><td>ENSDARG00000037178</td><td>500-519(CR)</td><td>CGGCGGCGCAC GCUGCCUCU
||||||||| ||||||||
UUGUUGUGUUGAAUGAUGGAGU</td><td>let-7h

let-7c, d</td></tr>
<tr><td></td><td></td><td>491-519(CR)</td><td>GAGCGCAUGCGGCGGCGCACGCUGCCUCU
||| |||||||| ||||||||
UUG GUAUGUUGGA UGAUGGAGU</td><td></td></tr>
</table>

Supplemental Table 4. List of genomic regions with let-7 micro RNA binding sites. Related to Figure 3 and Figure 5. The table shows a list of genomic regions of genes, mentioned in this study, with the micro RNA recognition elements (MREs) URL:https://bibiserv2.cebitec.unibielefeld.de/rnahybrid;jsessionid=be1041a93a76d436824f6e0f235b.
